# Supplementary material for: Multipurpose self-configuration of programmable photonic circuits
Source: Nat Commun. 2020 Dec 11;11:6359. doi: 10.1038/s41467-020-19608-w (PMC7733469; doi:10.1038/s41467-020-19608-w)
Supplement: Supplementary file 1 — Supplementary Information [file 41467_2020_19608_MOESM1_ESM.pdf]

Multipurpose self-configuration of programmable photonic circuits. Supplementary

Daniel Pérez López, Aitor López, Prometheus DasMahapatra and José Capmany

[1]ITEAM Research Institute, Universitat Politècnica de València, Camino de Vera s/n, Valencia, 46022, Spain.
[2]iPronics, Programmable photonics S.L., Universitat Politècnica de València, Camino de Vera s/n, Valencia, 46022, Spain.

Index

Supplementary Note 1: Architecture, variable space and cost function definitions for the optimization process
Supplementary Note 2: Pre-characterization routines of waveguide meshes
Supplementary Note 3: Auto-routing algorithm
Supplementary Note 4: Optimization methods employed at a glance
Supplementary Note 5: Self-configuration of Optical beamsplitter 1x8 channels
Supplementary Note 6: Self-configuration of All cross function, an arbitrary routing example
Supplementary Note 7: Self-configuration of optical filters
Supplementary Note 8: Description of the laboratory set-up
Supplementary Note 9: Experimental synthesis of optical circuits in a 30-TBU waveguide mesh
Supplementary Note 10: Main challenges for large-scale programmable photonic circuits
Supplementary Note 11: Examples of recent demonstrations of reconfigurable waveguide mesh arrangements

Supplementary Note 1: Architecture, variable space and cost function definitions for the optimization process

The self-configuration algorithms proposed in this paper rely on the application and customization of optimization routines employed in a wide range of application fields. Optimization deals with the task of finding the optimal values for the variables of a system to maximize or minimize its output. An ideal optimizer should avoid stacking in local optima and explore efficiently the variable's space to find the global optimum point as well as converge efficiently when this is found.

In most of the applications and algorithms demonstrated in this paper, the system to be optimized is the general-purpose photonic integrated circuit (PIC) based on a waveguide mesh arrangement. We consider the PIC as a black box whose response is given by the full scattering matrix of the circuit. The scattering matrix contains the spectral response of every optical port combination. Although a real system has an amplitude and phase response, we will employ the overall amplitude response in in our application examples. As shown in Supplementary Figure 1, the overall system can be modified through the application of a set of electrical signals that modify the optical properties locally in the circuit. This set of variables is defined by the vector $\boldsymbol{v}$. When dealing with a real system this vector can represent the electrical signal feeding each phase actuator or photonic actuator in general. In our results, we demonstrate some of the concepts employing a performance estimator that includes a model of the non-ideal performance of every component [1] as well as the experimental self-configuration employing a real system. In both cases, the initial, environmental conditions and the operating conditions of the circuit modify the system performance and are considered to be unknown and random.

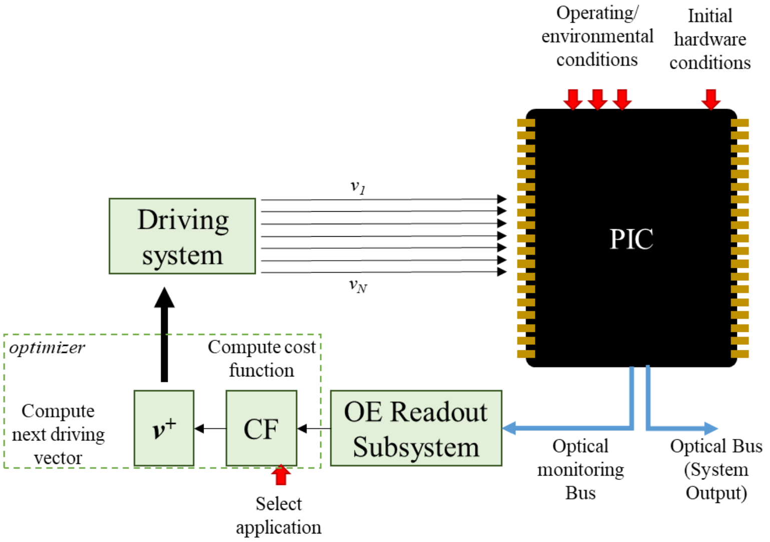

Supplementary Figure 1 | Optimization system diagram and their application to self-configuring performance of optical processors. CF: Cost function, v: vector defining the configuration variables of the system for the integrated actuators, PIC: Photonic integrated circuit. The full cycle define an operation.

In this work we apply both stochastic and derivative optimization techniques. In all cases, the optical system response is given by the settings applied to each phase actuator (***v***), the initial hardware conditions such as non-uniform loss distributions per TBU in the system as well as the passive conditions (Cp). The latter includes the phase offset, environmental conditions, and hardware-related non-perfect performance mostly given by nanometric fabrication errors. For each wavelength, we obtain the scattering matrix of the circuit that represents the optical response for every input and output port combination as *So,i*.

$$S\left(v,\lambda,Cp\right)=\begin{pmatrix} S_{11} & S_{12} & \cdots & S_{1P} \\ S_{21} & \ddots & & \vdots \\ \vdots & & \ddots & \vdots \\ S_{P1} & \cdots & \cdots & S_{PP} \end{pmatrix}_{\lambda} = f\left(v,\lambda,Cp\right) \quad (1)$$

With the scattering matrix information, or a portion of it, we compute the cost function. As shown in the Supplementary Figure 1, this function depends on the application to be optimized. A process of cost-function engineering requires the search of a function that is minimized when the targeted application is achieved. Assume for example that we want to route the light from port 1 to port 8 of a certain circuit. A valid cost function would be the negative value of the optical power received in this optical channel $S_{8,1}$. After an optimization process, we would maximize the optical power inside of this optical path. Once computed the cost function, the optimization algorithm computes the next vector of variables for the driving system. Thus, the optimizers deal with finding the optimum values for the individual phase actuators in the circuit to minimize a cost function and get the desired response of the optical processor, even in the presence of non-ideal conditions. The full cycle of driving, and monitoring is defined as an operation along this paper. Some optimization algorithms require the computation of a certain number of operations per iteration, so we re-scale to number of operations to compare their performance.

Supplementary Note 2: Pre-characterization routines

Due to fabrication errors, a minimum change in the waveguide geometry impacts on the propagation of the optical signal. This, together with design errors, induces phase deviations with respect to the phases predicted during the design stage. Precisely, although theoretically each Tunable Basic Unit (TBU) should be in a fixed state (typically cross or bar), phase errors in the 3-dB couplers and in the waveguide sections of the MZI as well as its accesses introduce changes in the phase response that derive in a random coupling and phase under passive (non-driven) conditions. This issue implies that the electrical power required to set a TBU into cross or bar is unknown and different for each TBU. In addition, yield errors during fabrication can produce non ideal effects like additional optical loss across distinct locations of the circuit.

Although most of the algorithms presented in this paper do not require a pre-characterization routine of the waveguide mesh arrangement, we also presented a set of configuration algorithms that require a close estimation of the performance of each component. This information is employed to make optimum configuration decisions. For example, under this category we presented algorithms based on pre-sets and routines performing auto-routing functions. As a first stage, both of them require information such as the insertion loss of every TBU, the power consumption for each coupling state and the phase shifter calibration versus electrical driving to cite a few. There are different methods to obtain this data automatically:

-Local monitor system at every TBU:

The presence of opto-electronic readouts at every TBU port is a brute-force approach to obtain the characterization of every TBU. They can be used to run local algorithms that through the optical power gathered at both outputs, obtain an estimation of the coupling state of the unit. Although included in the list, this approach requires an excessive overhead in the electronic and control circuit, limiting the scalability of the overall system.

-Self-characterization of the TBU insertion loss:

To estimate the insertion loss of each TBU, only optical power monitors placed at the external perimeter of the waveguide mesh are required. In such case, pre-characterization routines can be implemented by building up a linear system of equations describing a number of optical interconnections greater than the number of existing TBUs. A larger number helps to reduce the statistical noise arising from fiber-chip coupling loss variances. The easiest way to define such system of equations automatically consists on using an auto-routing algorithm (whose discussion can be found in the **Supplementary Note 3**) to create paths connecting arbitrary pairs of outer nodes from the waveguide mesh.

Once provided with a sufficiently large number of paths, we can compute or measure the optical loss per channel and translate them into a sparse matrix of connections of dimension MxN (being M the overall number of included paths and N representing the number of TBUs in the mesh) with '1's in those elements within each row whose corresponding TBUs are under use to form the corresponding path. Supplementary Figure 2(a-c) shows an illustrative numerical example of this process. To obtain the results from Supplementary Figure 2(c), we have considered two different noise sources to simulate a more realistic scenario in the determination of the ILs. The first noise source, defined by a uniform distribution with mean $\mu_1 = 0.005\pi$ and variance $\sigma_1 = 0.03\pi$, describes a possible variation in the determination of the phase shifting values of each actuator. The second one, described by a second uniform distribution with mean $\mu_2 = 0.2$ and variance $\sigma_2 = 0.1$, would correspond to any possible misalignment while measuring the overall IL of each path. After the addition of sufficiently large number of synthesized channels, we compute the difference between the estimated insertion loss for each individual TBU and the real value. We observe how the estimation error of the individual IL stabilizes after measuring and processing 36+15 additional optical paths. Under this scenario, the estimation error converges to 0.18 dB approximately.

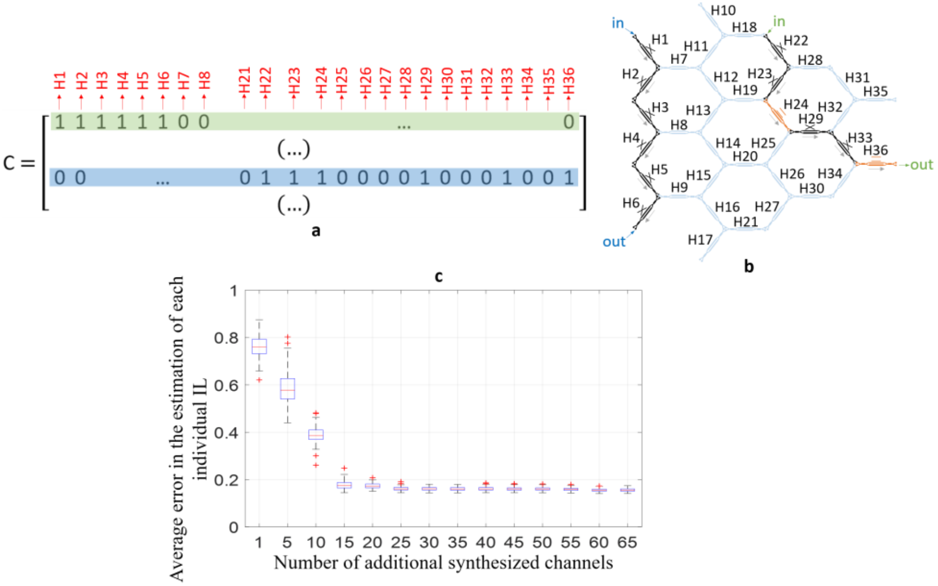

Supplementary Figure 2 | Self-characterization of the TBU insertion loss: (a) system of equations to estimate the loss per TBU. (b) waveguide mesh arrangement involving 36 TBU and two examples of paths describing connections to the outer ports. (c) Average error bar plot in the estimation of each individual IL versus the number of additional synthetized channels

The scalability of the method might be compromised when scaling up the waveguide mesh arrangement. Although the number of TBU increases faster than the number of optical ports, the number of optical paths that we can measure to perform the linear matrix inversion also grows exponentially, enabling the creation of the matrix. The evolution of the method and its relation to the number of unit cells in the circuit and is currently under study. However, preliminary results show that circuits with 80- 200 TBUs achieve the self-characterization method but require additional time for the pair-ports matrix generation.

-Self-characterization of the TBU driving calibration:

Obtaining the calibration (electro-optical mapping) of every phase shifter from a large-scale waveguide mesh arrangement requires the assistance of an automated process. There are different strategies to achieve the goal:

Self-characterization of the TBU electro-optical mapping based on max-min methods: This approach consists of the placement of few optical readouts and optical source points in the external ports of a waveguide mesh arrangement. Using a sequential routine that

maximize the optical power at the channels, starting from the shorter paths, it is possible to achieve the desired mapping for each TBU [2]. This method reduces the amount of optical readouts when compared with the brute-force approach that integrates a monitor per TBU.

Self-characterization of the TBU electro-optical mapping based on the all-cross function: The previous approach works smoothly, only requiring a reduced number of ports with optical sources and other ports with a small set of readout points. However, it might require few full-cycle iterations to obtain a clean and precise mapping for each TBU when dealing with large-scale meshes. An alternative is the use of the *all-cross* function described in the main document and in **Supplementary Note 6** as a sub-routine in the *calibration and characterization* function. The routine workflow is as follows: first, the *all-cross* routine is employed, perfectly defining closed light-paths in the waveguide mesh arrangement to avoid feed-back loops all over the circuit. Then, for each optical channel, an iterative process starts for each actuator inside the path. The process consists of the electrical tuning of one phase actuator while the system reads one optical power readout to accurately identify the driving conditions to achieve the *bar state*. Also, one can monitor a set of electrical powers at the driver and their associated optical powers at the readout to map a function to obtain the coupling ratio curve by sampling and fitting a small number of points to the well-known sinusoidal MZI equation to the zeros of the function. If the *all-cross* function is not employed at the beginning, the iterative process is less efficient, noisy and requires more iterations to succeed since the optical power leakage in the waveguide mesh arrangement increase the number of undesired optical paths and thus, the uncertainty of the calibration procedure, [2].

Self-characterization of the tuning crosstalk: When tuning one phase actuator, for example a thermo-optic actuator, the physical effect causing the tuning in the desired waveguide can spread to the neighbouring waveguides producing an undesired tuning effect. In the worst cases, even at distances larger than 10 mm the tuning crosstalk effect can be appreciated [3]. The tuning crosstalk can be modelled by a constant that reflects the percentage of phase shift occurred in the non-targeted waveguide compared to the experienced by the target waveguide [4]. Simulations and experimental works result in a crosstalk coefficient between 1 and 3% at several hundreds of micrometres. If extended to a system with multiple phase shifters, this model can be extended to a system of equations relating the effective phase shifts with the phase shifts set by the algorithm or the user.

$$\Delta\phi_{effective} = \begin{pmatrix} 1 & CT_{12} & \cdots & \cdots & CT_{1N} \\ CT_{21} & 1 & & & CT_{2N} \\ CT_{31} & & 1 & & \vdots \\ \vdots & & & \ddots & CT_{1N-1} \\ CT_{N1} & CT_{N2} & \cdots & \cdots & 1 \end{pmatrix} \begin{pmatrix} \Delta\phi_1 \\ \Delta\phi_2 \\ \vdots \\ \\ \Delta\phi_N \end{pmatrix} \tag{2}$$

Some circuits configurations have been demonstrated using open control loops requiring the pre-characterization of the tuning crosstalk matrix [5, 6] . However, it is not yet well studied the stability of this matrix in large-scale circuits with high-integration densities, since the gradient of the temperature is still present at long distances and it is also dependent on the modulation speed.

Supplementary Note 3: Auto-routing algorithm

The rationale behind this approach is to employ the well-known strategies of graph theory and pathfinding algorithms to configure interconnections and optical delay lines in a FPPGA core. This configuration optimizes the optical interconnection or programmed waveguide as a function of a given set of parameters with competing objectives such us reducing the accumulated loss, reduce the power consumption, reduce the number of resources employed, etc. The algorithm is fed initially with all the information gathered during a pre-characterisation routine as the ones described in **Supplementary Note 1**. In addition, it requires access to the graph emulating the physical interconnection of the waveguide mesh hardware. Different graphs have been proposed, extending configuration features [7] and reducing the customization process of the algorithms already available in the field [8].

Some key concepts in graph theory and their adaptation to waveguide mesh-based photonic integrated circuits.

- Graphs, the fundamental edges of study in graph theory, are systems of nodes connected in pairs by edges. The nodes can be defined by the physical optical ports of the TBUs and the edges represent the inner connections between the TBU ports.
- Weights are numerical values assigned to graph edges. The overall weight of any path inside the graph (i.e., a walk with or without repeated vertices and consequently edges) can be given by the sum of the weights of the edges within such path. In this work, the weights are defined as the performance parameter to be optimized during the creation of the optical connection or delay line (insertion loss, power consumption, basic unit length, basic unit delay, etc.)

A proposed pseudocode to achieve this task can be found here [7]. As a preliminary step, the algorithm runs a process to index all the TDs from a list of nodes describing the circuit interconnections. This aids in impeding that a path traverses through a given TBU twice consecutively and showing backward direction. The algorithm then creates the graph framework from this ordered list of nodes and TDs and sets the accumulated distance from the initial node to itself as zero and to all the others as infinity. From then on, a shortest path tree with the input port as root propagates through the remaining ones in the graph by accumulating each TD prior to reaching the destination port. Similar to original Dijkstra's implementation, the paths that go through the same node more than once during the process are discarded and the rest are stored inside the 'paths' variable. Once the destination port has been reached, the resulting paths and the

process keeps running until a fixed number of paths arrives to destination. An application example of this technique is illustrated hereunder in Supplementary Figure 3, in which we proceeded to synthesize an optimized optical path with respect to several of the aforementioned figures of merit and an imbalanced 2-TBU MZI. For this last family of circuits and optical filters in general, this algorithm allows an agile and optimized connection between the tunable couplers taking part of such kind of structures.

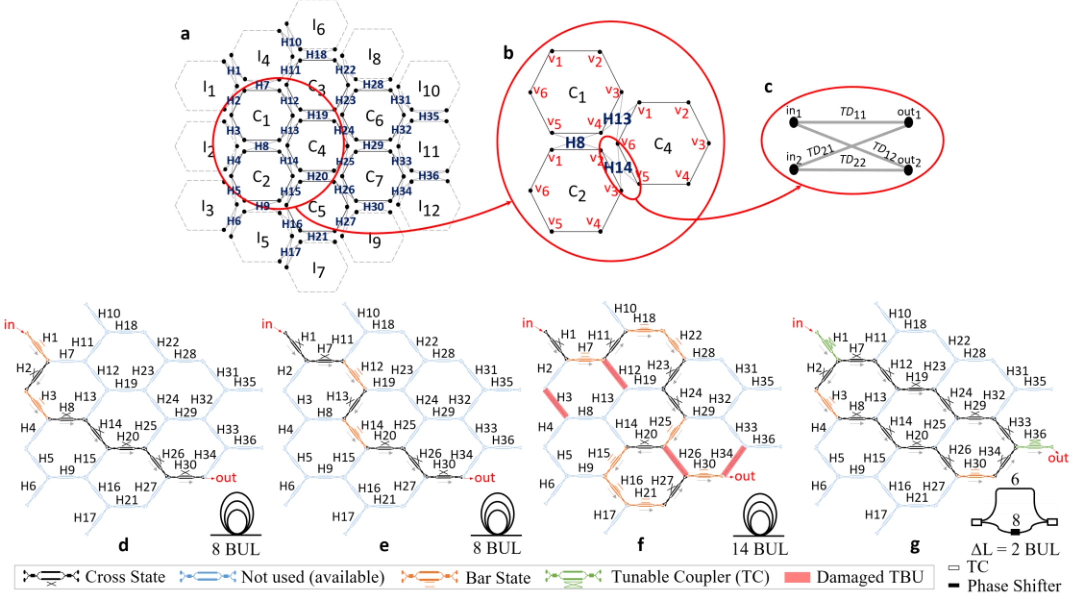

Supplementary Figure 3 (a) Graph representation of a 36-TBU waveguide mesh. TBUs, actual and imaginary cells are numbered from top to bottom and from left to right. Graph nodes within each hexagonal cell are numbered clockwise and hereinafter referred to as C(/I)xvy, where Cx or Ix represents the actual (x = {1, 2, …,7}) or imaginary cell (x = {1, 2, …,12}) in which the node is located, (b) node representation where vy denotes its position inside a cell (y = {1, 2, …,6}), (c) internal connections of the TBU where TD: Transmission distance stands for the weight or cost to travel from one node to another, (d) Synthesis of an optical path, optimized with respect to the number of traversed TBUs (8), (e) Re-synthesis of previous optical path optimized with respect to overall power consumption, featuring a power consumption improvement of 16.57% according to experimental data, (f) Demonstration of self-healing and fault-tolerant capability, assuming a malfunction in TBUs H3, H12, H26 and H30, (g) Synthesis of a 2-TBU imbalanced MZI using the auto-routing algorithm as sub-routine to configure both upper and lower arms.

One of the main virtues of this algorithm is its capability to provide self-healing and fault-tolerant attributes to the waveguide mesh. Provided that a local impairment, such a TBU malfunction, takes place in a very specific part of the photonic arrangement, the algorithm is be able to take it into account and search for alternative paths without crossing it to arrive to destination port. To do so, the algorithm would need to be provided with the individual estimated IL of each TBU in a periodic basis, following the aforementioned routing from **Supplementary Note 1**. A second application example of this feature can be observed in Supplementary Figure 4, which also illustrates the synthesis of multiple optical channels at a time. For this example, we are using a waveguide mesh of a larger size including 81 TBUs. To do so, we first focus on the synthesis of the first optical interconnections and, once finished, set all the TDs corresponding to opposite transmission states for each of its constituting TBUs to infinity. Once finished, we can run the algorithm a second time to create new structures while maintaining the previous ones. Supplementary Figure 4(b), illustrates the configuration example when some of the TBUs (H5, H40, H41, H50, H67 and H76) are identified as damaged during the pre-characterization stage. As observed, nothing prevents the algorithm to work efficiently for different waveguide mesh sizes, or even for different mesh topologies [7].

A potential issue arising from this implementation is the characterization of the tuning crosstalk. When the circuit contains a large number of photonic actuators, the auto-routing routine is not able to mitigate this effect and its performance decreases severely. A potential solution is the combination of the auto-routing method followed by the application of computational optimization methods covered in this work, considering only the optimization of the phase shifters included in the path given by the auto-router.

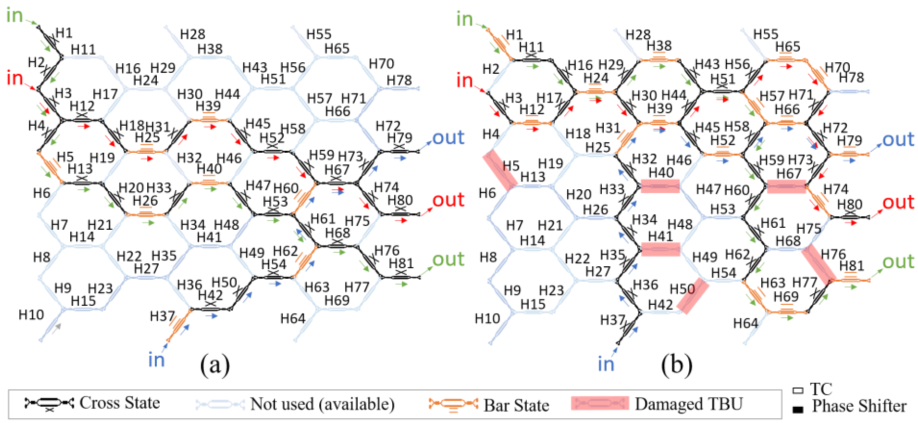

Supplementary Figure 4 | Application example of the auto-routing algorithm to an 81-TBU waveguide mesh arrangement: (a) Multiple input multiple output routing example, (b) the same scenario but with the identification of a selected set of TBU with high insertion loss (damaged) before the algorithm application.

Supplementary Note 4: Optimization methods employed for the self-configuration routines at a glance

In this work we demonstrated the use and customization of both stochastic and derivative optimization methods and routines for the self-configuration of multipurpose waveguide mesh arrangements and programmable photonic processors. The stochastic algorithms optimize problems employing random distributions at some point during the process. In this work we applied simple implementations of a genetic algorithm with scheduling and a particle swarm optimization, although both individual-based and population-based algorithms can be employed, as well as their evolutions. This work thus shows the self-configuration nature of a general-purpose waveguide mesh arrangement and sets the basis for the full exploration of advanced computational methods and optimization algorithms. In addition, we also explored the use of derivative methods requiring the computation of the gradient. This supplementary note explains briefly the hyperparameters involved for tuning the optimization methods and summarizes their implementation. This supplementary note aims to extend the section covering the methods and follows the diagram explained in **Supplementary Note 1**. We have implemented them employing a Python-based custom code.

Non-derivative computational optimization methods:

*Genetic algorithms***,** also known as evolutionary algorithms, resemble natural selection and reproduction processes governed by rules that assure the survival of the fittest individuals in large populations [9]. Individuals (points) are associated with identity genes that define a fitness measure (objective function value). A set of individuals form a population, which adapts and mutates following probabilistic rules that utilize the cost function. In this case, our individuals are defined by ***v*,** as defined in **Supplementary Note 1**. For each generation, we define the number of points given by the first hyperparameter: *population_percentage_per_weights*. Each point is a vector ***v*,** (initially random). Once computed the fitness (or cost function) we select a set of the fittest (individuals with reduced *CF*). The selection procedure can vary for different approaches like Boltzmann selection, Tournament selection, Rank selection, Steady state selection to cite a few. In our case, we use the simplest approach that consist of selecting a percentage of the best individuals, given by the hyperparameter *num_matting*. Next, we combine the remaining population and obtain the same number of points as we have before the selection process. After the mating process, the samples will mute. Although multiple alternatives exist for the muting process, we employ again the simplest approach which consist of modifiying a percentage of the weights given by the hyperparameter *num_mutations_per_weights_percent* following a uniform distribution with a limit imposed by a variable that suffers an exponential decay scheduling and with an initial value given by the hyperparameter *init_mutation_range_per_pi*. In short, this algorithm requires the tuning of 5 hyperparameters.

Since the optimum hyperparameter selection depends on the cost function employed, and the application, for each application we performed a preliminary test to check the impact of each hyperparameter on the self-configuration task. This task is also helpful as a first exploratory search of the optimum hyperparameter combination. The hyperparameters, when indicated, are selected following the ranges specified in Supplementary Table ***1***.

Supplementary Table 1 | Grid-search of the hyperparameters employed by the genetic algorithm

Population *(as a function of the number of weights)*	*Parents Mating* *(as a function of the population)*	*Number of Mutations*	*Mutation value from uniform distribution.* *(normalized to π)*	*Exp. Decay Mutation*
300%	50%	50%W	50%	0

200%	20%	10%W	10%	0.5
100%	10%	5%W	1%	0.7
50%			0.1%	

Particle swarm algorithm (PSO) is a population-based algorithm that maintains at each iteration a swarm of particles (set of points) with a velocity vector associated with each particle [10]. At each iteration, it generates a new set of particles from the previous swarm combining random and inherited parameters (*inertia*, cognition, and *social*). It is typically classified as a global-search algorithm. In our case, each particle position is defined by the driving vector ***v*** as described in **Supplementary Note 1.** The hyperparameters to be tuned are the *number of particles in the swarm*, the *inertia*, the *cognition* and the *social* coefficients. The inertia represents the momentum and speed of the particle. At each iteration, all the particles share the information regarding the position of the best positioned particle and its cost function. Thus, the *cognition* coefficient models the weight of the decision made by a particle considering his own track record. The *social* coefficient models the decision of each particle considering the best positioned particle in the group.
The efficiency of the PSO algorithm is dependent on the hyperparameters selected, the cost function and the targeted problem. Like for the genetic algorithm, for each application we perform a grid-search tuning the hyperparameters and running the algorithm for 320 combinations for each of the proposed cost functions. The hyperparameters are selected following the ranges specified in Supplementary Table . We selected wide ranges to ensure that we explore different combinations.

Supplementary Table 2 | Grid-search of the hyperparameters employed by the particle swarm optimization algorithm

Number of particles per weight	Inertia	Cognitive	Social
300%W	2	2	2
200%W	1	1	1
100%W	0.5	0.5	0.5
50%W	0.1	0.1	0.1
20%W			

As in the genetic algorithm, wherever specified, we performed a scheduled decrement of the *inertia* coefficient from the value specified in the grid until a fixed coefficient of 0.35, describing a linear decrement until arriving to iteration operation 1000. This scheduling improves the convergence to the optimum value as reported in this work.

Derivative optimization methods: For the minimization of the cost function, these set of techniques employ the multivariable generalization of the derivative of the CF for each variable in ***v*** [11]. The resulting vector ***g*** is the gradient of the function and it provides the direction tangential to the error surface at the evaluation point defined by **v**:

$$\mathbf{g} = \nabla_{\mathbf{v}} CF(\mathbf{v}). \tag{3}$$

This direction is employed to advance on the opposite way to progress in the minimization of the error function. A wide range of first-derivative optimization methods are reported in the literature [12].
A straightforward approach for computing the derivatives of the error function is to use finite differences approximation. This can be done by perturbing each variable in turn, and approximating the derivatives by using one of the following expressions:

$$g_i = \frac{CF(v_i + \varepsilon) - CF(v_i)}{\varepsilon} + O(\varepsilon), \tag{4}$$

$$g_i = \frac{CF(v_i + \varepsilon) - CF(v_i - \varepsilon)}{2\varepsilon} + O(\varepsilon^2), \tag{5}$$

In (3-5) the gradient employs the evaluation of the CF in **v** perturbing the position *i* by a small amount ε. In our case, we define ε equal to 0.3 10^{-3} rads. The rationale behind is that the finite-differences approximation is better approximated if we are close to the evaluation point. However, the lower limit of ε will be imposed by the resolution of our electrical drivers and the noise of the readout system. In (5), we see the central differences equation to get a significantly better approximation of the gradient. However, the number of computational steps is almost doubled when compared to (4). As the reader can infer, getting the gradient straightforwardly in a real waveguide mesh system, as proposed, implies performing the perturbation of one actuating variable, getting the associated CF from the postprocessed signal from the readout system, repeating the procedure with a negative perturbation and then computing (3). Other variations update and employ the updated gradient during the computation of each position of the gradient per se.

Gradient descent is the simplest and more extended first-derivative optimization algorithm. It renders the next configuration state of our system settings by sequentially applying:

$$\mathbf{v}^t = \mathbf{v}^{t-1} - \eta\mathbf{g}, \tag{6}$$

where η is the learning rate or step size.

Gradient descent with momentum can be employed to enhance the convergence speed, and to overcome noisy gradients. It accumulates an exponentially decaying moving average of past gradient vectors $\mathbf{v}_{mo}$ and use a proportion (α_{mo}) of it to set the new direction. The new $\mathbf{v}^t$ is

$$\mathbf{v}^t = \mathbf{v}^{t-1} + \left(\alpha_{mo}\mathbf{v}_{mo} - \eta\mathbf{g}\right), \qquad (7)$$

where α_{mo} is another hyper-parameter in the range [0,1) that determines the relation between the new gradient and the accumulated. Other variations like conjugate gradient descent can be employed.

Root Mean Square Propagation (RMSProp) is a derivative method tailored for machine learning optimizations where the gradient magnitude is different for each variable. Thus, it employs only the sign of the gradient and adapts over time the step size individually for each variable dimension. It can be classified as a global search algorithm.

Adaptive moment estimation (Adam) is an optimization algorithm that also adapts the parameter learning rates individually. However, rather than using exclusively the average first moment (the mean) as in RMSProp, Adam also makes use of the average of the second moments of the gradients (the uncentered variance).

Supplementary Note 5: Self-configuration of optical beamsplitter 1x8 channels

In this example, the aim is to configure a set of phase actuators in the waveguide mesh arrangement to program the operation of a beamsplitter with one specified input port and 8 specified output ports to achieve equally splitting the input port optical power. This functionality is particularly interesting for multiple signal processing schemes and subsystems, like finite impulse response filters and beamforming networks [13] [14] [15]. The proposed cost functions are presented in Supplementary Equation 8, where we have incorporated two features dealing with the error between the average optical power budget between the eight channels and the expected value and with the average ripple in the targeted channels [11]. For comparison, we consider two different *Cost Functions ($CF_{1,2}^{1x8}$)*:

$$CF_1^{(1x8)}\begin{cases} c_1 = 1/8, \quad c_2 = 1/8, \\ f_1 = \sum_{op}\left(10\log_{10}\left(\left|H_{op,6}\right|^2\right)+10\right)^2, \\ f_2 = \left(\begin{array}{l}\sum_{op}\max\left(10\log_{10}\left(\left|H_{op,6}\right|^2\right)\right) \\ -\min\left(10\log_{10}\left(\left|H_{op,6}\right|^2\right)\right)\end{array}\right), \end{cases} \quad CF_2^{(1x8)}\begin{cases} c_1 = -1, \quad c_2 = -1 \\ f_1 = \frac{20}{N}\sum_{1}^{chs.}\left(\log\left(1-\frac{\left|\left|H_{chs.}\right|-0.31\right|}{0.69}\right)\right), \\ f_2 = 20\log_{10}\left(1-\frac{1}{N}\sum_{1}^{chs.}\left(\begin{array}{l}\max\left|H_{chs.}\right| - \\ \min\left|H_{chs.}\right|\end{array}\right)\right), \end{cases} \qquad (8)$$

where *op* references the optical ports under use by this configuration. Note that, in this case, we are not employing the information coming from the signals from the non-targeted ports to reduce the number of reads by a practical readout system. In both cases, we consider the average signal in the targeted optical channels and the ripple at the channel. This is an example of a cost function that employs spectral information, meaning that if low-speed diodes are employed a laser sweeping multiple wavelengths would be required in a real system implementation. Alternatively, a filtered WDM spectrum could be photodetected at each spectral channel, increasing the complexity of the system. The use of extra features and the consideration of non-used or secondary ports is particularly interesting for larger-scale waveguide meshes. Supplementary Figure 5 illustrates both the labelled waveguide mesh employed in this example together with a schematic view of the power splitting from port 12 to channels 6,4, 3, 2, 1, 24, 23 and 21. A similar example as the CF1 was addressed in a recent publication [11].

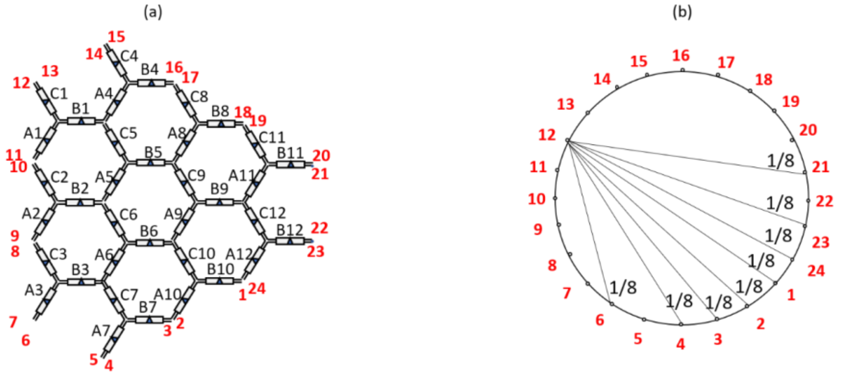

Supplementary Figure 5 (a) Labelled schematic of the waveguide mesh arrangement under test. (b) black box system with the targeted performance: Routing between channels defined by the port pairs with input 12 and outputs 21, 23, 24, 1, 2, 3, 4, 6.

To compare between different cost functions and models, that can make the optimization results quite non-intuitive, during the process we define and keep track of two output features at every iteration. The first one, referred as *Output Feature* 1 (OF_1) computes the mean optical loss at each channel and is normalized to -10 dB. The second one, (OF_2) captures the mean ripple at the 8 targeted channels (dB).

Data analysis for the different advanced optimization methods for the Optical beamsplitter configuration:

- **Optical beamsplitter 1x8 channels self-configuration employing a Genetic Algorithm**

The efficiency of the genetic algorithm is dependent on the hyperparameters selected, the cost function and the targeted problem. The summarized explanation of the algorithm employed in this work can be found in **Supplementary Note 4**. In order to test the performance and to find the best ranges for the hyperparameters for the optical beamsplitter problem, we performed a grid-search tuning the hyperparameters and run the algorithm for 409 trials for each of the two proposed cost functions. The results were tracked and saved for posterior analysis and postprocessing in a datasheet. The hyperparameters were selected following the ranges specified in Supplementary Table 1, ensuring wide-enough ranges to explore different combinations. For each hyperparameter combination we run an independent self-configuration process, where the passive conditions of the mesh are selected from a random distribution and remains unknown during the whole process. Finally, to ensure a fair comparison between the different combination of hyperparameter, a maximum number of 3000 operations is allowed during the iterative process, independently of the size of the population selected.

CF^{1x8}_1:

Our first analysis consists of computing the correlation matrix of the datasheet including the data gathered during the self-configuration process. It includes the final cost function, the output features and the values of the hyperparameters. As a result, it returns a coefficient describing the linear correlation of the data. Although restrictive to linear correlations, it is a good first-search indicator. For example, Supplementary Table suggests that the *init mutation range per pi* and the *mutation_decay* value have a higher negative correlation with the targeted cost function minimization. A deeper analysis of the data reveals that the optimum value for the *mutation_decay* coefficient is related to the *init_mutation range per pi*, suggesting that if it starts being too small, the decay is counterproductive and the process get stacked into local minima points.

Supplementary Table 3 | Correlation matrix of the datasheet after the grid-search application of a self-configuration routine based on the genetic algorithm approach.

**(CF^{1x8}_1)**	**CF**	**OF1**	**OF2**
**CF**	1.000000	0.958451	0.649870
**OF1**	0.958451	1.000000	0.637895
**OF2**	0.649870	0.637895	1.000000
mutation_decay	0.155327	0.152923	0.010581
parents_mating_per_population_percent	-0.057200	-0.023768	-0.034353
mutationpermating	-0.108323	-0.173077	-0.131097
parents_mating_per_num_weights_percent	-0.204237	-0.182081	-0.034353
population_per_num_weights_percent	-0.270537	-0.260325	-0.119533
num_mutations_per_num_weights_percent	-0.304063	-0.347558	-0.175940
mutation_decay vs range	-0.371618	-0.378442	-0.305843
init_mutation_range_per_pi	-0.448689	-0.460422	-0.300384

To delve into the actual performance of the different combinations, Supplementary Figure 6 and Supplementary Figure 7, illustrate the histogram of the complete datasheet and the datasheets including only the combinations of hyperparameters that achieve a performance closer to the targeted one, respectively. The second datasheet includes thus the best results accomplishing OF_1 better than 3 dB and OF_2 better than 2 dB. From the data, we infer that the *init_mutation_range_per_pi* value should be close to 50%. A lower value limits the exploration capability of the algorithm in favor to the exploitation capability, increasing the probability to get stacked in a local minimum. In addition, for larger values of *init_mutation_range_per_pi*, the best *mutation_decay* is 0.5. The value of *num_mutations_per_num_weights_percent should be close to 50%. The parents_mating_per_num_weights_percent* should be close to 10 and 20 %.

From the data we obtain that the best ranges are:

Supplementary Table 4 | Selection of the best hyperparameter combinations after the grid-search for the application of a genetic algorithm in the self-configuration of a 1x8 optical beamsplitter.

**(CF^{1x8}_1)**	**Best performance ranges**
mutation_decay	0.5
parents_mating_per_num_weights_percent	20%
population_per_num_weights_percent	any
num_mutations_per_num_weights_percent	50%
init_mutation_range_per_pi	50%

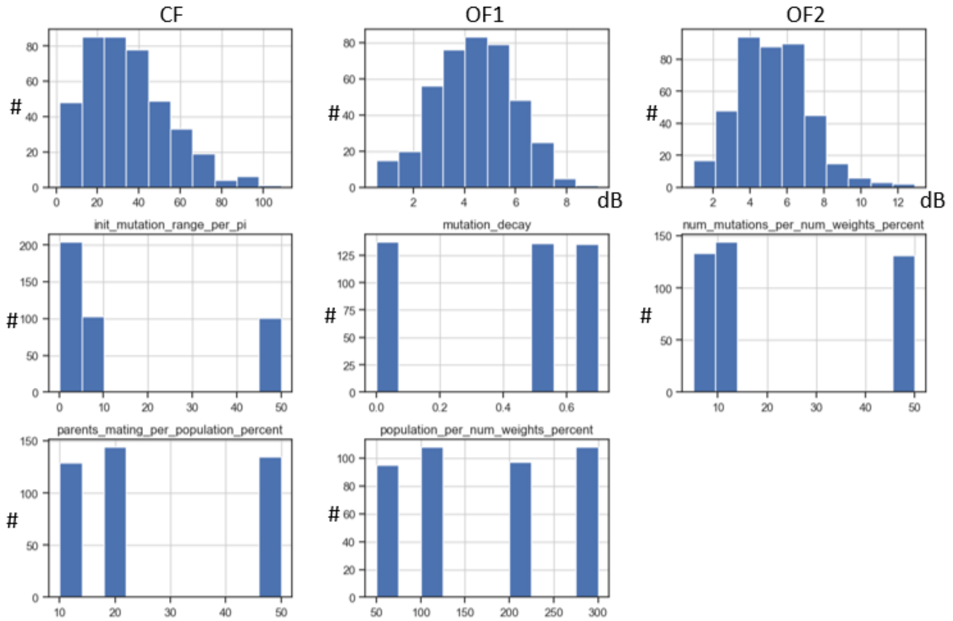

Supplementary Figure 6 | Optical beamsplitter 1x8 grid search results with genetic algorithm for CF^{1x8}_1 : Histogram including all datasheets.

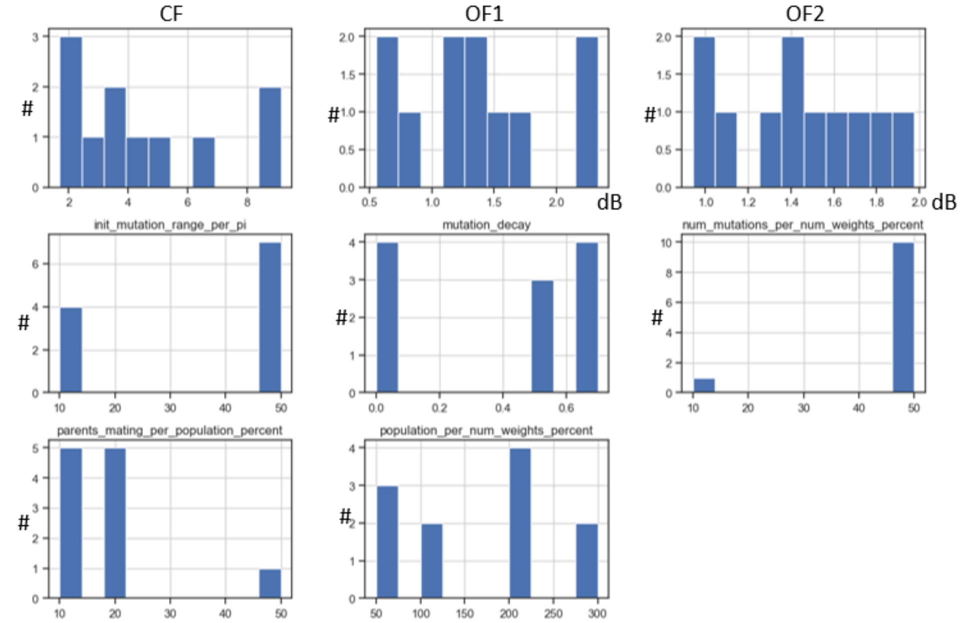

Supplementary Figure 7 | Optical beamsplitter 1x8 grid search results with genetic algorithm for CF^{1x8}_1: Histogram including the best performance datasheets.

From this preliminary analysis, the 2.6 % of the samples achieve the targeted metrics for the output features simultaneously. This low convergence reflects both the dependence of the hyperparameter values with the completion of the task and that the hyperparameter ranges selected are too broad. We limited the number of operations to 3000 for every trial, so slower convergency samples might be considered as failed. It is worth nothing that each operation implies a single configuration of the waveguide mesh and the extraction of the amplitude scattering matrix at the 8 output channels.

***CF^{1x8}_2*:**

We repeat the previous experiment but employing a CF^{1x8}_2. The results of the correlation of the datasheet are covered in Supplementary Table :

Supplementary Table 5 | Correlation matrix of the datasheet after the grid-search application of a self-configuration routine based on the genetic algorithm approach

**(CF^{1x82})**	**CF**	**OF1**	**OF2**
**CF**	1.000000	0.915892	0.327019
**OF1**	0.915892	1.000000	0.513217
**OF2**	0.327019	0.513217	1.000000
mutation_decay	0.077110	0.085830	0.074630
parents_mating_per_population_percent	-0.007362	-0.061426	0.026494
mutationpermating	-0.110021	-0.065654	-0.040172
parents_mating_per_num_weights_percent	-0.148215	-0.216105	-0.046298
population_per_num_weights_percent	-0.227142	-0.269435	-0.078157
num_mutations_per_num_weights_percent	-0.273857	-0.244912	-0.108319
mutation_decay vs range	-0.451101	-0.375154	-0.067322
init_mutation_range_per_pi	-0.527120	-0.475107	-0.141418

To delve into the actual performance of the different combinations, Supplementary Figure 8 and Supplementary Figure 9, illustrate the histogram of the complete datasheet and the datasheet, respectively. As in CF^{1x8}_1, from the data, we infer that the *init_mutation_range_per_pi* value should be close to 50%. Again, for larger values of *init_mutation_range_per_pi*, the best *mutation_decay* is 0.5. The value of *num_mutations_per_num_weights_percent should be close to 50%. The parents_mating_per_num_weights_percent* should be close to 10 and 20 %. From the data we obtain that the best ranges, presented in Supplementary Table . Note that the best hyperparameters are similar for the ones selected for the CF^{1x8}_1.

Supplementary Table 6 | Selection of the best hyperparameter combinations after the grid-search for the application of a genetic algorithm in the self-configuration of a 1x8 optical beamsplitter.

**(CF^{1x8}_2)**	**Best performance ranges**
mutation_decay	0-0.6 (proportional to init mut range)
parents_mating_per_num_weights_percent	20-25%
population_per_num_weights_percent	200-300%
num_mutations_per_num_weights_percent	50%
init_mutation_range_per_pi	10-50%

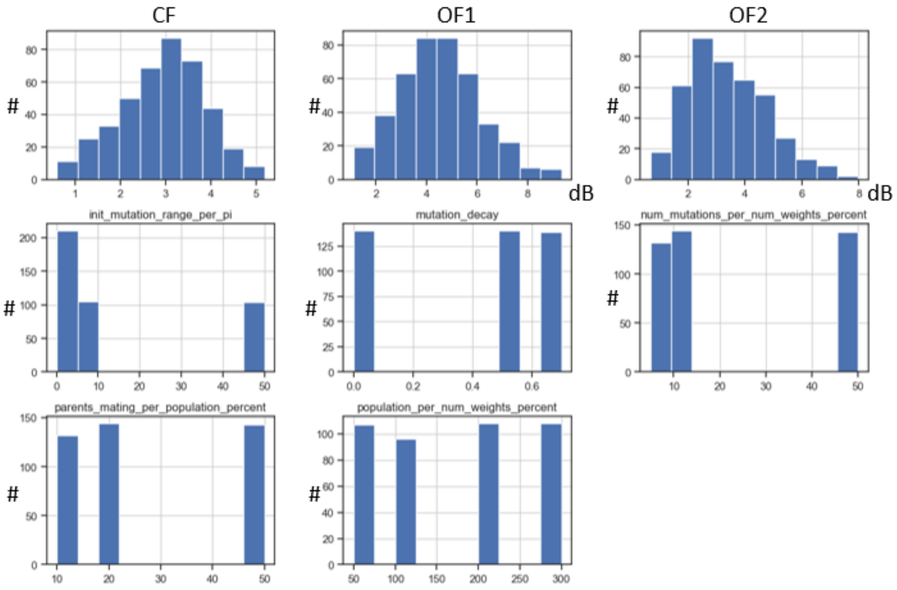

Supplementary Figure 8 | Optical beamsplitter 1x8 grid search results with genetic algorithm for CF^{1x8}_2: Histogram including all datasheets.

In this case, a 7.39 % of the samples achieve the targeted metrics for both output features simultaneously. Although results are better than the ones obtained from CF^{1x8}_1, they reflect again both the dependence of the hyperparameter values with the completion of the task and that the hyperparameter ranges selected are too broad. Again, we limited the number of operations to 3000. With this example, it can be appreciated that the selection of an optimum cost function is essential to optimize the self-configuration performance and to relax the impact of the selected hyperparameters.

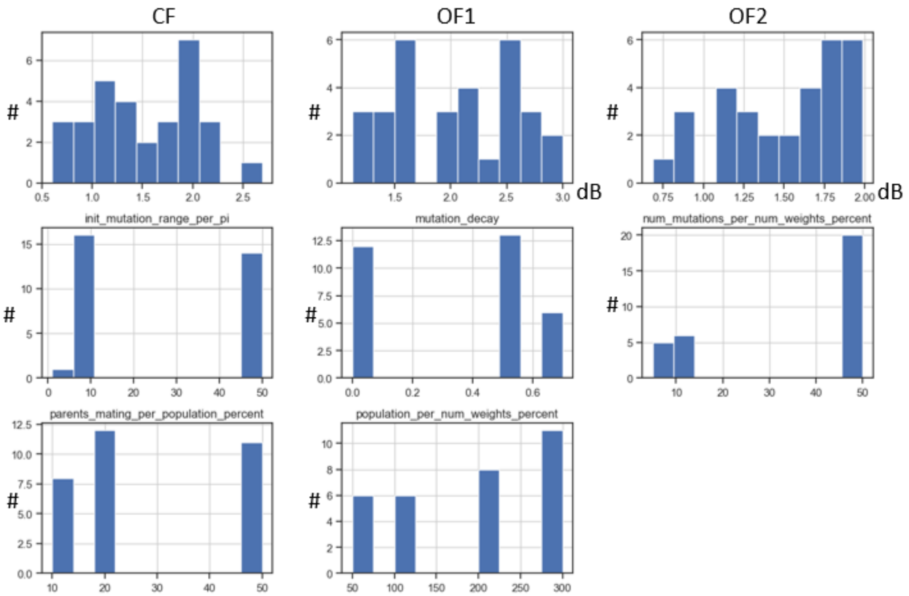

Supplementary Figure 9 | Optical beamsplitter 1x8 grid search results with genetic algorithm for CF^{1x8}_2: Histogram including the best performance datasheets.

Now, to show the statistical success rate of the algorithm, we launch 100 trials using the same hyperparameters (as specified in Supplementary Table I) and change the initial offsets of the TBUs for each trial.

Supplementary Table I | Selection of the best hyperparameter combinations after the grid-search for the application of a genetic algorithm in the self-configuration of a 1x8 optical beamsplitter to be used in the statistical test

**(CF^{1x8}_2)**	**Best performance ranges**
mutation_decay	0.5
parents_mating_per_num_weights_percent	25%
population_per_num_weights_percent	250%
num_mutations_per_num_weights_percent	50%
init_mutation_range_per_pi	50%

The statistical results are plotted in Supplementary Figure 10. Here, we can infer from the trend that in all cases, a larger number of operations would improve the results, both reducing the optical loss of the channel and their ripples. With 3000 operations, the 25% of the data have OF1 better than 3 dB and ripples better than 3 dB. In addition, whereas 78% of the data achieves the target proposed for the OF1, the average ripples in the channel requires more tuning. Solutions to improve the efficiency of the algorithm ranges from increasing the number of operations to performing cost function engineering considering a higher weight for the ripples. Nevertheless, 100% of the solutions present a result very close to the global optimum. At this point, once closed to the global minimum, a third approach consist of launching a second iteration with an optimization routine with exploitation capabilities like a derivative based approach or the simplex method.

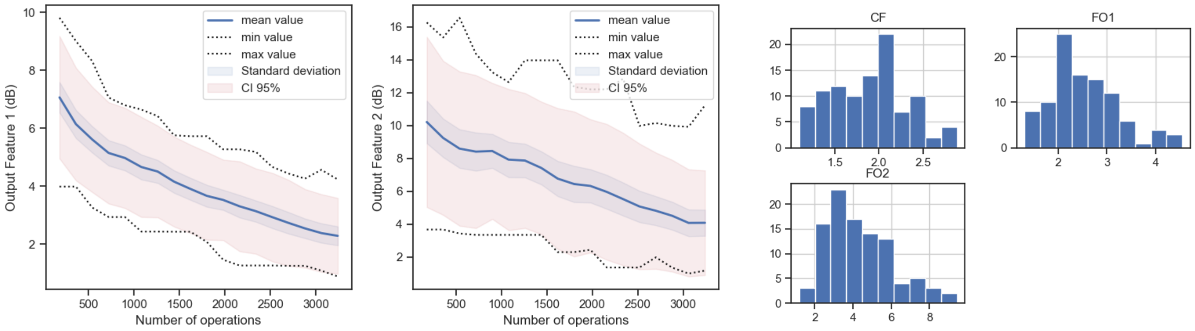

Supplementary Figure 10 | Optical beamsplitter 1x8 grid statistical results for fixed hyperparameter selection with genetic algorithm for CF^{1x8}_2: Evolution of the output features (OF1: mean of normalized output channels power of the beamsplitters, OF2: Mean ripple at the output channels). Progress (left) and histogram at last iteration (right) The datasheet is composed of 100 independent experiments with different waveguide mesh initial conditions.

Conclusions on the GA in the 1x8 beamsplitter example

We have applied different combinations of hyperparameters to two cost functions describing the operation of a 1x8 optical beamsplitter. We show that the success rate is dependent on the hyperparameter and the cost function selection. However, the best behaved

hyperparameters ranges are maintained between cost functions. In addition, we repeated 100 times a configuration test, varying the initial conditions (loss distribution and phase offsets) in the waveguide mesh arrangement showing a success rate of 25% for a given output feature metrics targeting average deviations better than 3 dB and average ripples in the channel better than 3-dB, always employing less than 3000 operations.

The efficiency and final performance achieved by the self-configuring algorithm could be improved by using different decrement schedules for the decay function or employing a cost function that includes the non-targeted optical ports leaked power. Also, the GA employed is a simple version of the method. Different selection methods and elitism strategies can be programed to achieve faster convergence to the global optimum. Moreover, although the optimum performance is not achieved in some cases, the trends shows that a larger number of operations would improve the current result. Finally, the use of the algorithm result in a configuration vector ***v*** closer to the global optimum of the function, and an algorithm with exploitation abilities can be sequentially employed with the resulting position as an starting point, as we will see for the gradient descent for the simplex method [11].

- **Optical beamsplitter 1x8 channels self-configuration employing a Particle Swarm Optimization (PSO) algorithm:**

The efficiency of the PSO algorithm is dependent on the hyperparameters selected, the cost function and the targeted problem. Like the previous case, we perform a grid-search tuning the hyperparameters and running the algorithm for 320 trials for each of the two proposed cost functions. The hyperparameters are selected following the ranges specified in Supplementary Table . We selected wide ranges to ensure that we explore different combinations.

CF^{1x8}_1:

Once computed the full datasheet, we compute the correlation matrix. It includes the final cost function, the output features and the values of the hyperparameters and returns a coefficient describing the linear correlation of the data as a good first-search indicator. Precisely, Supplementary Table suggests that in order to minimize the cost function and the output feature 1 and 2, one should employ a moderately large *inertia* coefficient, a moderate *cognitive* coefficient, and *number of particles*. Finally, the *social* value should be moderately large, within the range tested.

Supplementary Table 8 | Correlation matrix after the reconfiguration example

**(CF^{1x8}_1)**	**CF**	**OF1**	**OF2**
**CF**	1.000000	0.953957	0.464912
**OF1**	0.953957	1.000000	0.545596
**OF2**	0.464912	0.545596	1.000000
inertia	0.783003	0.726994	0.243692
cognitive	-0.043652	-0.040700	0.037724
particles_per_num_weights_percent	-0.148211	-0.165540	-0.167069
social	-0.161279	-0.122487	-0.090509

To delve into the actual performance of the different combinations, Supplementary Figure 11 and Supplementary Figure 12, illustrate the histogram of the complete datasheet and the datasheets featuring the targeted performance, respectively. The second datasheet includes thus the best results accomplishing OF_1 better than 3 dB and OF_2 better than 3 dB. From the data, we infer that the *init_mutation_range_per_pi* value should be close to 50%. A lower value limits the exploration capability of the algorithm in favor to the exploitation capability, increasing the probability to get stacked in a local minimum. In addition, for larger values of *init_mutation_range_per_pi*, the best *mutation_decay* is 0.5. The value of *num_mutations_per_num_weights_percent* should be close to 50%. The *parents_mating_per_num_weights_percent* should be close to 10 and 20 %.

From the data we obtain that the best ranges are:

Supplementary Table 9 | Selection of the best hyperparameter ranges

**(CF^{1x8}_1)**	**Best performance ranges**
Number of particles per weight %	20-100%
Inertia coefficient	0.5
Cognitive coefficient	0.5-1
Social coefficient	1-2

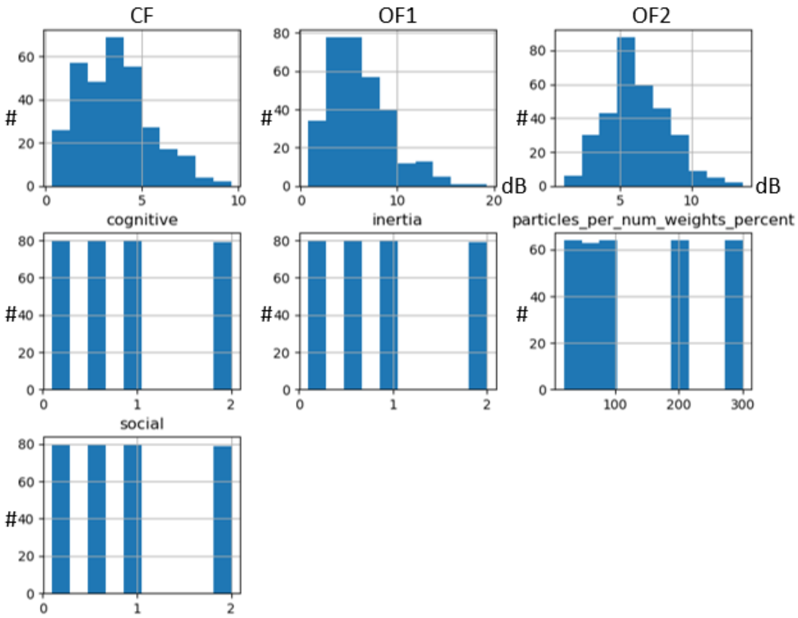

Supplementary Figure 11 | Optical beamsplitter 1x8 grid search results with genetic algorithm for CF^{1x8}_1 : Histogram including all datasheets.

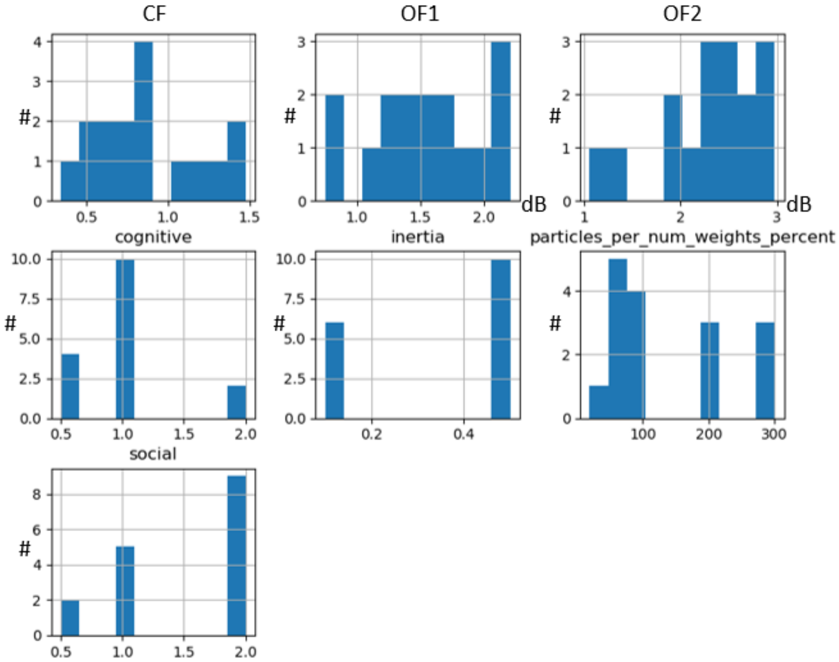

Supplementary Figure 12 | Optical beamsplitter 1x8 grid search results with genetic algorithm for CF^{1x8}_1: Histogram including the best performance datasheets.

In this case, it results that the 5.0% of the samples have succeed in the self-configuration task. This reflects, again, both the dependence of the hyperparameter values with the completion of the task and that the hyperparameter ranges selected are too broad. We limited the number of operations to 3000 for every trial, so slower convergency samples might be considered as failed. It is worth nothing that each operation implies a single configuration of the waveguide mesh and the extraction of the amplitude scattering matrix at the 8 output channels.

***CF^{1x8}_2*:**

Once computed the full datasheet of every trial, we compute the correlation matrix. It includes the final cost function, the output features and the values of the hyperparameters. As a result, it returns a coefficient describing the linear correlation of the data as a good first-search indicator. Precisely, Supplementary Table suggests that in order to minimize the cost function and the output feature 1 and 2, one should employ a moderately high *inertia* coefficient, and a moderate *number of particles*. Finally, the *social* value should be moderately small, within the range tested.

Supplementary Table 10 | Correlation matrix after the application of the PSO algorithm for the case CF^{1x8}_2

(CF^{1x8}_2)	CF	OF1	OF2
**CF**	1.000000	0.960125	0.359904
**OF1**	0.960125	1.000000	0.397768
**OF2**	0.359904	0.397768	1.000000
inertia	0.773356	0.716420	0.184801
cognitive	-0.011251	-0.032298	-0.025226
particles_per_num_weights_percent	-0.175712	-0.208913	0.054592
social	-0.126041	-0.093567	-0.100469

To delve into the actual performance of the different combinations, Supplementary Figure 13 and Supplementary Figure 14, illustrate the histogram of the complete datasheet and the datasheets featuring the targeted performance, respectively. The second datasheet includes thus the best results accomplishing OF_1 better than 3 dB and OF_2 better than 2 dB. From the data, we infer that the *init_mutation_range_per_pi* value should be close to 50%. A lower value limits the exploration capability of the algorithm in favor to the exploitation capability, increasing the probability to get stacked in a local minimum. In addition, for larger values of *init_mutation_range_per_pi*, the best *mutation_decay* is 0.5. The value of *num_mutations_per_num_weights_percent should be close to 50%. The parents_mating_per_num_weights_percent* should be close to 10 and 20 %.
From the data we obtain that the best ranges are:

Supplementary Table 11 | Selection of the best hyperparameter values

(CF^{1x8}_2)	Best performance ranges
Number of particles per weight %	50-200%
Inertia coefficient	0.5
Cognitive coefficient	0.5-1
Social coefficient	1-2

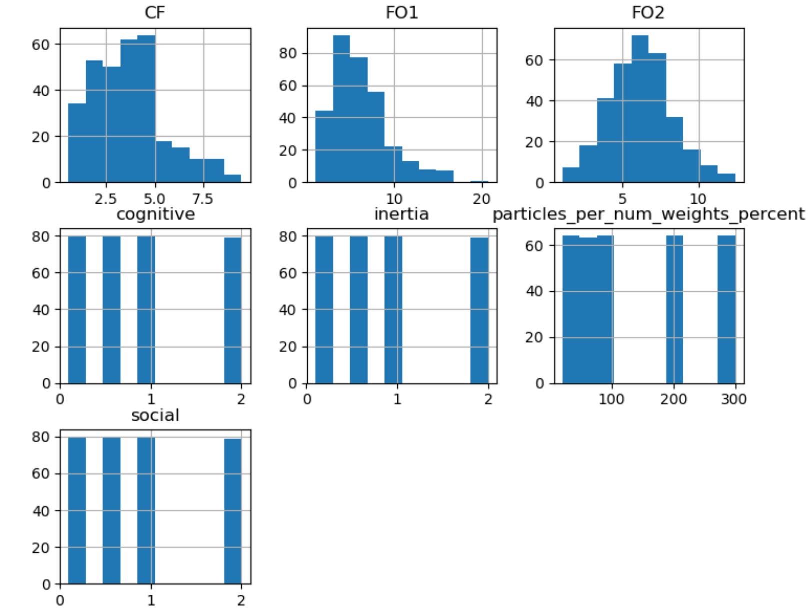

Supplementary Figure 13 | Optical beamsplitter 1x8 grid search results with genetic algorithm for CF^{1x8}_2: Histogram including all datasheets.

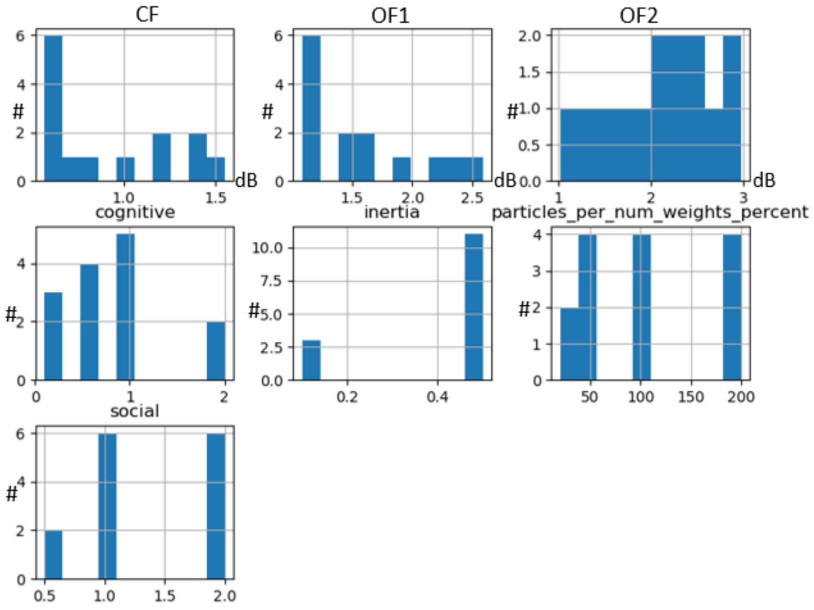

Supplementary Figure 14 | Optical beamsplitter 1x8 grid search results with genetic algorithm for CF^{1x8}_{2}: Histogram including the best performance datasheets.

In this case, the preliminary analysis shows that 4.3 % of the samples have succeed in the self-configuration task, reflecting again both the dependence of the hyperparameter values with the completion of the task and that the hyperparameter ranges selected are too broad. We limited the number of operations to 3000 for every trial. It is worth nothing that each operation implies a single configuration of the waveguide mesh and the extraction of the amplitude scattering matrix at the 8 output channels.

Now, to show the statistical success rate of the algorithm, we will launch 100 trials using the same hyperparameters and changing the initial offsets of the TBUs at each experimental trial.

Supplementary Table II | Selection of best hyperparameters employed in the statistical analysis.

**(CF^{1x8}_{2})**	**Best performance ranges**
Number of particles per weight %	200%
Inertia coefficient	0.5
Cognitive coefficient	0.5
Social coefficient	1.0

From this statistical analysis we can see that 89% of the trials have obtained an OF1 better than 3dB. Maintaining this performance, a 31% of the trials achieve an OF2 better than 3dB. The trends suggest that a greater number of operations would improve the statistical result. However, we see that the improvement rate is reduced for OF2, suggesting that some of the samples might be closed to a local minimum.

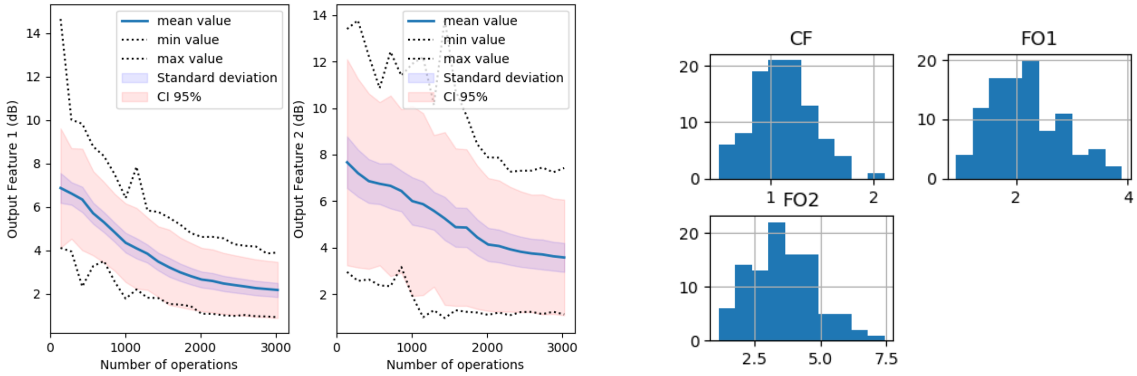

Supplementary Figure 15 | Optical beamsplitter 1x8 grid statistical results for fixed hyperparameter selection with PSO algorithm for CF^{1x8}_{2}: Evolution of the output features (OF1: mean of normalized output channels power of the beamsplitters, OF2: Mean ripple at the output channels). Progress (left) and histogram at last iteration (right) The datasheet is composed of 100 independent experiments with different waveguide mesh initial conditions.

Conclusions on the PSO in the 1x8 example

We applied different combinations of hyperparameters to two cost functions describing the operation of a 1x8 optical beamsplitter. We show that the "success rate" is dependent on the hyperparameter selection. However, the best behaved hyperparameters ranges are maintained between both cost functions. In addition, we repeated 100 times a configuration test, varying the initial conditions (loss distributions and phase offsets) in the waveguide mesh arrangement showing a success rate of 32% always employing less than 3000 operations.

The efficiency of the algorithm could be improved by using advanced versions of the plain PSO. Moreover, although the optimum performance is not achieved in some cases, the use of the algorithm result in a position much closer to the global optimum of the function, and an algorithm with exploitation abilities can be sequentially employed with the resulting position as an starting point, as we will see for the gradient descent and to the simplex method [11]. Compared to the genetic algorithm applied to the same application example, the PSO algorithm achieves a 7% more of success rate for the selected best configuration. A better convergence speed and less sensitivity to the hyperparameters can be achieved if parameters like the inertia are configured adaptatively to decrease during the optimization process.

- **Optical beamsplitter 1x8 channels self-configuration employing a gradient-descent with momentum algorithm:**

The gradient descent algorithm is the simplest approach of optimization methods that require the computation of the gradient (the derivative of the cost functions for each variable in the search-space). Here we illustrate its application to the two cost functions engineered for the self-configuration of the optical beamsplitter 1x8. This algorithm, as described in Supplementary Material 5 requires the selection of two hyperparameters. In order to illustrate how their selection affects to the minimization process we perform a grid search to find the best values for the learning rate (η) and the momentum (α_{mo}). We divided the grid as: $\eta \in$ [5e-2 1e-1 5e-1 1] and $\alpha_{mo} \in$ [0 0.2 0.4 0.6 0.8 0.9].

From Supplementary Figure 16, we can see that the cost function final values, the average optical power at the selected output channels displayed at Output Feature 1, and their average ripple in dBs displayed at Main Feature 2, are acceptable (<1, around -1dB, <1.5 dB, respectively) for most of the points where α_{mo} is greater than 0.4. However, the optimum number of iterations to convergence is achieved for $\alpha_{mo} \geq 0.8$ and η around 1e-1, resulting in a number of 43-50 iterations, 0.4-0.8-dB average ripples, and -10.2 to -10.4 dBm average output power. Per iteration, the algorithm performs 72 operations, ranging between 6192 and 7200 operations for the best cases. These numbers can potentially by decreased by half if partial non-central derivatives are employed.

***$CF^{1x8}{}_2$*:**

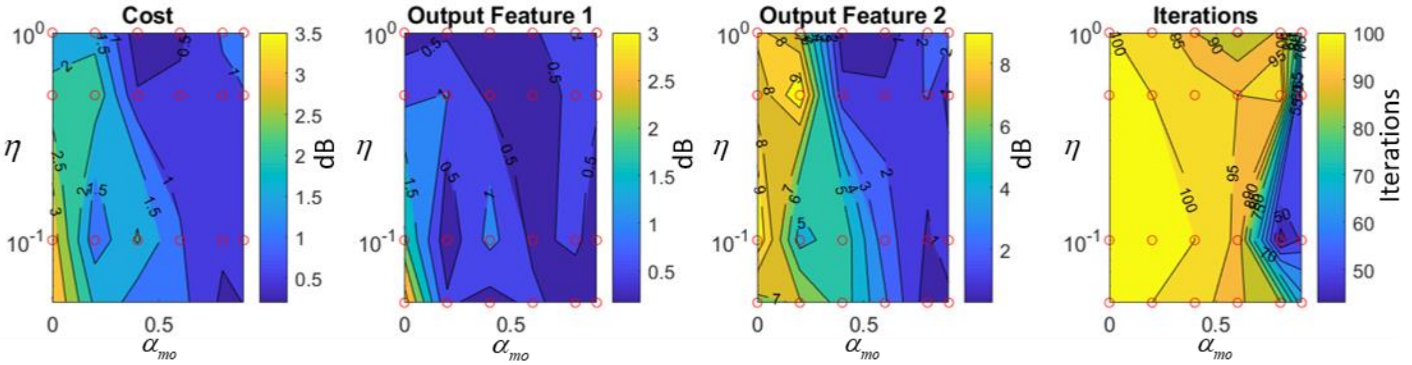

Supplementary Figure 16 | Optical beamsplitter 1x8 grid search results with gradient descent with momentum for $CF^{1x8}{}_2$: Cost, Output Feature considering the average at the targeted outputs in dBm, Output Feature 2 considering the ripple in the channel in dB, and the number of iterations to converge. A maximum number of 100 iterations is allowed and then associated to not convergence. The red circles show the computed points.

Considering the aforementioned example at α_{mo} = 0.8 and η = 2e-1. The α_{mo} is progressively raised each iteration by an amount of 0.05 until reaching the targeted value to enhance the convergence and avoid noisy starts.

Supplementary Note 6: Self-configuration of *all-cross* waveguide meshes.

All-cross function aims to configure every phase actuator to set every TBU to their cross-state. This function is particularly interesting for both *calibration and characterization* as well as for setting an optimized initial point for some of the optimization methods described in this work and enhance their convergence.

The cost function is just an example of a broad range of applications involving arbitrary routing of the optical signals between the optical ports. This particular case is associated to the minimization process of a mathematical function that involves the readouts corresponding to the optical channels that are configured when all the TBUs in the arrangement are in *cross-state*. For example, in Supplementary Figure 17, the optical channels are described by the following port pairs: 12-23, 14-21, 10-1, 8-3, 13-6, 15-4, 17-2, 19-24, and 11-16, 9-18,7-20, 5-22. Note that the last set of 4 port pairs is redundant and could be deleted from the cost function as they do not incorporate additional TBUs. In addition, one could incorporate extra features to the cost function to enhance the convergence of the process or reduce the number of iterations, however it comes at the cost of additional number of measurements or signal processing. Few examples are the monitoring of the spectral response of those channels to incorporate the observed ripples to the cost function or the monitoring of a set of undesired paths. Although not incorporated in the following *CF* definition, they should be contemplated as a solution for the future scalability of large-scale waveguide mesh arrangements. Finally, the selection of the cost function is essential to enhance and, in some cases, enable the convergence of the optimization process.

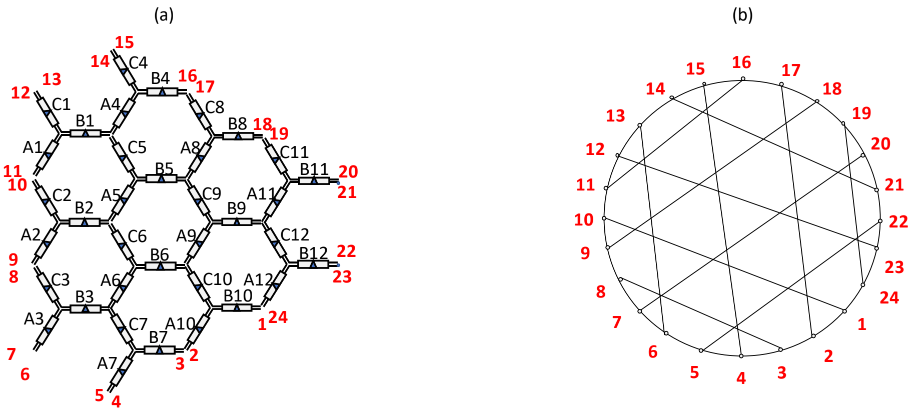

Supplementary Figure 17 | (a) Labelled schematic of the waveguide mesh arrangement under test. (b) black box system with the targeted performance: (a) Labelled schematic of the waveguide mesh arrangement under test. (b) black box system with the targeted performance: Routing between channels defined by the port pairs 12-23, 14-21, 10-1, 8-3, 13-6, 15-4, 17-2, 19-24, and 11-16, 9-18,7-20, 5-22. Note: They represent direct connections without crossings or splitting.

The cost function is described as follows:

$$CF_1^{allcross} = \begin{cases} c_1 = -1, \\ f_1 = \dfrac{1}{N}\sum_{1}^{chs.}\left(\log\left(\left|H_{chs.}\right|\right)\right), \end{cases} \qquad (9)$$

where N is the number of optical channels incorporated in the optimization process and $|H_{chs}|$ the maximum absolute value of the electric field measured for each optical channel listed before.

To perform an intuitive analysis of the achieved performance, we monitor and define the Output Feature 1 as the average power transmission response in the targeted optical channels defining the *all-cross* operation. It is expressed in logarithmic units.

Data analysis for the different advanced optimization methods:

- ***All-cross*** **self-configuration employing a Genetic Algorithm**

The efficiency of the genetic algorithm is dependent on the hyperparameters selected, the cost function and the targeted problem. In order to test the performance and to find the best ranges for the hyperparameters for the *all-cross* configuration problem, we perform a grid-search tuning the hyperparameters and running the algorithm for 409 trials the $CF^{\text{all-cross}}_1$**.** The hyperparameters are selected following the ranges specified in Supplementary Table 1. We selected wide ranges to ensure that we explore different combinations of hyperparameters.

$\boldsymbol{CF^{\text{all-cross}}_1}$**:**

We repeat the correlation analysis between the cost function and the output feature and the hyperparameters. It computes the correlation matrix of the datasheet including the data gathered during the self-configuration process. In this case, results gathered in Supplementary Table suggest that in order to minimize the cost function and maximize the *Output Feature* 1, one should employ a moderately large

init mutation range per pi value, *num_mutations_per_num_weights_percent* and *population_per_num_weights_percent* value. Finally, the *mutation_decay* value should be moderately small, within the range tested and is proportional to the *init_mutation_range_per_pi* value..

Supplementary Table 13 | Correlation matrix after the reconfiguration of $CF^{all\text{-}cross}{}_1$

**(*$CF^{all\text{-}cross}{}_1$*)**	**CF**	**OF1**
**CF**	1.000000	-0.973491
**OF1**	-0.973491	1.000000
mutation_decay	0.083603	-0.103660
parents_mating_per_population_percent	-0.038304	0.017483
mutationpermating	-0.214166	0.207822
parents_mating_per_num_weights_percent	-0.156005	0.146814
population_per_num_weights_percent	-0.210828	-0.260325
num_mutations_per_num_weights_percent	-0.386104	0.374420
mutation_decay vs range	-0.365893	0.342917
init_mutation_range_per_pi	-0.428195	0.406466

To delve into the actual performance of the different combinations, Supplementary Figure 18 and Supplementary Figure 19, illustrate the histogram of the complete datasheet and the datasheets featuring an output feature value better than 3-dB, respectively. From the data, we infer that the *init_mutation_range_per_pi* value should be close to 50%. A lower value limits the exploration capability of the algorithm in favor to the exploitation capability, increasing the probability to get stacked in a local minimum. In addition, for larger values of *init_mutation_range_per_pi*, the best *mutation_decay* is 0.5. The value of *num_mutations_per_num_weights_percent should be close to 50%. The parents_mating_per_num_weights_percent* should be close to 10 and 20 %.

Supplementary Table 14 | Selection of the best hyperparameter ranges.

**(*$CF^{all\text{-}cross}{}_1$*)**	**Best performance ranges**
mutation_decay	0.2-0.5
parents_mating_per_num_weights_percent	25%
population_per_num_weights_percent	50-200%
num_mutations_per_num_weights_percent	50%
init_mutation_range_per_pi	20-50%

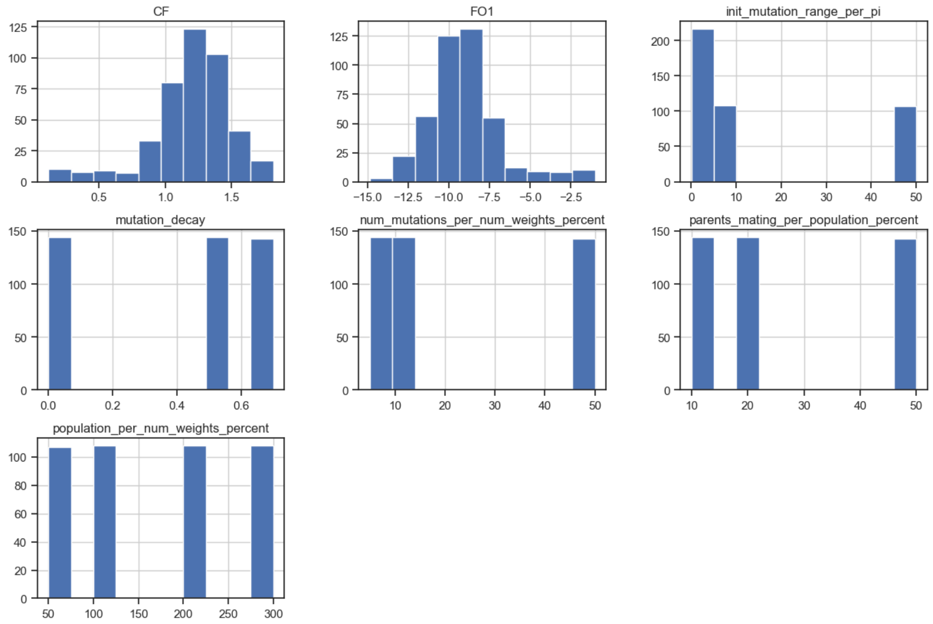

Supplementary Figure 18 | All-cross routing function grid search results with genetic algorithm for $CF^{all\text{-}cross}{}_1$: Histogram including all datasheets.

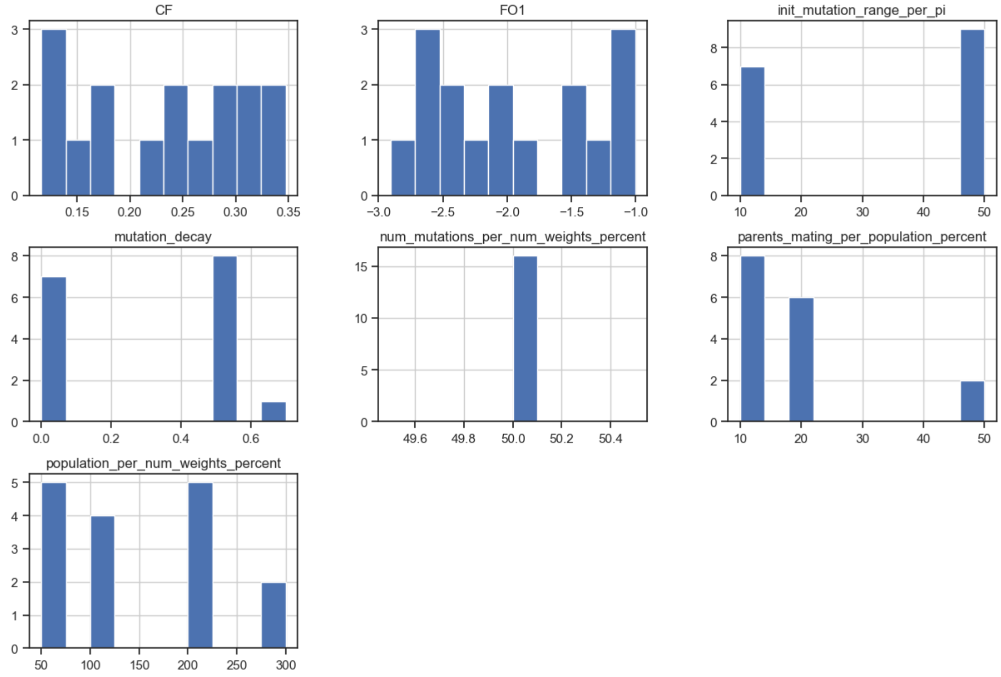

Supplementary Figure 19 | All-cross routing function grid search results with genetic algorithm for $CF^{all\text{-}cross}_1$: Histogram including the best performance datasheets.

For these tests, the 3.7 % of the samples have succeed in the self-configuration task. This reflects both the dependence of the hyperparameter values with the completion of the task and that the hyperparameter ranges selected are too broad. We limited the number of operations to 3000 for every trial, so slower convergency samples might be considered as failed. It is worth nothing that each operation implies a single configuration of the waveguide mesh and the extraction of the amplitude scattering matrix at the 12 optical channels. In order to test the statistical variability of the method we run 100 experiments with different initial conditions as phase offsets and loss distributions of the waveguide mesh arrangement, employing the hyperparameters of Supplementary Table .

Supplementary Table 15 | Selection of the best hyperparameter ranges to be employed in the statistical analysis.

**($CF^{all\text{-}cross}_1$)**	**Best performance ranges**
mutation_decay	0.5
parents_mating_per_num_weights_percent	25%
population_per_num_weights_percent	200%
num_mutations_per_num_weights_percent	50%
init_mutation_range_per_pi	50%

As illustrated in Supplementary Figure 20 the 95% of the samples show an Output Feature better than 3 dB. In addition, both the percentage and the mean would improve if we increase slightly the number of operations, as suggested by the trend.

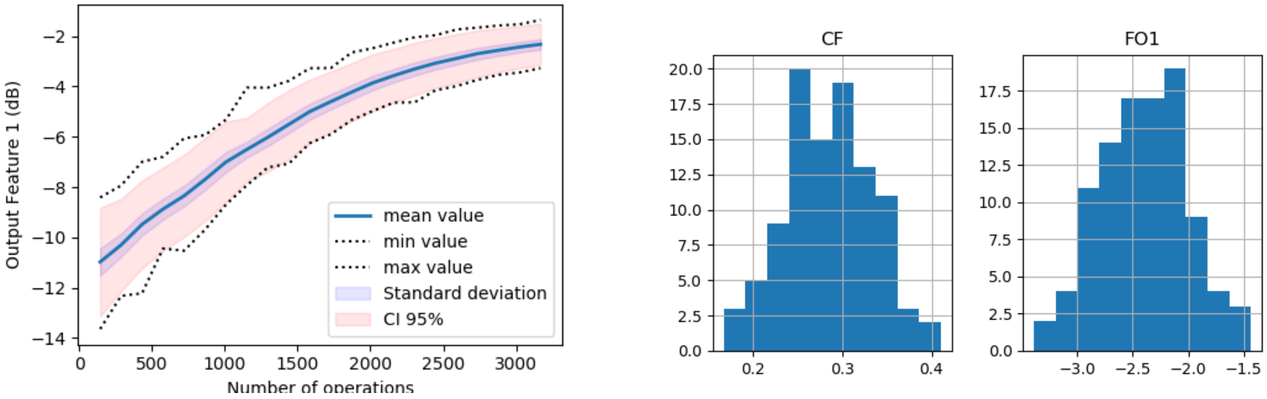

Supplementary Figure 20 | All-cross function statistical results for fixed hyperparameter selection with genetic algorithm for $CF^{all\text{-}cross}_1$: Evolution of the output features (OF1: mean of normalized output channels power of the beamsplitters, OF2: Mean ripple at the output channels). Progress (left) and histogram at last iteration (right) The datasheet is composed of 100 independent experiments with different waveguide mesh initial conditions.

Conclusions on the GA in the all cross self-configuration example

We applied different combinations of hyperparameters to one cost functions describing the operation of a routing function requiring all the programmable units being in cross state. We show that the success rate is dependent on the hyperparameter selection. In addition, we repeated 100 times a configuration test, varying the initial conditions or phase offsets in the waveguide mesh arrangement showing that 95% achieves an average error in their optical channels of less than 3-dB, always employing less than 3000 operations.

- ***All-cross* self-configuration employing a Particle Swarm Optimization (PSO) algorithm:**

The efficiency of the PSO algorithm is dependent on the hyperparameters selected, the cost function and the targeted problem. Like the previous case, we perform a grid-search tuning the hyperparameters and running the algorithm for 320 trials employing the cost function. The hyperparameters are selected following the ranges specified in Supplementary Table 1. We selected wide ranges to ensure that we explore different combinations.

$CF^{All\text{-}cross}_1$:

Once computed the full datasheet of every trial, we compute the correlation matrix. It includes the final cost function, the output features and the values of the hyperparameters. As a result, it returns a coefficient describing the linear correlation of the data as a good first-search indicator. Precisely, Supplementary Table suggests that in order to minimize the cost function and the output feature, one should employ a moderate *inertia* coefficient, a moderately high *number of particles*. Finally, the *social* value should be moderately high, within the range tested.

Supplementary Table 16 | Correlation matrix after the reconfiguration of $CF^{All\text{-}cross}_1$

($CF^{All\text{-}cross}_1$)	CF and OF1	OF2
**CF**	1.000000	-0.985750
**OF1**	1.000000	-0.985750
**OF2**	-0.985750	1.000000
inertia	0.490296	-0.474098
cognitive	0.006863	0.006605
particles_per_num_weights_percent	-0.113016	0.113350
social	-0.269188	0.280533

To delve into the actual performance of the different combinations, Supplementary Figure 21 illustrates the histogram of the complete datasheet and the datasheets featuring the targeted performance, respectively. The second datasheet includes thus the best results accomplishing OF_1 better than 3 dB. From the data, we infer that hyperparameters obtaining the best results are the following:

Supplementary Table 17 | Selection of the best hyperparameter ranges

($CF^{All\text{-}cross}_1$)	Best performance ranges
Number of particles per weight %	100%
Inertia coefficient	0.5
Cognitive coefficient	1.0
Social coefficient	1.0-2.0

From the trials, it results that 7.28% of the samples have succeed in the self-configuration task. This reflects both the dependence of the hyperparameter values with the completion of the task and that the hyperparameter ranges selected are too broad. We limited the number of operations to 3000 for every trial, so slower convergency samples are considered as failed. It is worth nothing that each operation implies a single configuration of the waveguide mesh and the extraction of the amplitude scattering matrix at the 12 output channels. Compared to the genetic algorithm the PSO achieves the double efficiency. However, the comparison should not be understood as straightforward since the hyperparameter spaces in both cases is different.

In order to test statistically the success rate for a fixed set of best-performing set of hyperparameters, we test the method 100 times with variable initial conditions for the waveguide mesh arrangement. We employ the following combination expressed in Supplementary Table 18.

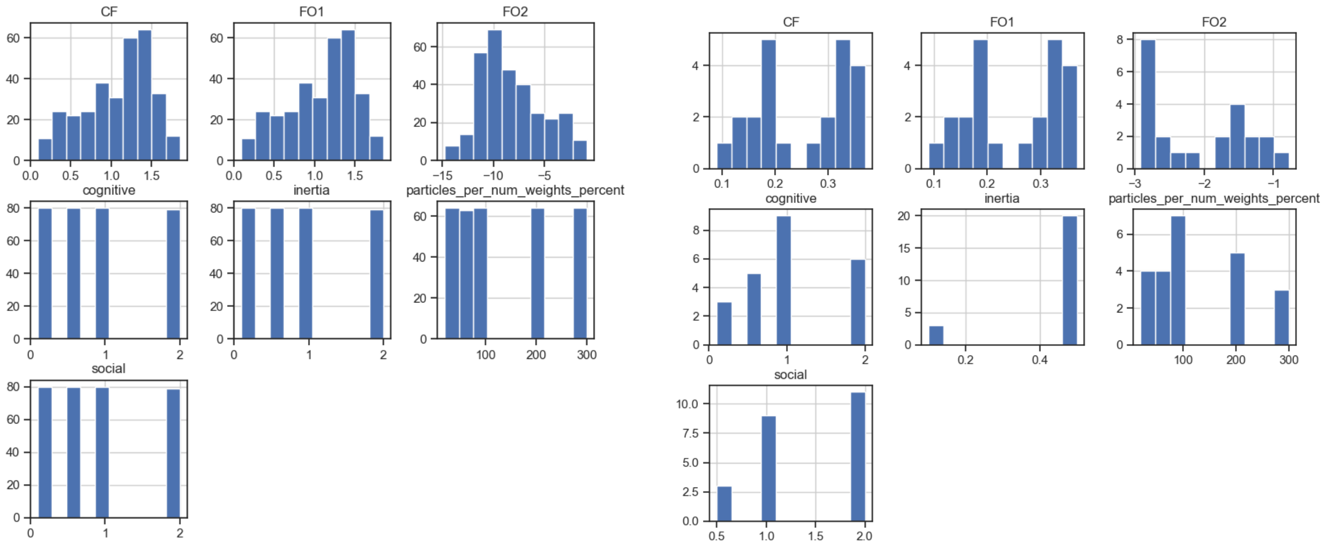

Supplementary Figure 21 | All cross grid search results with genetic algorithm for $CF^{all\text{-}cross}_1$: Histogram including all datasheets.(left) and the best cases (right)

Supplementary Table 18 Selection of the best hyperparameter ranges employed in the statistical analysis.

**($CF^{allcross}_1$)**	**Selected values**
Number of particles per weight %	150%
Inertia coefficient	0.5
Cognitive coefficient	0.8
Social coefficient	1.0

As illustrated in Supplementary Figure 22 the 70% of the samples show an Output Feature better than 3 dB. In addition, both the percentage and the mean would improve if we increase slightly the number of operations, as suggested by the trend.

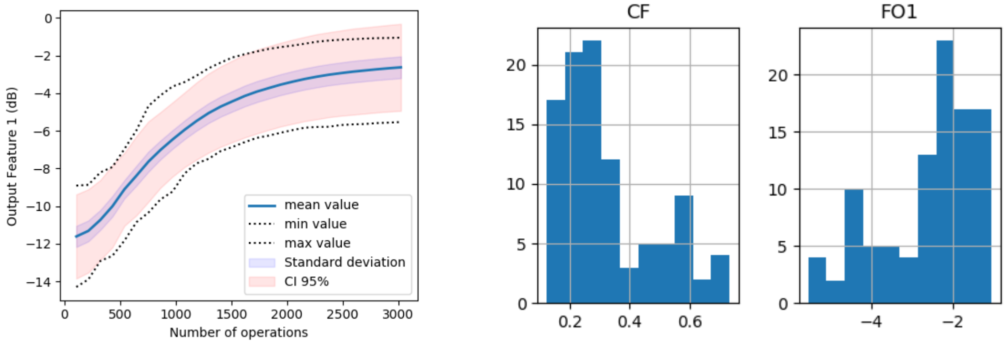

Supplementary Figure 22 | All-cross function statistical results for fixed hyperparameter selection with PSO algorithm for $CF^{all\text{-}cross}_1$: Evolution of the output features (OF1: mean of normalized output channels power of the output channels, OF2: Mean ripple at the output channels). Progress (left) and histogram at last iteration (right) The datasheet is composed of 100 independent experiments with different waveguide mesh initial conditions.

Conclusions on the PSO in the all cross self-configuration example

We applied different combinations of hyperparameters to one cost function describing the operation of a routing function requiring all the programmable units being in cross state. We show that the success rate is dependent on the hyperparameter selection. In addition, we repeated 100 times a configuration test, varying the initial conditions or phase offsets in the waveguide mesh arrangement showing a success rate of 70% always employing less than 3000 operations. For this particular case, and hyperparameter combination the genetic algorithm outperforms the PSO. In both cases the configuration of the device is very close to the ideal value and either employing more iterations or improving the cost functions to consider spectral feature, and leaking power would improve the convergence rates. In addition, this can be employed as the starting point of a gradient-descent based optimization procedure or a Nelder-Mead method that have easier exploitation capabilities of the nearest minimum point. A better convergence speed and less sensitivity to the hyperparameters can be achieved if parameters like the inertia are configured adaptatively to decrease during the optimization process.

- ***All-cross* self-configuration employing a gradient-descent with momentum algorithm:**

The efficiency of the gradient descent with momentum algorithm is dependent on the two hyperparameters selected, the cost function and the targeted problem. Like in the previous case, we perform a sweep over a wide range for each hyperparameter to illustrate how their selection affects to the minimization process. Thus, we perform a grid search to find the best values for the learning rate (η) and the momentum (α_{mo}). We divided the grid as: $\eta \in$ [1 1e1 5e1 1e0] and $\alpha_{mo} \in$ [0 0.2 0.4 0.6 0.8 0.9].

$CF^{all\text{-}cross}_{1}$:

The resulting sweep is plotted in Supplementary Figure 23. Here we can see that the final cost function value and the average optical power at the selected output channels are acceptable (<0.2 and -1dB, respectively) for most of the points where α_{mo} is greater than 0.4. However, the optimum number of iterations to convergence is achieved for $\alpha_{mo} \geq 0.5$ and $\eta \geq 50$, resulting in a number of 5-6 iterations. At each iteration we have computed the gradient of ***v*,** implying a total of 750 – 900 operations.

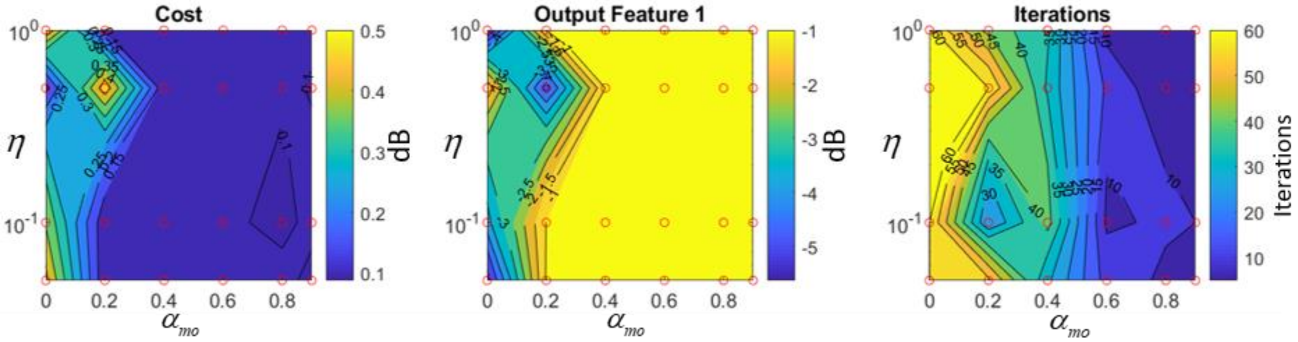

Supplementary Figure 23 | Example results of the grid-search of the optimization process of the CF (1) and the Gradient Descent algorithm. Cost, Main Feature considering the average at the targeted outputs in dBm and the number of iterations to converge. A maximum number of 60 iterations is allowed and then associated to not convergence. The red circles show the computed points.

As an illustrative example, we analyse in Supplementary Figure 24 the optimization process considering the aforementioned example and $\alpha_{mo} = 0.5$ and $\eta = 50$. The α_{mo} is progressively raised each iteration by an amount of 0.05 until reaching the targeted value to enhance the convergence and avoid noisy starts.

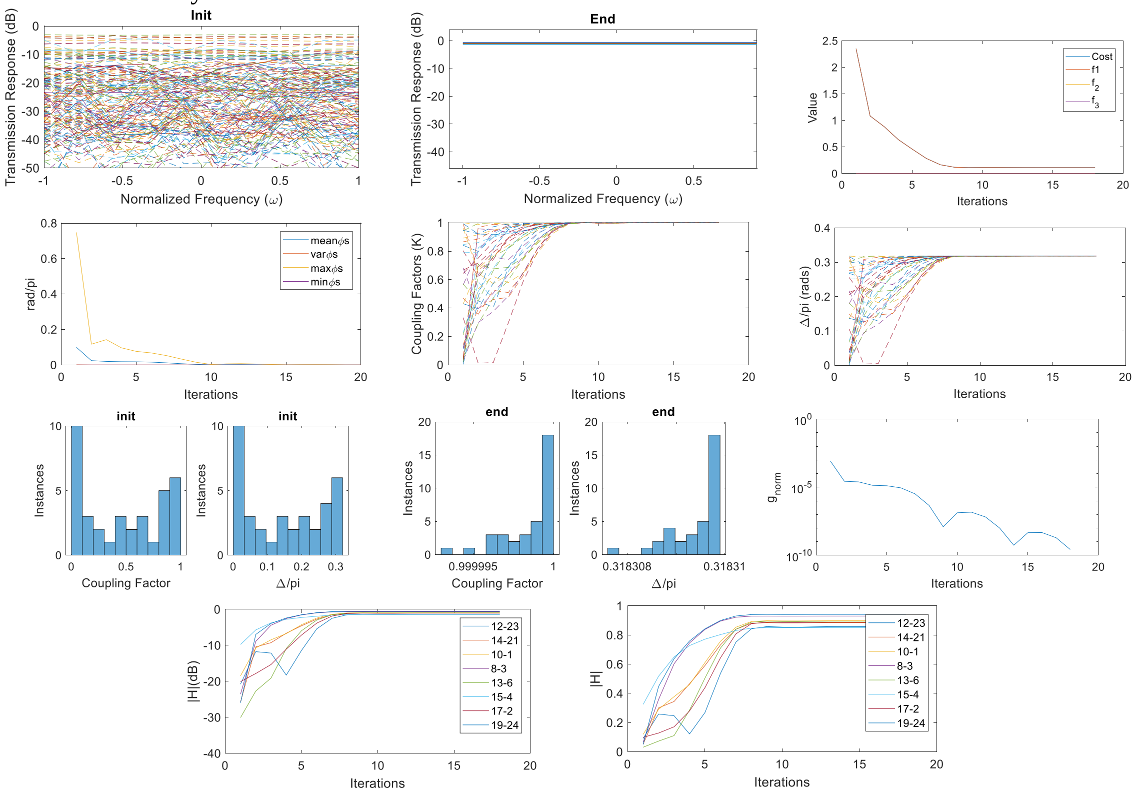

Supplementary Figure 24 | Resulting log-file of the grid-search of the optimization process of the CF (1) and the Gradient Descent algorithm with $\alpha_{mo} = 0.5$ and $\eta = 50$. Initial spectral response of the desired and undesired optical channels, final spectral response of the desired and undesired

optical channels, Cost Function value per iteration, statistical results of the driving phases per iteration, coupling factor per iteration (not employed in the optimization process), common phase of each TBU per iteration (not employed in the optimization process), and initial and final histograms of the coupling factor and common phase value of each TBU and norm of the gradient.. Complementary resulting log-file of the grid-search of the optimization process of the CF (1) and the Gradient Descent algorithm with $\alpha_{mo} = 0.5$ and $\eta = 50$ including the features related to the application. Absolute value of the scattering matrix elements related to the targeted optical channels and their evolution for each iteration, related optical power per iteration.

In the previous figure, we can see visual metrics that allows to understand the progress of the optimization process. First, Supplementary Figure 24(a-b) illustrates the initial and final spectral responses obtained from the scattering matrix. We can see that the initial state is a unitary random scattering matrix and the final response shows the flat response of the targeted all-cross channels. (c) illustrates the cost function and the features values during the optimization process. The first feature *f1* is proportional to the optical power at the targeted channels, whereas the second feature refers to the average ripple in the targeted channels.

Supplementary Note 7: Self-configuration of optical filters:

The design and configuration of optical filters as application specific photonic integrated circuits has been covered in multiple papers and books [13]. There, a circuit architecture is chosen and build up with optical splitters, combiners and waveguides. In this supplementary note, we demonstrate the self-configuration of optical filters in the general-purpose waveguide mesh arrangement. This application targets the suppression of a given spectral band while maintaining the minimum losses in the passband. The general-purpose waveguide mesh arrangement can be configured to perform as a filter relying on pre-characterization routines and pre-sets. However, having a self-configuration method is a promising capability to configure filters on demand. Also, letting an automated function select between thousands of parameters is a good solution for scalable systems and can potentially deal with the mitigation of non ideal effects like optical crosstalk, tuning crosstalk, power consumption savings, logical footprint savings, and optical loss improvements.
For the definition of the cost function, it is possible to consider many features, as the insertion loss of the pass band, the extinction ratio of the filter, the roll off, the optical power at the non-targeted ports, etc. They are illustrated in Supplementary Figure 25. Additionally, one can define the targeted spectral mask of the filter and define a cost function feature considering the error between the obtained trace and the mask at each iteration. Here we demonstrate the self-reconfiguration capability employing only the mean square error with the spectral mask, although multiple objectives could be added to the cost function to be optimized. The cost function employed is described by the following equation:

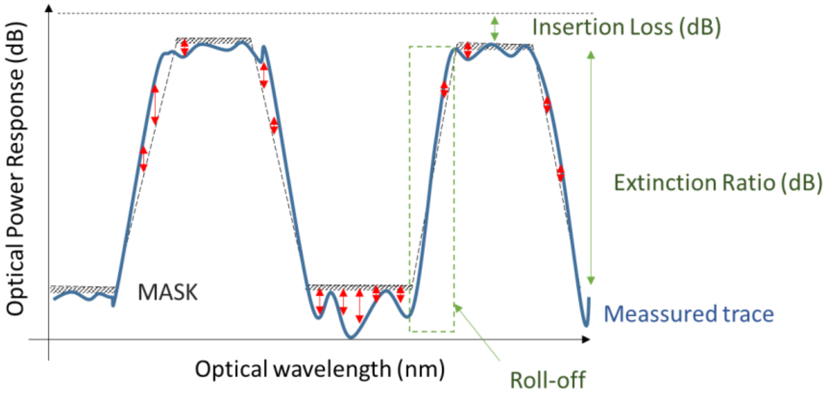

Supplementary Figure 25 | Optical filter performance scheme for the construction of the cost function $CF^{O.Filter}_1$.

$$CF_1^{O.Filter} = \begin{cases} c_1 = 1, \\ f_1 = \frac{1}{N_\lambda} \sum_{\lambda} \left(M_\lambda - 20\log_{10}\left(\left|S_{4,1}(\lambda)\right|\right)\right)^2, \end{cases} \quad \text{(S8)}$$

where N_λ is the number of wavelength points, M_λ is the value of the spectral mask at each wavelength point, and $S_{4,1}$ is the value of the scattering matrix at the optical channel defined by ports 4 and 1 at a given wavelength. Note that this operation is equivalent to the average of the distance between the mask and the measured trace depicted in red in the previous figure.
For the comparison between methods and future cost functions, we analyze also the evolution of two output features, dealing with the insertion loss in the passband (OF_1) and with the extinction ratio of the filter (OF_2).

Data analysis for the different advanced optimization methods:

- **Optical filter self-configuration employing a Genetic Algorithm**

The efficiency of the genetic algorithm is dependent on the hyperparameters selected, the cost function and the targeted problem. In order to test the performance and to find the best ranges for the hyperparameters for the *Optical Filter* self-configuration, we perform a grid-search tuning the hyperparameters and running the algorithm for 409 trials to minimize the $CF^{\text{Optical filter}}$**.** The hyperparameters are selected following the ranges specified in Supplementary Table 1. We selected wide ranges to ensure that we explore different combinations of hyperparameters.

$CF^{\text{Optical filter}}_1$:

We repeat the correlation matrix analysis of the datasheet including the data gathered during the self-configuration process. In this case, results shown in Supplementary Table suggest that in order to minimize the cost function, one should employ a *large init mutation range per pi* value, a *num_mutations_per_num_weights_percent* a moderate *population_per_num_weights_percent* value. Finally, the *mutation_decay* value should be moderately small, within the range tested.

Supplementary Table 19 | Correlation matrix after the reconfiguration employing $CF^{Optical\ filter}{}_{1}$

**($CF^{all\text{-}cross}{}_{1}$)**	**CF**	**OF1**	**OF2**
**CF**	1.000000	0.603358	-0.607061
**OF1**	0.603358	1.000000	-0.378884
**OF2**	-0.607061	-0.378884	1.000000
mutation_decay	-0.000787	0.023269	-0.113220
parents_mating_per_population_percent	-0.054180	-0.029678	0.150988
mutationpermating	-0.067246	-0.093016	0.042401
parents_mating_per_num_weights_percent	-0.132101	-0.051165	0.195070
population_per_num_weights_percent	-0.212540	-0.150821	0.188196
num_mutations_per_num_weights_percent	-0.203432	-0.242716	0.218610
mutation_decay vs range	-0.222523	-0.136275	0.097295
init_mutation_range_per_pi	-0.174769	-0.156635	0.137048

To delve into the actual performance of the different combinations, Supplementary Figure 26 and Supplementary Figure 27, illustrate the histogram of the complete datasheet and the datasheets featuring the targeted performance, respectively. The second datasheet includes thus the best results accomplishing OF_1 better than 5 dB and OF_2 better than 18 dB. From the data, we infer that the *init_mutation_range_per_pi* value should be, as in the previous applications of the GA algorithm, close to 50%. A lower value limits the exploration capability of the algorithm in favor to the exploitation capability, increasing the probability to get stacked in a local minimum. In addition, for larger values of *init_mutation_range_per_pi*, the best *mutation_decay* is 0.5. The value of *num_mutations_per_num_weights_percent* should be close to 50%. The *parents_mating_per_num_weights_percent* should be close to 10 and 20 %.

Supplementary Table 20 | Selection of best performing parameters

**($CF^{all\text{-}cross}{}_{1}$)**	**Best performance ranges**
mutation_decay	0.2-0.5
parents_mating_per_num_weights_percent	25%
population_per_num_weights_percent	50-200%
num_mutations_per_num_weights_percent	50%
init_mutation_range_per_pi	20-50%

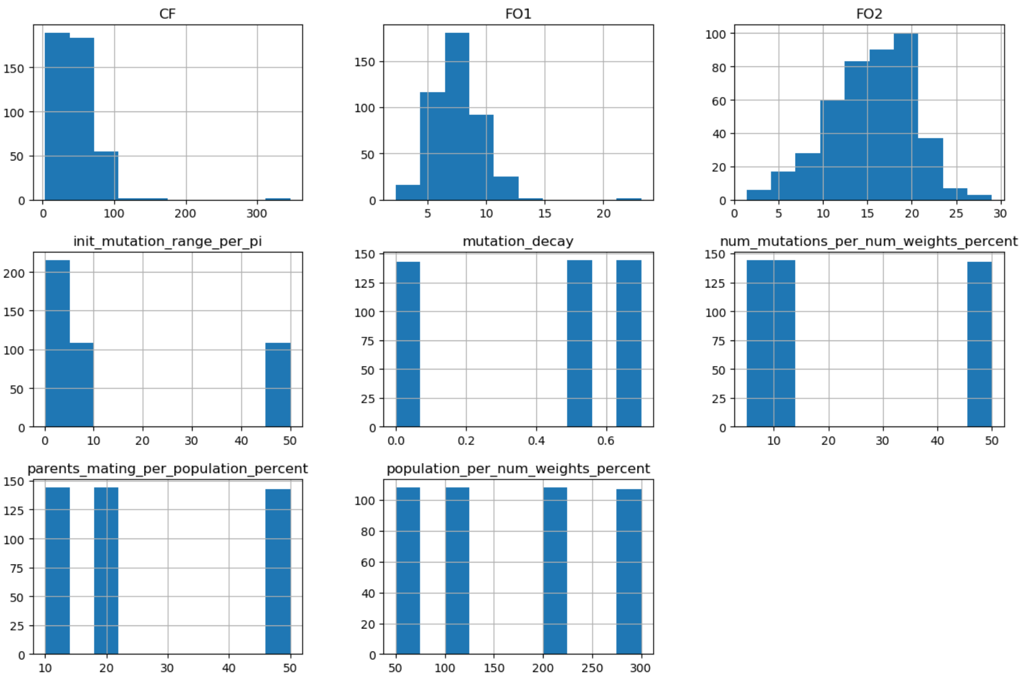

Supplementary Figure 26 | Optical filter routing function grid search results with genetic algorithm for $CF^{Optical\ filter}{}_{1}$: Histogram including all datasheets.

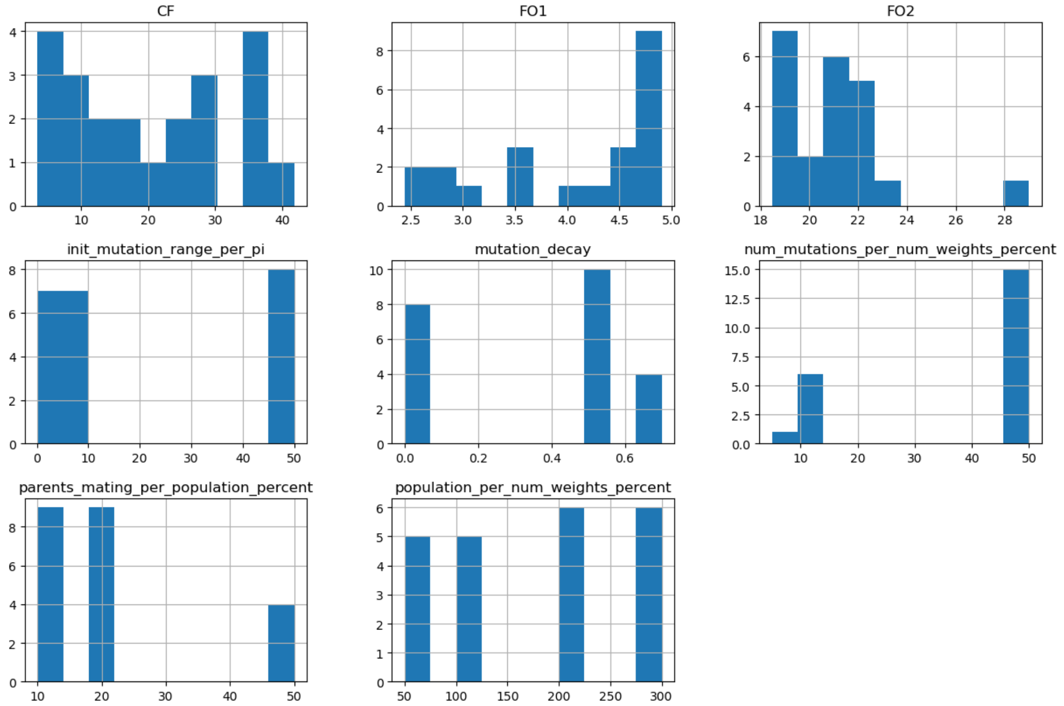

Supplementary Figure 27 | Optical filter routing function grid search results with genetic algorithm for $CF^{Optical\ filter}_1$: Histogram including the best performance datasheets.

A 5.55 % of the samples have achieved the targeted values during the self-configuration task. This reflects both the dependence of the hyperparameter values with the completion of the task and that the hyperparameter ranges selected are too broad. We limited the number of operations to 3000 for every trial, so if the experiment does not converge is considered as failed. It is worth nothing that each operation implies a single configuration of the waveguide mesh and the extraction of the amplitude scattering matrix at only one optical channel defined by the optical ports 4 and 1. In order to test the statistical variability of the method we run 100 experiments with different initial conditions as phase offsets and loss distributions of the waveguide mesh arrangement, employing the hyperparameters from Supplementary Table . The results are included in Supplementary Figure 28.

Supplementary Table 21 | Selection of best parameters employed for the statistical analysis.

**$(CF^{all\text{-}cross}_1)$**	**Best performance ranges**
mutation_decay	0.5
parents_mating_per_num_weights_percent	25%
population_per_num_weights_percent	200%
num_mutations_per_num_weights_percent	50%
init_mutation_range_per_pi	50%

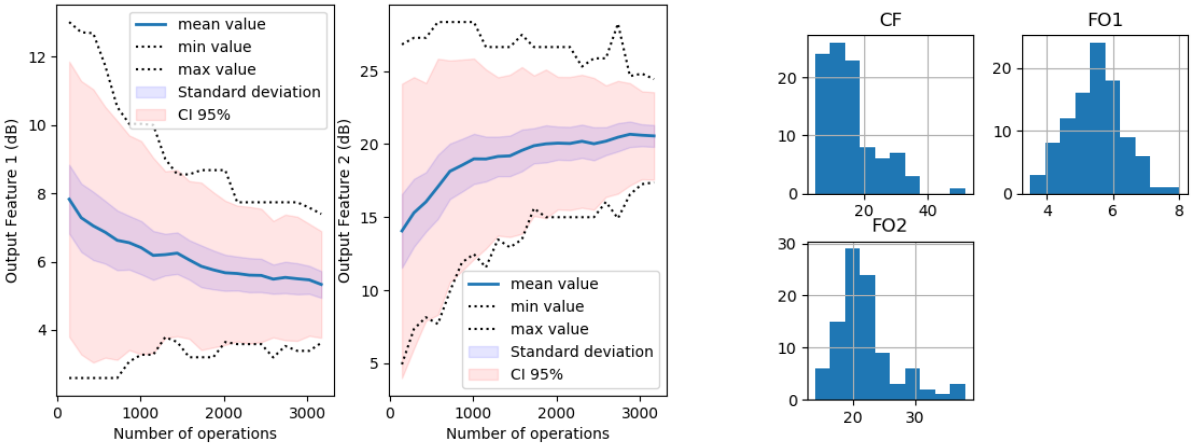

Supplementary Figure 28 | Optical filter function statistical results for fixed hyperparameter selection with GA algorithm for $CF^{O.Filter}_1$: Evolution of the output features (OF1: Insertion loss of the passband(dB), OF2: Extinction Ratio (dB). Progress (left) and histogram at last iteration (right) The datasheet is composed of 100 independent experiments with different waveguide mesh initial conditions. Statistical illustration of the circuit.

We can then verify the spectral response statistically plotting the mean, standard deviation and interval of 95% confidence, as shown in Supplementary Figure 29. In this particular hyperparameter selection the 25% of the trials achieved the targeted metrics (*OF*1 better than 5 dB and *OF*2 better than 18 dB). The mask is accomplished notably, considering that achieving the targeted spectral response might require a high-order filter that can be achieved by cascading more components and thus requiring a larger-scale circuit. To achieve the targeted insertion loss while maintaining the conditions fixed for the stopband and the passband in terms of flatness, and bandwidth. We believe that a large-scale waveguide mesh architecture will offer more freedom to combine and split the light and thus achieve more challenging optical filter design demands.

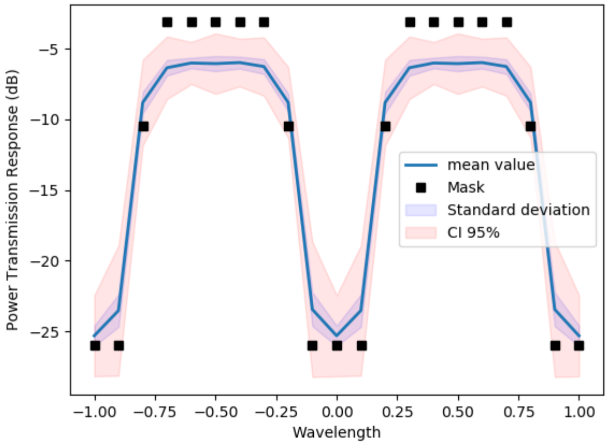

Supplementary Figure 29 | Optical filter function statistical results for fixed hyperparameter selection with GA algorithm for $CF^{O.Filter}_1$: Statistical results considering the spectral response after the self-configuration of the filter. The datasheet is composed of 100 independent experiments with different waveguide mesh initial conditions.

Conclusions on the GA in the optical filter self-configuration example

We applied different combinations of hyperparameters to one cost function describing the operation of an optical filter defined by a spectral mask. We show that the success rate is dependent on the hyperparameter selection. In addition, we repeated 100 times a configuration test, varying the initial conditions or phase offsets in the waveguide mesh arrangement showing a success rate of 25% always employing less than 3000 operations. The convergence trends suggest that a greater number of operations would achieve better results, 100% of the results are very close to the global optimum.

- **Optical filter self-configuration employing a Particle Swarm Optimization (PSO) algorithm:**

As we saw for the previous examples, the efficiency of the PSO algorithm is dependent on the hyperparameters selected, the cost function and the targeted problem. We perform again a grid-search tuning the hyperparameters and running the algorithm for 320 trials for each of the proposed cost function. The hyperparameters are selected following the ranges specified in Supplementary Table to ensure that we explore different combinations. Note that in this cases all the hyperparameters are fixed. A better convergence speed and less sensitivity to the hyperparameters can be achieved if parameters like the inertia are configured adaptatively to decrease during the optimization process.

***$CF^{O.Filter}_1$*:**

Supplementary Table includes the correlation matrix of the datasheet containing the self-configuration examples. It suggests that in order to minimize the cost function and the output feature 1 and maximize output feature 2, one should employ a moderate *inertia* coefficient, and a moderately large *number of particles*. Regarding the *coginitive* and *social* coefficient, their linear relation is not well defined, although the social value has a larger linear dependency than the cognitive value within the range tested.

Supplementary Table 22| Correlation matrix after the reconfiguration of $CF^{O.Filter}_1$

**(CF^{1x8}_1)**	**CF**	**FO1**	**FO2**
**CF**	1.000000	0.825119	-0.474592
**FO1**	0.825119	1.000000	-0.397155
**FO2**	-0.474592	-0.397155	1.000000
inertia	0.685360	0.584035	-0.714336
cognitive	-0.027732	0.000538	0.038607
particles_per_num_weights_percent	-0.141062	-0.086131	0.112652
social	-0.070270	-0.047905	0.113850

To delve into the actual performance of the different combinations, Supplementary Figure 30 and Supplementary Figure 31, illustrate the histogram of the complete datasheet and the datasheets featuring the targeted performance, respectively. The second datasheet

includes thus the best results accomplishing OF_1 better than 4.5 dB and OF_2 better than 18 dB. From the data, we infer that the ideal values are the ones summarized in the next Supplementary Table.

Supplementary Table 23 | Selection of best performance hyperparameters

**(CF^{1x8}_{1})**	**Best performance ranges**
Number of particles per weight %	200%
Inertia coefficient	0.5
Cognitive coefficient	0.5-1
Social coefficient	1-2

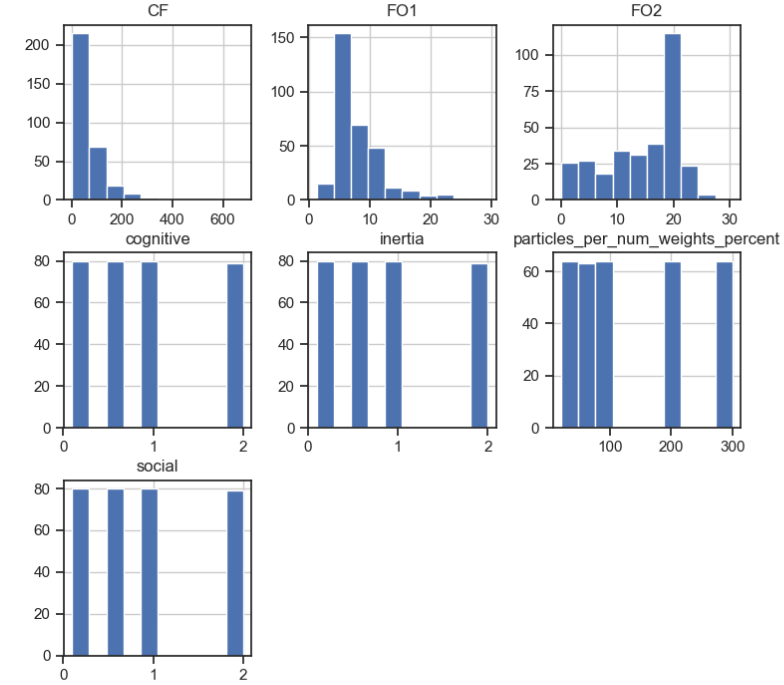

Supplementary Figure 30 | Optical filter grid search results with PSO algorithm for $CF^{O.Filter}_1$: Histogram including all datasheets.

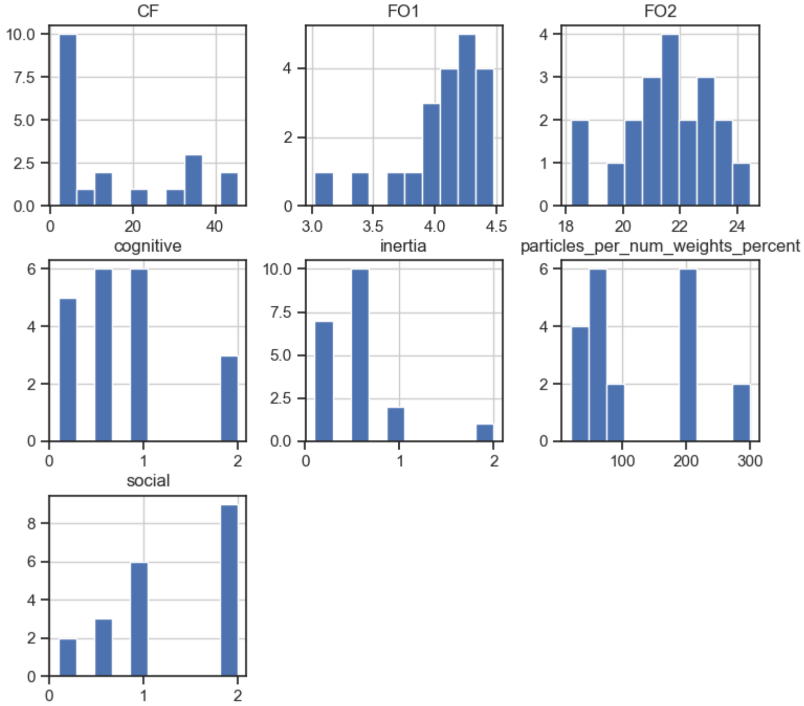

Supplementary Figure 31 | Optical filter grid search results with PSO algorithm for $CF^{O.Filter}_1$: Histogram including the best performance datasheets.

In this case, the 6.25% of the samples completed successfully the self-configuration task. This only reflects both the dependence of the hyperparameter values with the completion of the task and that the hyperparameter ranges selected are too broad. We limited the number of operations to 3000 for every trial, so slower convergence samples might be considered as failed. It is worth nothing that each operation implies a single configuration of the waveguide mesh and the extraction of the amplitude scattering matrix at only one output channel.
In order to test statistically the success rate for a fixed set of "optimum" hyperparameters, we test the method 100 times with variable initial conditions for the waveguide mesh arrangement. The results plotted in Supplementary Figure 32 and Supplementary Figure 33 illustrate that 71% of the samples achieve the targeted performance. In addition, the trends suggest that all the trials are close to the global optimum and a large number of operations would meet the requirements.

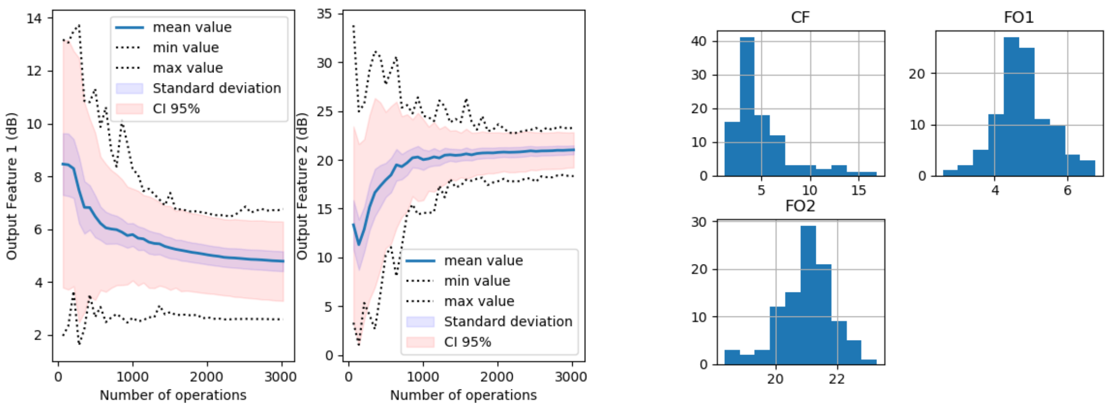

Supplementary Figure 32 | Optical filter function statistical results for fixed hyperparameter selection with PSO algorithm for $CF^{O.Filter}_{1}$: Evolution of the output features (OF1: Insertion loss of the passband(dB), OF2: Extinction Ratio (dB). Progress (left) and histogram at last iteration (right) The datasheet is composed of 100 independent experiments with different waveguide mesh initial conditions.

We can then verify the spectral response statistically consider the final configuration for each trial. The mask is accomplished notably, and it shows that the device finds challenging achieving the targeted insertion loss while maintaining the conditions fixed for the stopband and the passband in terms of flatness, and bandwidth. We believe that a large-scale waveguide mesh architecture will have more freedom to combine and split the light and thus achieve more challenging optical filter design demands.

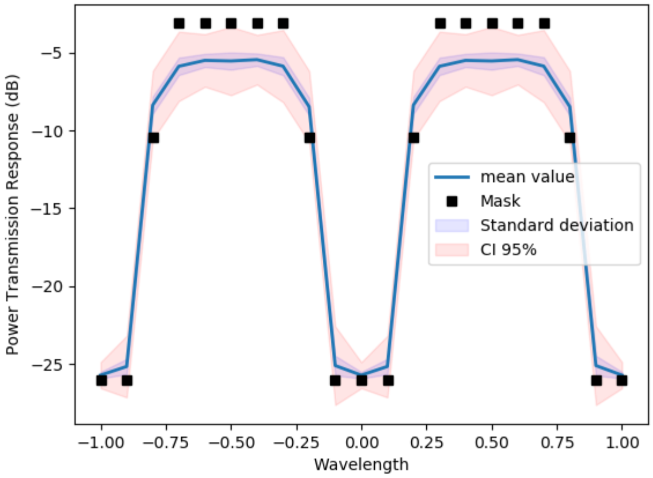

Supplementary Figure 33 | Optical filter function statistical results for fixed hyperparameter selection with PSO algorithm for $CF^{O.Filter}_{1}$: Statistical results considering the spectral response after the self-configuration of the filter. The datasheet is composed of 100 independent experiments with different waveguide mesh initial conditions.

The flexibility of the self-configuration approach is demonstrated with the following application examples. In all of them, we maintained the hyperparameters employed in the previous example. For each figure, the datasheet employed is specified. Every trial is statistically independent from each other and we consider random an unknown offset value for each TBU. In all of them, ports 1 and 4 are employed. First, Supplementary Figure 34 includes the spectral responses and progress for different spectral masks. The first three examples maintain the targeted free spectral range, corresponding to a 2-TBU difference or cavity. The resolution (understood as the number of samples of the spectral response) is low. We can see how the modification of the four points at -10 dB from the first, second and third subfigure, modify the passband and stopband responses. The four example targets a different free spectral range, related to a 4-TBU difference or cavity length. It finds the challenge of spectral filters when trying to optimize the extinction ratio in a single frequency

while maintaining a flat passband with low insertion loss. In this case, a mean ripple of less than 2 dB is produced to achieve a narrow stopband.

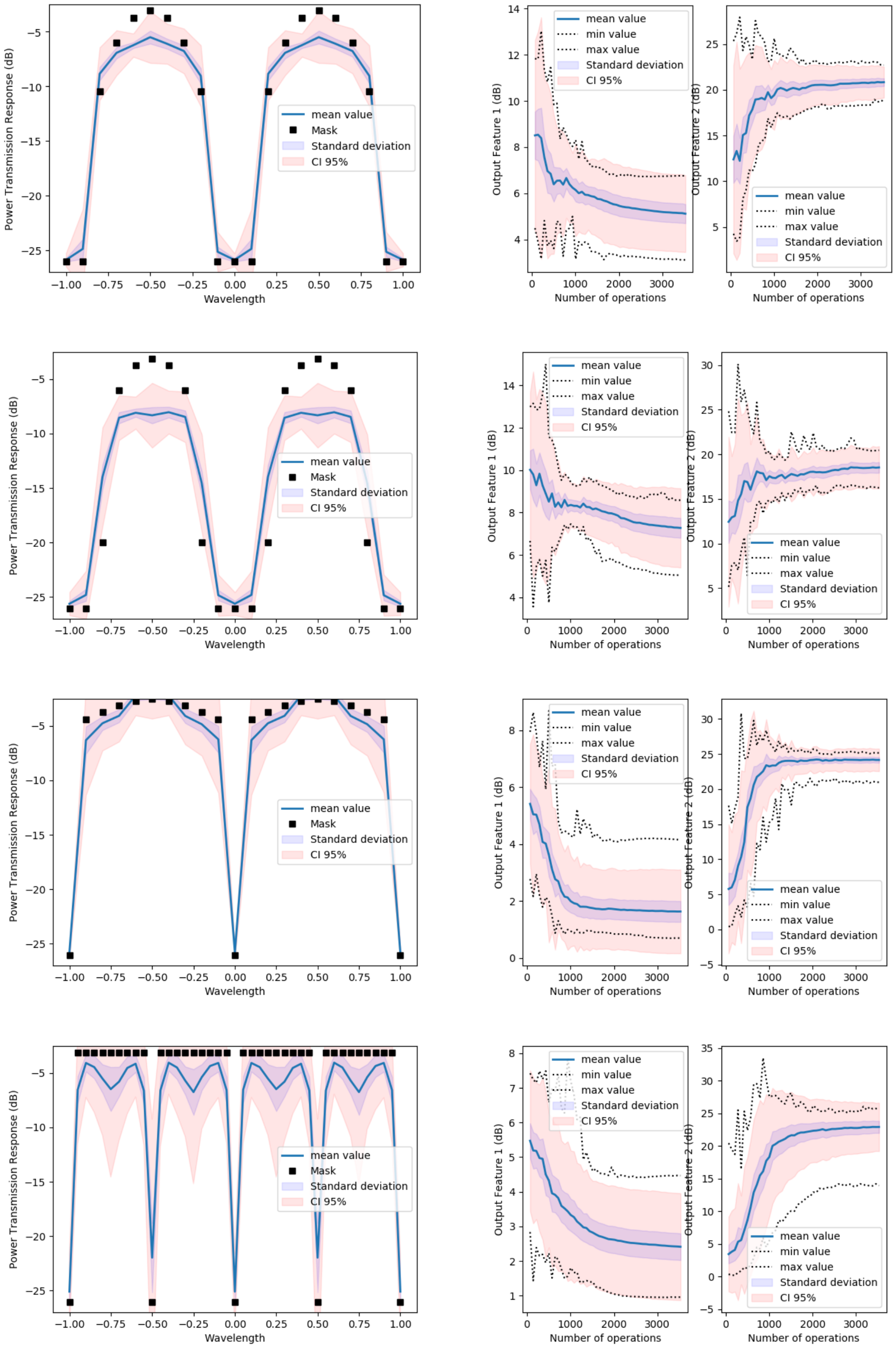

Supplementary Figure 34 | Optical filter function statistical results for fixed hyperparameter selection with PSO algorithm for $CF^{O.Filter}_{1}$: Spectral response, Evolution of the output features (OF1: Insertion loss of the passband(dB), OF2: Extinction Ratio (dB). Each datasheet is composed of 20 independent experiments with different waveguide mesh initial conditions.

The great advantage of multipurpose meshes is that we can, maintaining the same inputs and outputs as in the previous example, configure additional spectral masks with different free spectra range. The following examples illustrated in Supplementary Figure 35, configure different masks while maintaining a free-spectral range associated to 6-TBU interferometric lengths.

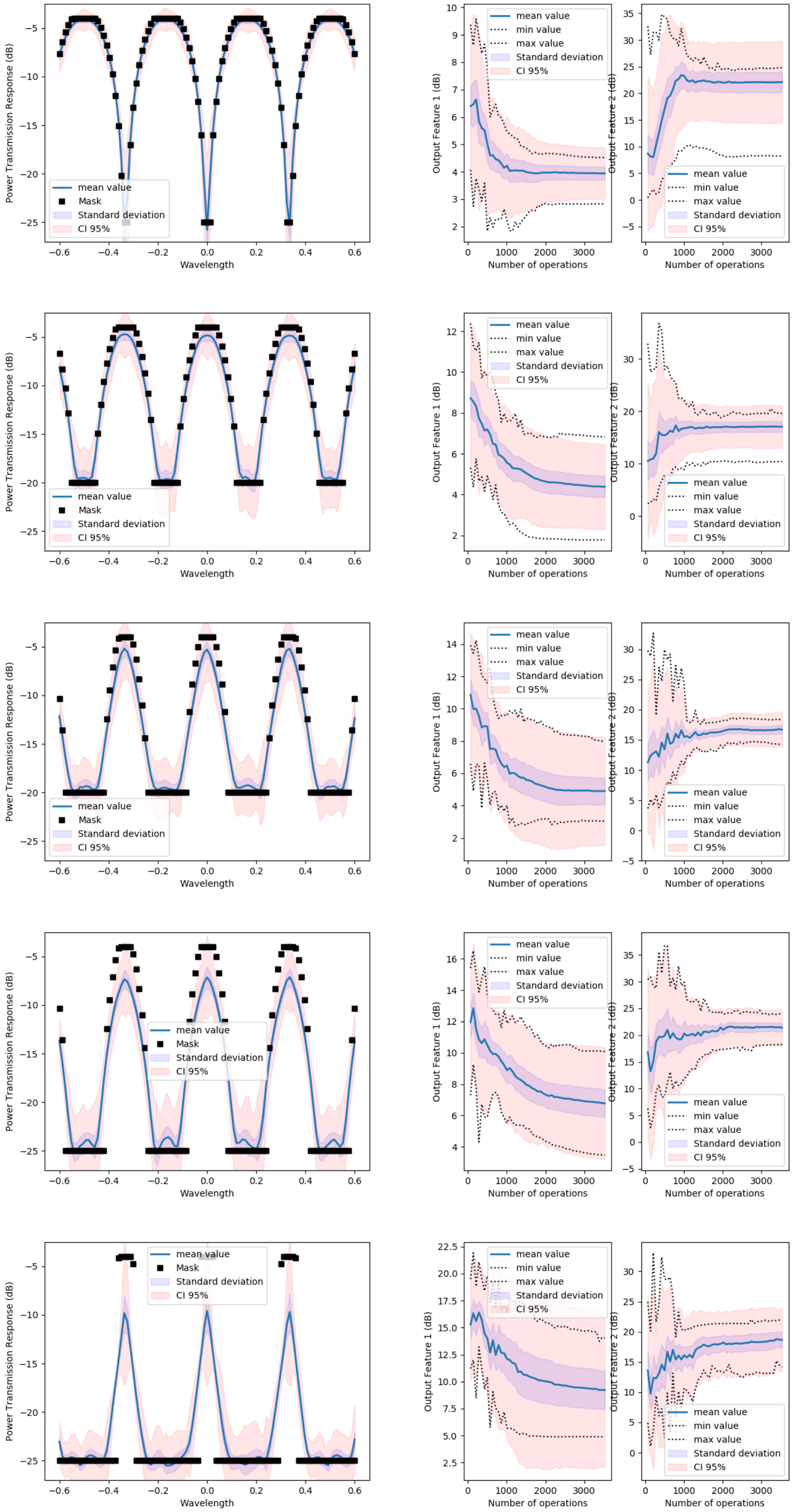

Supplementary Figure 35 | Optical filter function statistical results for fixed hyperparameter selection with PSO algorithm for $CF^{O.Filter}_1$: Spectral response, Evolution of the output features (OF1: Insertion loss of the passband(dB), OF2: Extinction Ratio (dB). Each datasheet is composed of 20 independent experiments with different waveguide mesh initial conditions.

The first example is a mask that resembles the reflexion response of a ring resonator with the notch centred at normalized frequency equal to 0, and achieves an average extinction ratio of 22 dB, and an average insertion loss of 4 dB after the self-configuration process. The spectral mask is achieved notably. The second example targets a more selective filter. In this case, the mask reduces the passband region and increase the stopband range. The extinction ratio specifications are reduced to 16 dB. The remaining examples continue both reducing the passband range and increasing the stopband region to find a more selective filter. To meet the specifications the self-configuration routine returns a filter with higher insertion loss. In the last two cases the trends of the progress lines suggest that a large number of iterations would improve the system performance in both output features.

As shown in Supplementary Figure 36, we can change the mask to alternative free spectral ranges including 8, 6, 10 -BUL interferometric paths. When more periods are obtains, it is necessary to either increase the number of wavelength points or reduce the frequency range where the mask is evaluated to ensure that we have enough resolution to resolve the spectral response.

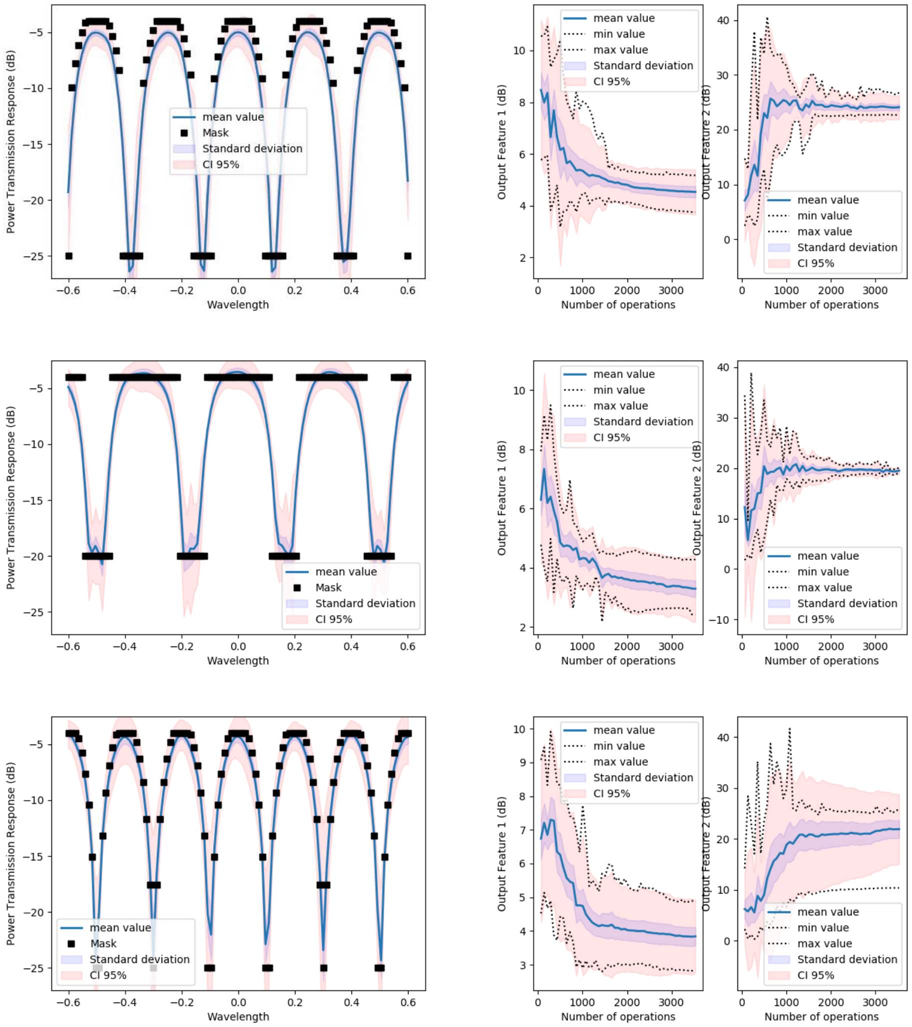

Supplementary Figure 36 | Optical filter function statistical results for fixed hyperparameter selection with PSO algorithm for CF$^{\text{O.Filter}}{}_1$: Spectral response, Evolution of the output features (OF1: Insertion loss of the passband(dB), OF2: Extinction Ratio (dB). The datasheet is composed of 20 independent experiments with different waveguide mesh initial conditions.

- **Optical Filter self-configuration on waveguide mesh arrangements with non-ideal components:**

The scalability of multipurpose waveguide mesh arrangement to higher integration densities is currently severely restrained by several factors arising from physical hardware constraints and the precise control and configuration of several hundreds of variables. One of the advantages of programmable multipurpose waveguide meshes is, however, their potential management of non-ideal fabrication and design defects in the circuit in an automated way. The main non-ideal effects are the uneven distribution of optical loss all over the circuit, the optical crosstalk and parasitic effects, and the dynamic crosstalk coming from the undesired tuning mechanism effects in neighbouring photonic components [1] [3] [16]. Some of these effects can be mitigated by using pre-characterization routines and modelling the effects to counter-act them in during the configuration stage. However, obtaining the full characterization of some of them, for example the tuning-crosstalk, is a time-consuming and difficult task that get worse for large-scale circuits. For example, obtaining the tuning crosstalk matrix that computes the crosstalk coefficient between N phase actuators all over the circuit requires the

computation of N^2 coefficients. Moreover, it is still not clear if these coefficients are dependent on the number of phase actuators that are enabled at a given slot of time, and if other areas of the circuit are affected by this effect.
As explained in **Supplementary note 4,** the self-configuration routines proposed in this work, assumes these non-ideal effects as part of the system behaviour and consider their effects during the optimization task. Both passive effects like non-uniform loss distribution all over the circuit, optical crosstalk, and active defects like environmental conditions fluctuations or dynamic tuning crosstalk are considered. In the following examples, we demonstrate the application of the aforementioned routines to non-ideal circuits providing fault-tolerant, self-healing and error-mitigation capabilities.

Tuning crosstalk:
When tuning one phase actuator, for example a thermo-optic actuator, the physical effect causing the tuning in the desired waveguide can spread to the neighbouring waveguides producing an undesired tuning effect. In the worst cases, even at distances larger than 10 mm the tuning crosstalk effect can be appreciated [3]. The tuning crosstalk can be modelled by a constant that reflects the percentage of phase shift occurred in the non-targeted waveguide compared to the experienced by the target waveguide [4]. Simulations and experimental works result in a crosstalk coefficient between 1 and 3% at several hundreds of micrometers. If extended to a system with multiple phase shifters, this model can be extended to a system of equations relating the effective phase shifts with the phase shifts set by the algorithm or the user.

$$\Delta\phi_{effective} = \begin{pmatrix} 1 & CT_{12} & \cdots & \cdots & CT_{1N} \\ CT_{21} & 1 & & & CT_{2N} \\ CT_{31} & & 1 & & \vdots \\ \vdots & & & \ddots & CT_{1N-1} \\ CT_{N1} & CT_{N2} & \cdots & \cdots & 1 \end{pmatrix} \begin{pmatrix} \Delta\phi_1 \\ \Delta\phi_2 \\ \vdots \\ \\ \Delta\phi_N \end{pmatrix} \tag{S9}$$

To test the algorithm, we perform two statistical test. The first one does not include the thermal crosstalk while the second one is affected by it. In the latter, we load to the performance model [1] a severe crosstalk matrix where the crosstalk coefficients are obtained from a uniform distribution from 0 to 5%. Moreover, the crosstalk matrix does not contemplate the mitigation of the crosstalk with the distance, making overall a more challenging configuration scenario. As shown in Supplementary Figure 37 the differences are inappreciable, as the self-configuration routine consider the dynamic tuning crosstalk effect during the optimization procedure.

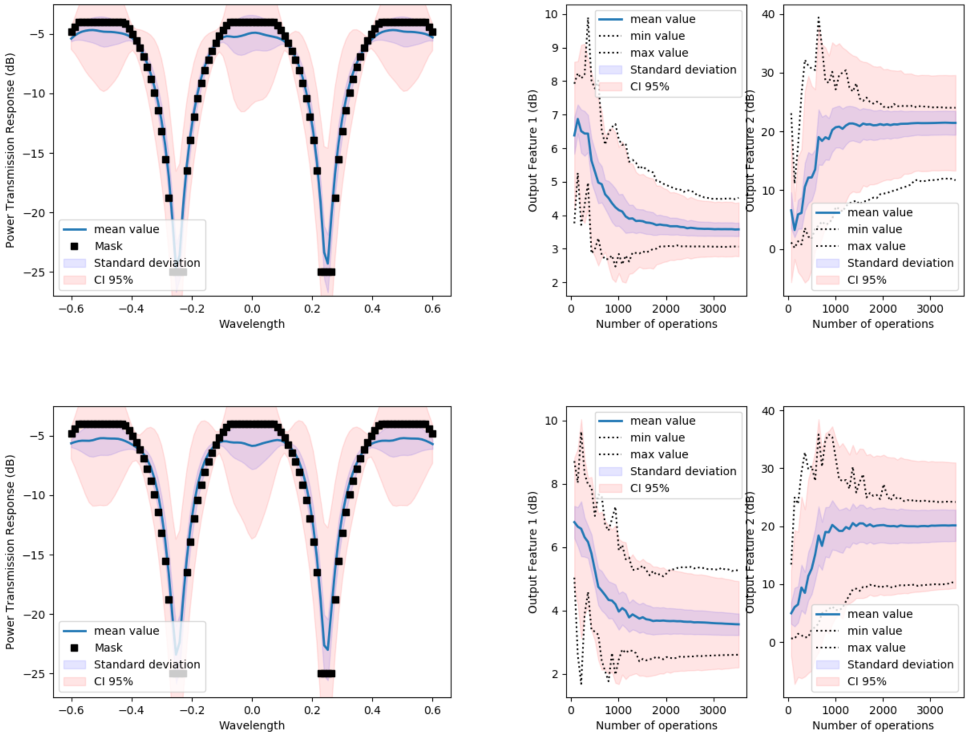

Supplementary Figure 37 | Optical filter function statistical results for fixed hyperparameter selection with PSO algorithm for $CF^{O.Filter}_1$: Spectral response, Evolution of the output features (OF1: Insertion loss of the passband (dB), OF2: Extinction Ratio (dB). The datasheet is composed of 20 independent experiments with different waveguide mesh initial conditions. Upper example: no crosstalk, Lower example: crosstalk 5%

We can repeat the same trial with a different structure. In this case, we change the mask and perform the test without crosstalk, with a crosstalk of 5% and with a crosstalk of 10%. Highlighting again that these scenarios are by far, more challenging that the ones experienced by a real system, the average performance of the self-configuration routine is remarkably good. These results open the possibility for employing waveguide mesh arrangements with much higher integration density, reducing the distance between components [11].

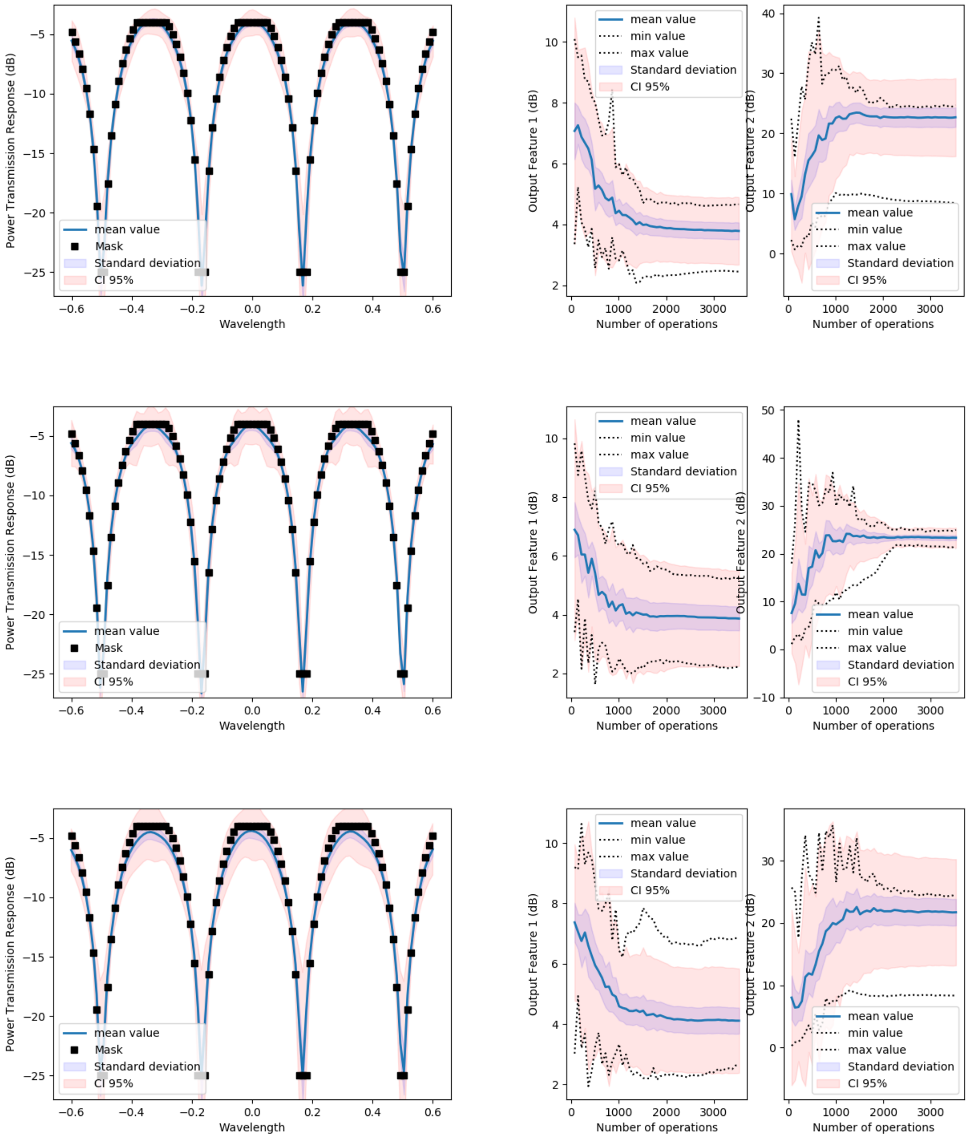

Supplementary Figure 38 | Optical filter function statistical results for fixed hyperparameter selection with PSO algorithm for $CF^{O.Filter}_1$: Spectral response, Evolution of the output features (OF1: Insertion loss of the passband (dB), OF2: Extinction Ratio (dB). The datasheet is composed of 20 independent experiments with different waveguide mesh initial conditions. Upper example: no crosstalk, Middle example: crosstalk 5%, Lower example: crosstalk 10%

Fault-tolerant and self-healing effects:

In some cases, the fabrication defects or derive of components can decrease the performance of the components integrated in the circuit, even destroying completely one section of the circuit. Whereas these fabrication and design yields can completely discard a whole die in application specific photonic integrated circuits, mesh arrangements offer potential fault-tolerant and self-healing capabilities. This approach comes from the architecture of these circuits, which rely on the repetition and interconnection of simple components. The availability of spare components and sections in the circuit enable the use of alternative circuits when some parts of the circuit are damaged. To illustrate a demonstration example, we show in Supplementary Figure 39 the waveguide mesh arrangement under use. In this case, we configure the filter specified by the spectral mask specified in the figure. Given the demanded FSR one could expect that

the cells numbered as 1,2, 3 and 4 have more probabilities to perform as a coupled cavity and define the targeted mask. In the next example, we decrease the performance of TBUs A6, B6 and C6 by imposing 30 dB insertion loss to each one. After running the statistical test again, the self-configuration process is able to provide alternative structures. Indeed, we can see that it is able to maintain notably the demanded response, probably employing the cavities 5 and less likely 6.

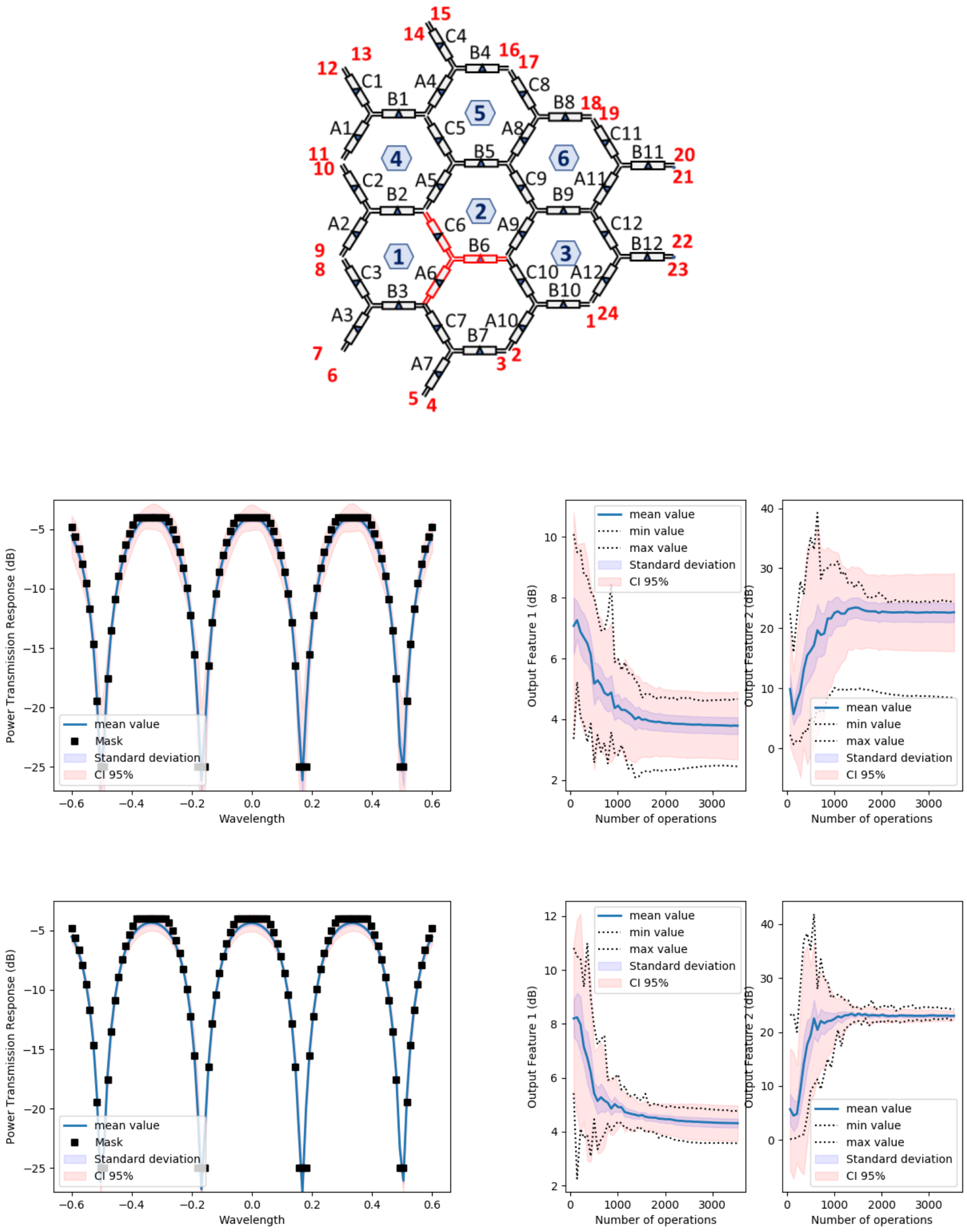

Supplementary Figure 39 | Optical filter function statistical results for fixed hyperparameter selection with PSO algorithm for $CF^{O.Filter}_1$: Spectral response, Evolution of the output features (OF1: Insertion loss of the passband(dB), OF2: Extinction Ratio (dB). The datasheet is composed of 20 independent experiments with different waveguide mesh initial conditions. (up) mesh with good performance, (down) mesh with TBUs A6, B6, C6 featuring additional 30 dB insertion loss.

Check the Supplementary Note 10 for the analysis of the impact on the FSR deviations on the self-configuration process and an alternative way to define more complex cost-functions.

Supplementary Note 8: Description of the laboratory set-up

In this subsection, we describe the laboratory set-up under use in the experiments and further reflect on the importance of choosing appropriately the cost function, this time in a realistic scenario.

Supplementary Figure 40 sketches the 30-TBU waveguide mesh under use in our experiments (See details [3, 24]). Apart from it, our measurement set-up consists of a synchronized tunable laser source, a Multichannel Electronic Driver Array (MEDA) subsystem based on a table-top multichannel current source along with an optical spectrum analyser (OSA) as an optical monitor with custom routines in Python being run on a standard personal computer thus completing the entire closed-feedback loop along with a processing unit. This set-up allows us to achieve a spectral resolution of 1 pm and to ensure a minimum of 50 points per period for the shortest-FSR structure, thus resolving the spectral traces -mostly periodic filters whose FSRs are inherently limited to a set of architecture-dependent discretized values (which range in our case from 297 to 50 pm)- with more precision.

Prior to our first experiment, we synthesized the optical path connecting 'in' and 'out' ports appearing in Supplementary Figure 40(a) from a pre-defined current setting. Then, we performed a current sweep on TBUs H11 and H16 to draw the plot contours appearing in Supplementary Figure 41 and Supplementary Figure 42, in which their height values are expressed by means of the cost functions defined in Supplementary Figure 40(c). All such functions apply a brute maximization of the optical signal's output power in its central wavelength ($CF_{1\text{-}3}$) or averaging over its whole spectrum ($CF_{4\text{-}6}$). At the same time, $CF_{1,4}$ are expressed in dB, while $CF_{2,5}$ and $CF_{3,6}$ appear in linear units.

In both Supplementary Figure 41 and Supplementary Figure 42 we observe no significant differences between choosing the central element of the spectrum or averaging over all the samples captured by the OSA. As observed, representing the output power in linear units leads to more pronounced slopes compared to the smoothed ones obtained through conversion to logarithmic scale. Intuitively, this should help to speed up convergence for first-order optimization algorithms, as the calculated gradients at each point will present much larger norms.

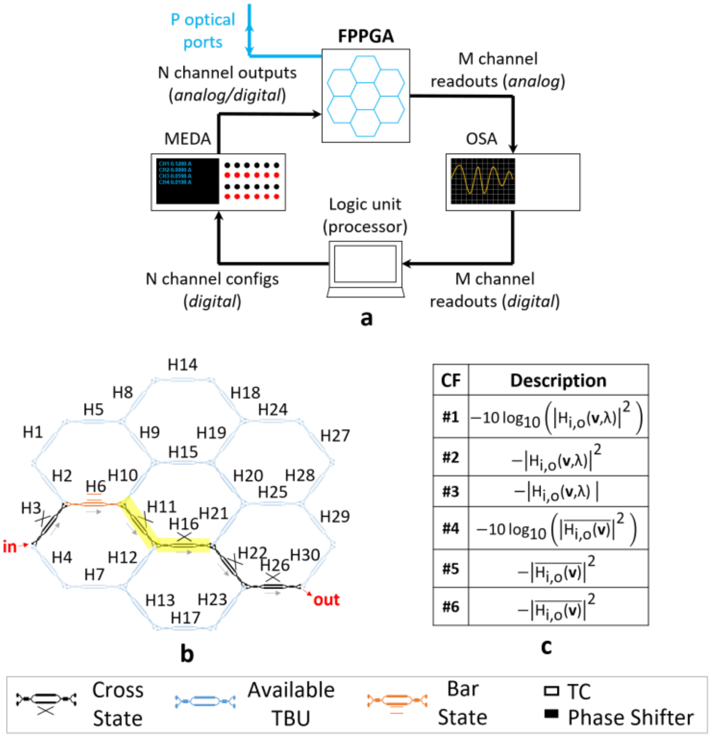

CF	Description
#1	$-10\log_{10}\left(\lvert H_{i,o}(\mathbf{v},\lambda)\rvert^2\right)$
#2	$-\lvert H_{i,o}(\mathbf{v},\lambda)\rvert^2$
#3	$-\lvert H_{i,o}(\mathbf{v},\lambda)\rvert$
#4	$-10\log_{10}\left(\lvert \overline{H_{i,o}(\mathbf{v})}\rvert^2\right)$
#5	$-\lvert \overline{H_{i,o}(\mathbf{v})}\rvert^2$
#6	$-\lvert \overline{H_{i,o}(\mathbf{v})}\rvert^2$

Supplementary Figure 40 | (a) Sketch of our experimental set-up, consisting of a MEDA subsystem based on a table-top multichannel current source (supplying thirteen independent channels) along with an Optical Spectrum Analyser (OSA) as an optical monitor with custom routines in Python being run on a standard personal computer. (b) Labelled schematic of the waveguide mesh arrangement under test and a synthesized optical path traversing TBUs H3, H6, H11, H16, H22 and H26. (c) List of cost functions (CF) under use to generate the contour plots from Supplementary Figure 41 and Supplementary Figure 42.

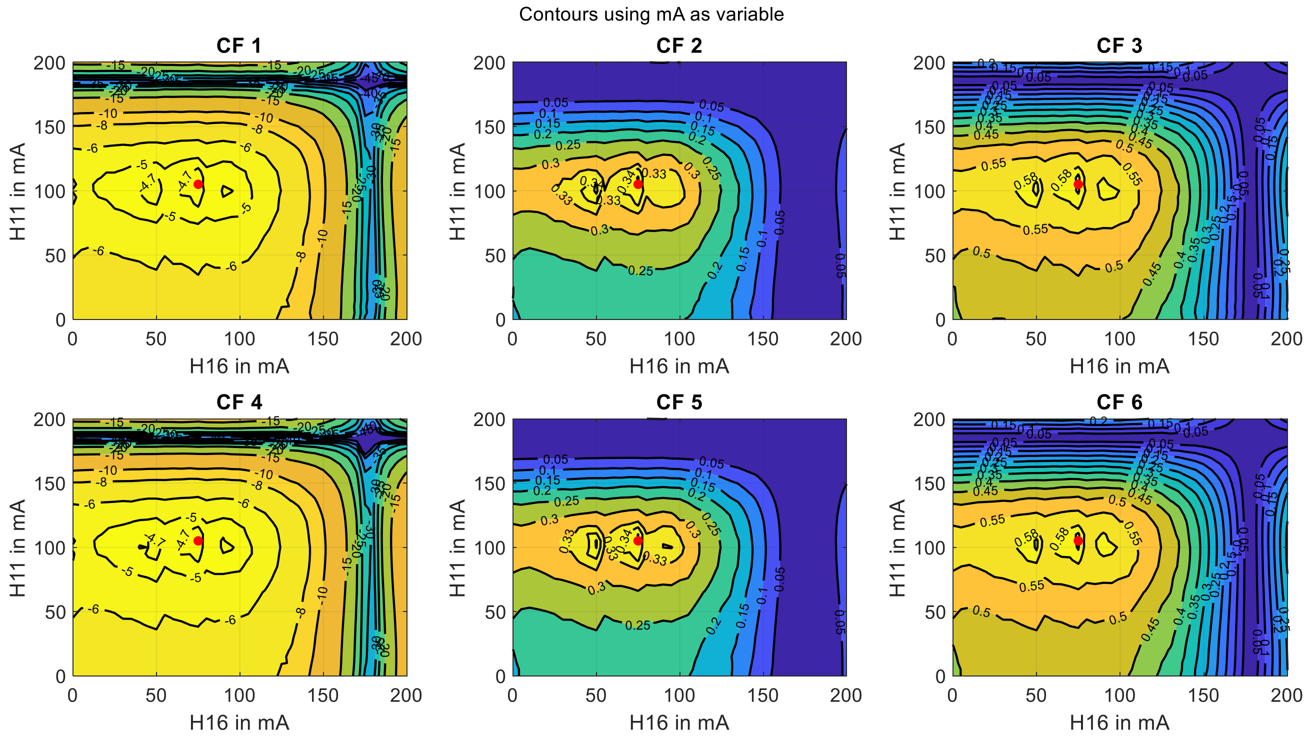

Supplementary Figure 41 | Contour plots of TBUs H11 and H16 using I (in mA) as a variable in the synthesis of the optical path from Supplementary Figure 40(a) for the cost functions defined in Supplementary Figure 40(b).

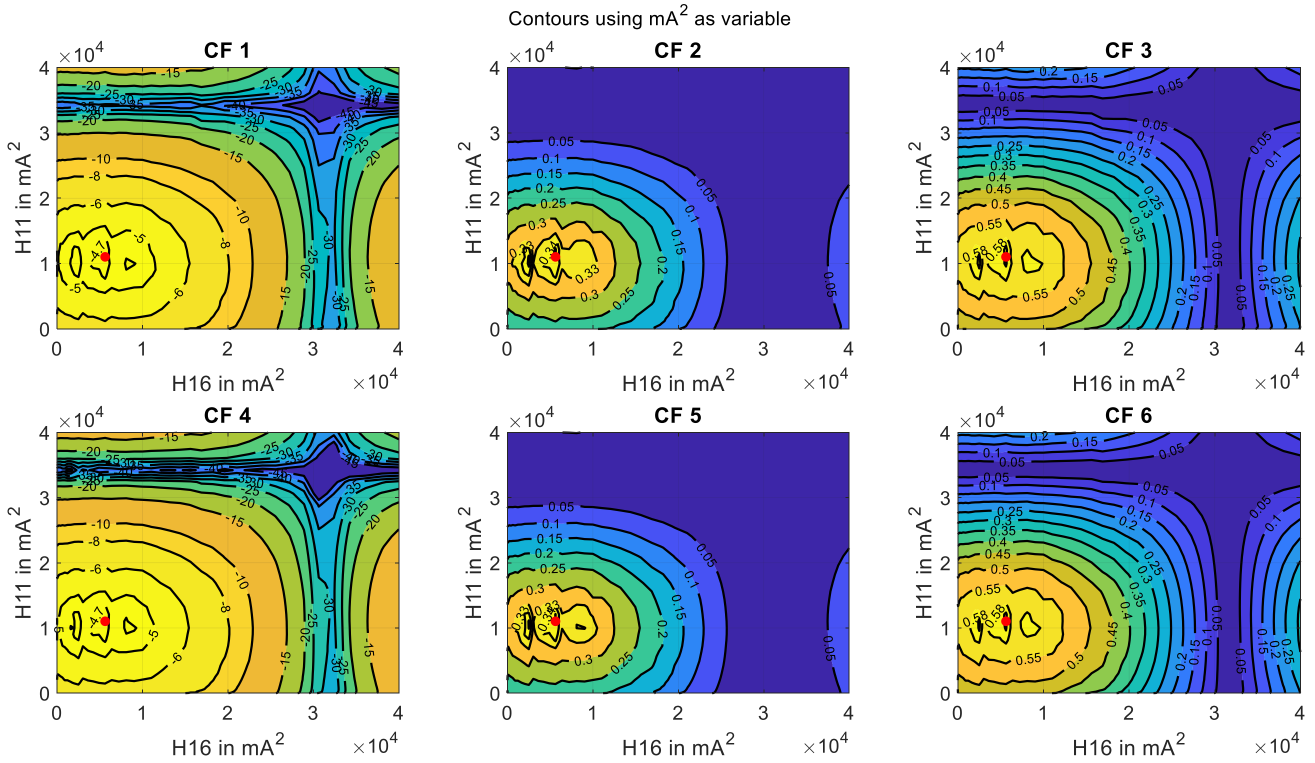

Supplementary Figure 42 | Contour plots of TBUs H11 and H16 using I2 (in mA^2) as a variable in the synthesis of the optical path from Supplementary Figure 40(a) for the cost functions defined in Supplementary Figure 40(b).

Supplementary Note 9: Experimental synthesis of optical circuits in a 30-TBU waveguide mesh

Here we report the synthesis of many optical paths in the same waveguide mesh than for previous section, starting from the same input and output nodes. This time we operate over 13 TBUs: H3, H6, H7, H10, H11, H12, H13, H16, H17, H21, H22, H23 and H26. Operating over such a large number of TBUs will allow us not only to effectively connect input and output ports, but also to reduce leakage by impeding light to be recirculated by surrounding TBUs. Both actions combined will lead to a maximization of the optical power at the output. In all cases, the starting point has been 'all-passive', meaning that all actuators were initially set to 0 mA. Supplementary Figure 43 illustrates some of the obtained results using both the current (I) and as optimization variable for derivative methods and PSO algorithm. The reason behind choosing optimizing over I rather than over I^2 lies on the fact that powering over two the space of variables has the effect of 'stretching' the values from the hyperparameter space, making more difficult to find appropriate coefficients for finishing the process successfully. For the choice of the cost function for derivative methods, we simply aimed to maximize the average output power in linear units between input and output optical ports (CF 5 from Supplementary Figure 41).

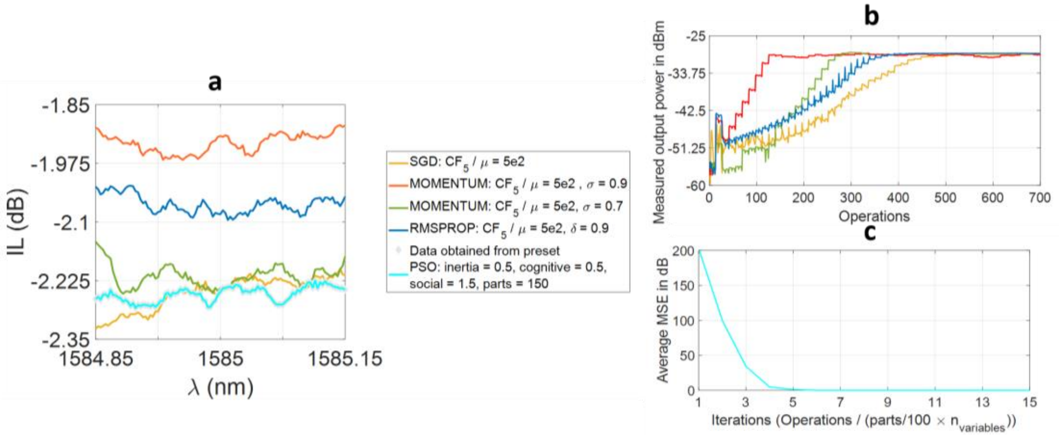

Supplementary Figure 43 | Experimental results of the synthesis of a 6-TBU optical path in our waveguide mesh using derivative optimization methods and PSO algorithm. (a) Experimental traces obtained from our optical spectrum analyser at the end of each process. A trace obtained from the use of current presets is also attached for comparison, **(b) Evolution of the average output power measured in each trace with the number of operations for derivative optimization methods**, **(c) Evolution of the average MSE between the synthesized path and the one obtained through current presets with the number of iterations for PSO algorithm.** An iteration includes the individual evaluation of each particle (operation) prior to the update of their velocities, and therefore of their positions.

In our second experiment, we proceeded to synthesize a 2-TBU MZI by monitoring the set of TBUs highlighted in yellow appearing in Supplementary Figure 44(a). The TBU highlighted in purple (H16) works under 'dual-drive' configuration, meaning that we are driving current into both of its upper and lower phase shift actuators to provide us independent control of its phase and amplitude response, something that will ultimately allow us to tune at pleasure both the extinction ratio and the wavelength notch of our filter. After being provided with the spectral mask of our MZI filter by using its corresponding preset of currents, we performed a set of experiments using PSO algorithm whose results are shown in Supplementary Figure 44(b,c). There, we can observe how the transmission spectrums of the new filters closely match that from the one obtained using current presents –as a matter of fact, one of them even features a slightly larger ER of around 30 dB. In all these experiments –and also during the following ones- we set an adaptive inertia parameter which lowered down progressively from 1 to 0.35. We also include a limitation of 40 mA (in absolute value) in the velocity of each particle's actuator to avoid strong variations in the particle's positions -especially at the beginning of the process, when this value of inertia is still very large. Each particle's actuator position has been also bounded between 0 and 200 mA to avoid any potential damage affecting the chip. The elapsed time for each experiment was of around two hours, due to the slow sweep of the laser employed (10 sec per operation). Next, we repeated the same procedure on a 6-TBU ORR, whose results appear in Supplementary Figure 45. Again, we obtained an ER of around 30 dB for this configuration.

Next, we aimed to reproduce a 10-TBU ORR by using the highlighted arrangement from Supplementary Figure 46. To do so, we increased the number of samples of our trace to 301 from the 101 used in our previous experiments prior to the execution of the algorithm, as we are dealing with a filter with smaller FSR –and therefore with a more 'challenging' spectrum to be captured by our optical spectrum analyser. We did not appreciate any positive impact from this on the final average MSE results of our synthesized filters with respect to the corresponding spectral mask, again obtained through an already known pre-set of currents. As observed in Supplementary Figure 46(b,c), results suggest that further increasing the number of operations would lead to a better matching between the obtained spectrum and the filter mask, which still features around 20 dB and a similar insertion loss in the passband for both synthesized structures. Speeding up the configuration of filters process can be done by employing broadband sources or faster tunable lasers.

Finally, we synthesized the 4-TBU MZI using the set of TBUs highlighted in yellow illustrated in Supplementary Figure 47. Unlike with previous experiments, this time we employed a self-made spectral mask in the same manner than with our simulations using the following expression:

$$H(\lambda) = 20\log_{10}\left(\left|\cos\left(\pi\frac{\lambda}{FSR} - \delta\right)\right|\right) \quad \text{(S10)}$$

in which both the FSR and the wavelength λ are expressed in nm. Phase variation δ does represent any arbitrary shift of the spectral response of our filter –which as mentioned shortly backwards, can be achieved through dual drive operation–, and it is set in our case to 0 rad. In addition, we flattened both passband and eliminated band regions (-1.5 dB in the passband and -28 dB in the stopband) of our mask to provide a slightly modified spectrum from the one that can be actually achieved using the set of TBUs at our disposal. Such flat spectral response would resemble more to the one provided by a higher-order filter rather than to the one supplied by our first-order MZI. In any case, we can observe from the figure how the algorithm 'does its best' to match the filter response to the spectral mask provided by the end user as much as it can. In accordance to simulated results in this work, the degree of similarity between both spectra is expected to increase if more electrical channels to drive a larger number of cell units are at our disposal. We performed this measurement on a different chip than the one used in Supplementary Figures 44-46, whose grating design was centred at 1570 nm rather than at 1585 nm. Looking at the variation of the measured MSE (Fig. 47c), it can be observed that it does not vary quite significantly compared to those from figs. 44-46. This happens due to the use of a larger number of sampling points during the experiment (501, in contrast to the 101 and 301 used in Supplementary Figures 44-45 and in Supplementary Figure 46 respectively). As a result, there is a much larger number of points close to the spectrum passband that contribute to reduce the average error, especially at early stages of the algorithm with the filter notches have not still been formed. Fig. 47(d), which illustrates the variation of the CF value with the number of operations, provides in this case a better insight of the performance of the algorithm under this scenario. Check the Supplementary Note 10 for the analysis of the impact on the FSR deviations on the self-configuration process.

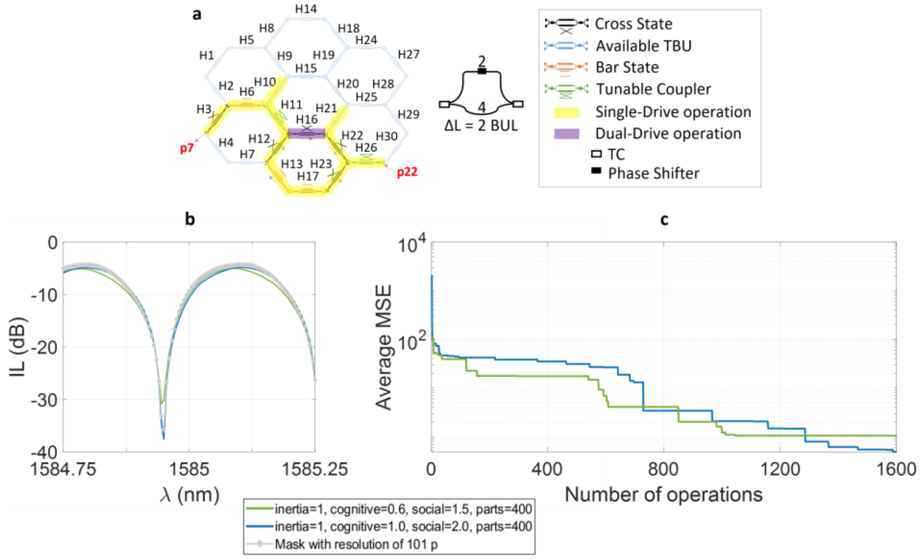

Supplementary Figure 44 | Experimental results of the synthesis of a 2-TBU unbalanced MZI in our waveguide mesh using PSO algorithm. (a) Schematic of the 30-TBU waveguide mesh. TBUs under use for the experiment appear highlighted in yellow and purple (dual-drive configuration), **(b) Final experimental traces obtained after executing the algorithm.** The spectral mask of the filter was obtained through current presets, **(c) Evolution of the average MSE provided by the algorithm with the number of operations.**

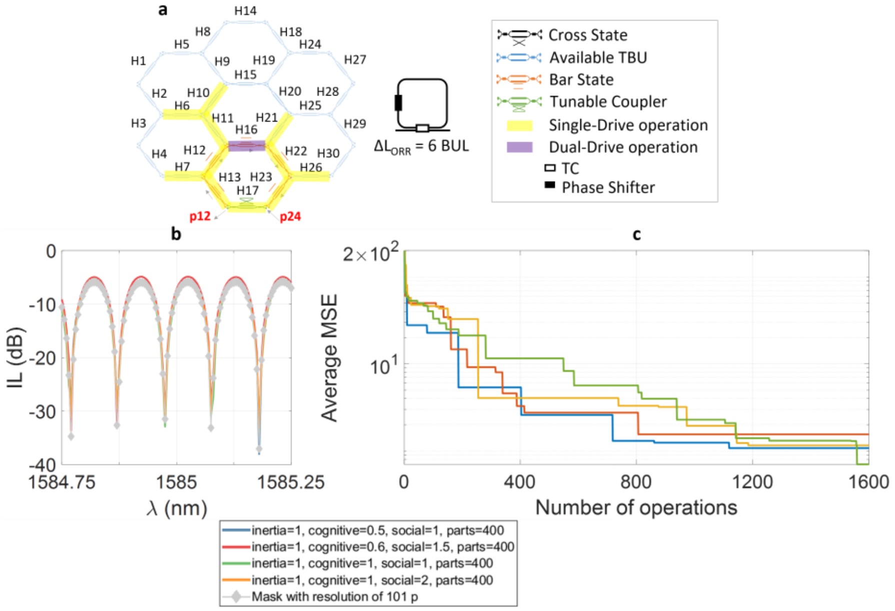

Supplementary Figure 45 | Experimental results of the synthesis of a 6-TBU ORR in our waveguide mesh using PSO algorithm. (a) Schematic of the 30-TBU waveguide mesh. TBUs under use for the experiment appear highlighted in yellow and purple (dual-drive configuration), **(b) Final experimental traces obtained after executing the algorithm.** The spectral mask of the filter was obtained through current presets, **(c) Evolution of the average MSE provided by the algorithm with the number of operations**.

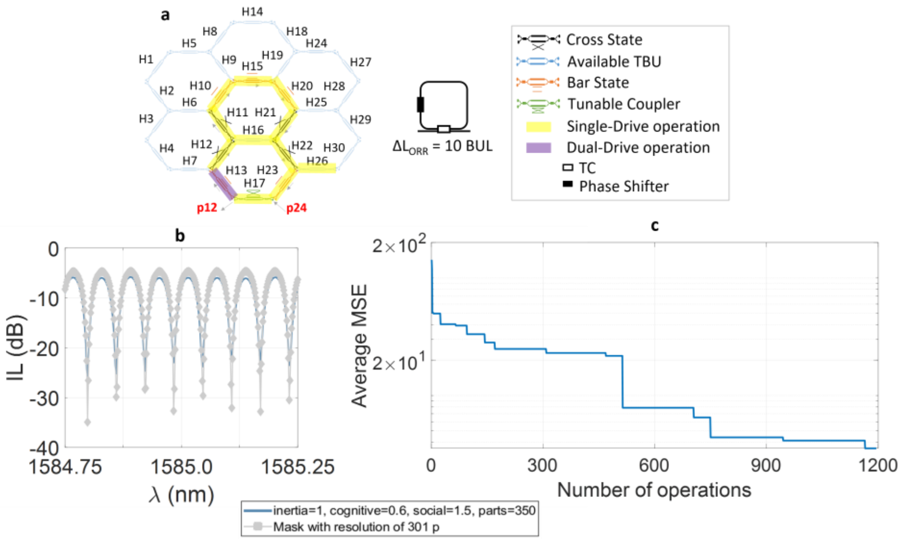

Supplementary Figure 46 | Experimental results of the synthesis of a 10-TBU ORR in our waveguide mesh using PSO algorithm. (a) Schematic of the 30-TBU waveguie mesh. TBUs under use for the experiment appear highlighted in yellow and purple (dual-drive configuration), **(b) Final experimental traces obtained after executing the algorithm.** The spectral mask of the filter was obtained through current presets, **(c) Evolution of the average MSE provided by the algorithm with the number of operations.**

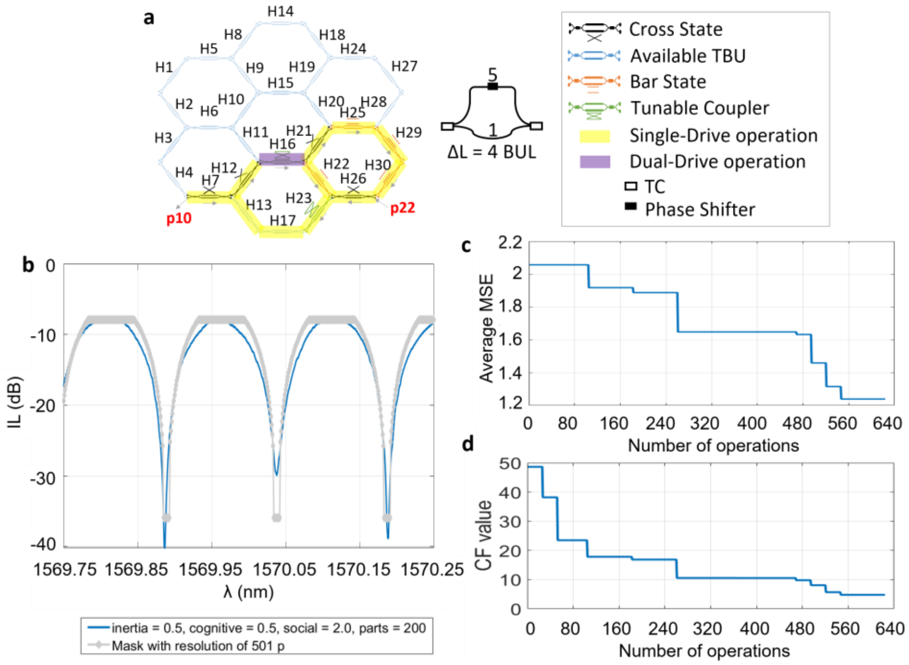

Supplementary Figure 47 | Experimental results of the synthesis of a 4-TBU imbalanced MZI in our waveguide mesh using PSO algorithm. (a) Schematic of the 30-TBU waveguide mesh. TBUs under use for the experiment appear highlighted in yellow and purple (dual-drive configuration), **(b) Final experimental traces obtained after executing the algorithm.** The spectral mask of the filter was obtained through a self-made spectral mask using the formula described in supplementary equation 10, **(c) Evolution of the average MSE provided by the algorithm with the number of operations**, **(d) Evolution of the CF value with the number of iterations**.

Supplementary Note 10: Main challenges for large-scale programmable photonic circuits.

Physics and engineering limit the future scalability of programmable photonic circuits employing a very large number of photonic actuators (>100-1000), waveguide loss and back reflection (discussed in Supplementary Note 7), and heater performance hamper the evolution of the technology. The scalability analysis of waveguide mesh arrangements was covered in [16, 21]. Here we extend the discussion for each of the main relevant scalability limits:

Fabrication defects, design deviation and other passive parasitic effects:

The high refractive index contrast of a silicon waveguide that makes it possible to confine light in a small volume, makes its behavior also very sensitive to small imperfections. Nanometer-scale geometry variations can already affect the circuit performance, limiting the scale of integration. These are mainly arising due to variations of the silicon thickness at the wafer-level and from deviations during the waveguide width patterning. On one hand, sidewall roughness can give rise to backscattering inside the waveguide, resulting in unwanted transmission fluctuations [1, 17]. On the other hand, small deviations of a few nanometers can lead to undesired changes in the light propagation properties, originating undesired phase deviations. This impacts over the performance of components like beam splitters, which are fundamental blocks of the programmable unit cells.

From a pure hardware perspective, several structures and mitigation techniques have been proposed and demonstrated as standalone components to reduce the impact of uniformity and risk of defects, and tuning crosstalk. For example, it has been demonstrated that the use of adiabatic directional couplers leads to better tolerances to waveguide geometry deviations [18] and that the use of ridge waveguide directional couplers with optimum geometry can cancel the effects due to width, gap and thickness deviations [19]. As far as thermal crosstalk is concerned, some demonstrations applied additional deep lateral air trenches [20] and optimized the architectural PIC design to facilitate the use of thermal crosstalk cancellation techniques [6].

As discussed in the main document and in the Supplementary Note 7 (Self-healing effects), the automated configuration methods reported in this paper overcome and mitigate the aforementioned defects, by finding optimal paths and avoiding the interaction with deteriorated waveguides or unit cells Future large-scale circuits will benefit from both fabrication-tolerant hardware and the self-healing attributes of the proposed configuration routines.

Power consumption, thermal crosstalk, and robustness of phase shifters:

Novel phase tuning mechanisms and architectures need to be optimized and developed to achieve low power consumption, low tuning crosstalk and robust phase actuators. The heater evolution trend in silicon on insulator shows a mitigation of the thermal tuning crosstalk and a reduction of the overall electrical power of the tuning elements. Beyond being beneficial for the overall power consumption of the circuit, it reduces the circuit complexity of the control electronics required for driving purposes. The current state of the technology is maturing to more robust driving and phase tuning actuators, but further improvements are required to achieve the consolidation of the technology. A good example of the trends followed by thermo-optic actuators in silicon photonic is the following:

- The simplest and original architecture is limited to proof-of-concept devices that employ a metal layer for both routing and heating (100 mW/pi, 450 µm-long to avoid electromigration and short life-times). Note this is the one employed in this paper, [3]
- Next, mature foundries have optimized the Joule-effect heater achieving better efficiencies and lengths through the improvement of their material qualities, processes, and thermo-optic waveguide geometries (30 mW/pi, 100 um), [22].
- In the last 5-8 years, additional techniques like the use of deep-trenches / isolation trenches, si-doped heaters are opening the path to better efficiencies (< 1mW/pi, 100 um) and a reduction of the driving circuitry complexity. With better efficiencies, the temperature of the heater can be reduced. In particular electromigration limits are inversely proportional to the electrical current density and the temperature [20].

To further reduce the power consumption, alternative phase tuning mechanisms are currently being explored in many research centers and universities. These include non-volatile tuning effects based on phase change materials, mems, and electro-optic effects. Together with thermo-optic effects, the technology needs to mature in terms of robustness. In order to provide insights for the robustness of our demonstrator, we performed resistance variation tests driving the heaters for 1000 cycles of 0-pi and measured resistance variations lower than 1% (in this case the source of the variation is unknown but likely coming from the vibration of the electrical probes employed in the test due to PAD material expansion with temperature). However, we are aware that a slight increment in the heater current (beyond the 2pi, leads to irreversible defects in the structure. Similar and deeper efforts need to be done by researchers and industry to provide data and tests of the technology maturity and readiness for industrialization and future commercialization.

In short, with state-of-the-art thermo-optic waveguides, improved efficiency is leading to a reduction of the electrical current demands and an increment of robustness during their dynamic operation (lower current densities lead to the mitigation of the electro-migration effect). However, greater efforts are required to quantify and qualify the robustness of phase tuning technology in general.

Reconfiguration speed and enhanced convergence of automated functions:

Reconfiguration speed:

Some of the automated configuration routines presented in this paper require a compilation time to configure the circuit for a certain functionality. Although some applications could be configured in run-time, the presented circuits perform signal processing after a slot of time dedicated to configuration. Most final applications would benefit from a reduction of the time consumed during the reconfiguration by increasing the speed and reducing the number of operations (processing, driving, and monitoring cycles).

In particular, one of the operations of the iterative configuration procedure could be divided as follows (See Supplementary Notes 1 and 4):

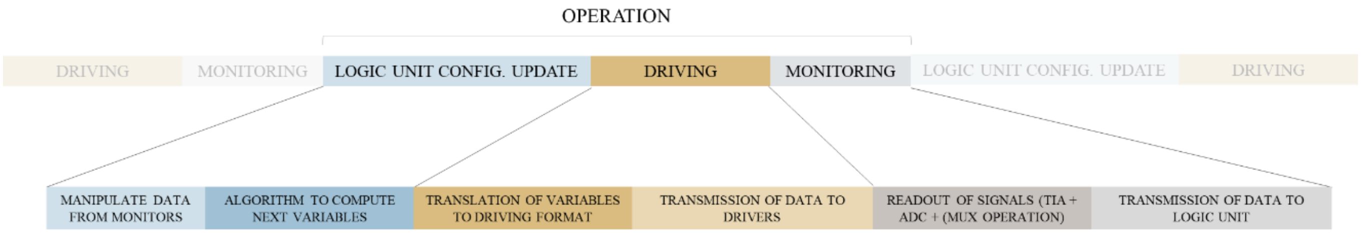

Supplementary Figure 48 | Division of the different stages in one of the operations of the self-configuration methods proposed in this work.

For each operation one can compute the next configuration settings based on the current setting and the readout monitoring data. This task requires the manipulation of the said signals and the execution of the optimization algorithm as described in Supplementary Notes 1 and 4. Next, we translate and transmit the next variables to the driving electronic circuitry. On a physical level, the response of the heater-based thermo-optic is limited to 2.2 µs, 5.6 and 65.5 µs for Ti-based heaters, silicon-doped heaters and Ti-based heaters with

under-etched waveguides, respectively [22]. Finally, the readout operation is done followed by an analog to digital operation and a transmission of the data to the logic unit for the next operation.

While processing times of the algorithm depend on the size of the array of variables (number of driving signals) and the hardware employed, the overall time is in the µs regime for vectors between 10 to 1000 variables and current electronic processors performance. In contrast, a significant delay can be imposed by the transmitted data between the logic unit, the driving circuitry, and the monitoring circuitry. To avoid a bottleneck in the internal transmission of data required by each operation, different protocols can be employed. For example, USB 2.0 allows theoretic rates up to 480 Mbit/s and USB 3.0 allows theoretic rates up to 4.8 Gbit/s, that would enable transferring the data for 1000 channels (driving of phase actuators) in 100 µs and 10 µs, respectively. Alternative protocols like PCIExpress can 3.x, 4.0, 5.0 or 6.0 provides better transfer rates up to few GB/s.

Moreover, some functions require the readout from different wavelength points (see example of optical filter). In this case, the system can employ either a tunable laser, a set of fixed lasers at different wavelengths or a broadband source plus a tunable passband filter. In most cases, the tuning speed is similar to the tuning of the phase shifter in the programmable photonic circuit.

All in all, applying some margins, we could assume that the total delay of an "operation" (setting computation, driving and monitoring) can be potentially done in the 20-200 µs regime (5-50 kHz). In general, moving to MHz or GHz regime, would require the use of alternative tuning mechanisms and the design and development of dedicated integrated electronics circuitry. Having said this in practice, with the exception of a handful of applications (most notably optical packet switching) there is not need for the reconfiguration speed to match the speed inherent in the dynamic properties of the optical signal.

Improved convergence:

Regarding the number of operations, further programming strategies and optimization methods can be employed. In this paper we suggested the combination of auto-routing algorithms and advance optimization methods to reduce the number of variables (driving phases) to be optimized during the self-configuration process. In addition, we are currently investigating alternative approaches to enhance the process convergence efficiency.

- Explore the use and combinations of different optimization methods: There is no free lunch in search and optimization algorithms and our underpinning work suggests that the use of orchestrated global search and local search algorithms can improve future scalability and convergence rates by one order of magnitude.
- Combine the autorouting algorithm and the optimization methods. Once the autorouting algorithm or the loading of dynamic (relative positioned) preset configurations is selected, we can perform the optimization method only on the variables selected by the first phase. This will reduce the overall number of variables and thus the search space without compromising the circuit flexibility.

- Select a portion of the system where optimization will be performed (only including certain areas or distances to minimize the number of variables and thus search space. We can select the two targeted optical ports. An autorouting algorithm can then select the shortest path between ports. Next, we can perform the advanced optimization methods (to the targeted function) only optimizing the programmable unit cells that are at a certain interconnection (logical) distance from that short-path.
- Employing Principal Component Analysis (PCA) algorithms to eliminate variables (phase shifters) with negligible impact during the optimization process to progressively reduce the number of variables during the optimization process.

As a further motivation and demonstration of the previous points, with the following example we can demonstrate the impact of the number of tuning variables on the operations required to achieve convergence. Precisely, we will configure a simple 1x2 beamsplitter following a simple cost function that includes the 50:50 splitting and the ripple in the optimization. The output feature 1 and output feature 2 are also defined to monitor the performance, average power at the outputs and average ripple, respectively. Supplementary Figure 49 (a) includes in red a path connecting the input with the two outputs. An autorouting example or a manual inspection of an advanced user would determine that these 10 TBUs (H6, H9, H16, H17, H15, H14, H13, H19, H23, H28) are enough (key) to build up the desired 1x2 splitter function. For the test, we perform the self-configuration method employing a PSO with the same hyperparameters as the one selected in the configuration of the optical filter example. First, we can include for the optimization process a single phase shifter for each of the selected TBUs, including 10 variables in the optimization process. We can define convergence as the point where the cost function becomes stable and the output features are around 4.2 dB and better than 0.5 dB, respectively. As illustrated in Fig. 49 (b), a fast configuration in less than 900 operations can be achieved. Next, we repeat the test including more tuning variables by employing the dual-drive configuration or by using additional unit cells. For the additional cells, we can employ sets of cells that are at a logical interconnection distance to the key path (in red), defining a set of unit cells at distance 1, 2 and 3. For comparison purposes, in Fig. 49 (b), we can see that convergence is achieved for the single-drive case of 34 variables (distance 3) at iteration 1500. In this case,

this application can be achieved without dual-drive mode as no-interferometry is fully required to achieve the targeted operation. If we include the dual-drive variables, we measured 1500 and 3000 approximated operations for the cases with 20 (key) and 68 (distance 3) variables, respectively. This proves the impact of the number of variables with the convergence rates.

Since these numbers can vary depending on the starting points and the passive phase offset of each unit cell, we ran a set of 30 independent examples with arbitrary phase offset distributions, including the different cases. The results are shown in Fig. 49 (c). Here we can observe an interesting behaviour. First, due to the cost function definition, the scenario where we optimize a reduced number of unit cells lead to sub-optimal results for the loss in the channel. Since we are considering 0.15-average loss per unit cell (See methods), we would expect an approximate optical power of 3 dB + 1.2 dB at the outputs. In a general case, the scenario with 10 unit cells (key) achieves faster convergence but suboptimal results (since it is not able to find alternative or improved paths). The more variables we include, the better performance we achieve on average and the slower convergence we achieve. We must highlight that the final performance is good for all cases. These results motivate the search of future methods of large scale circuits where the optimization is performed on a subset of the arrangement with reduced distance to the "key" cells selected by a basic auto-routing algorithm and the search of alternative optimizers.

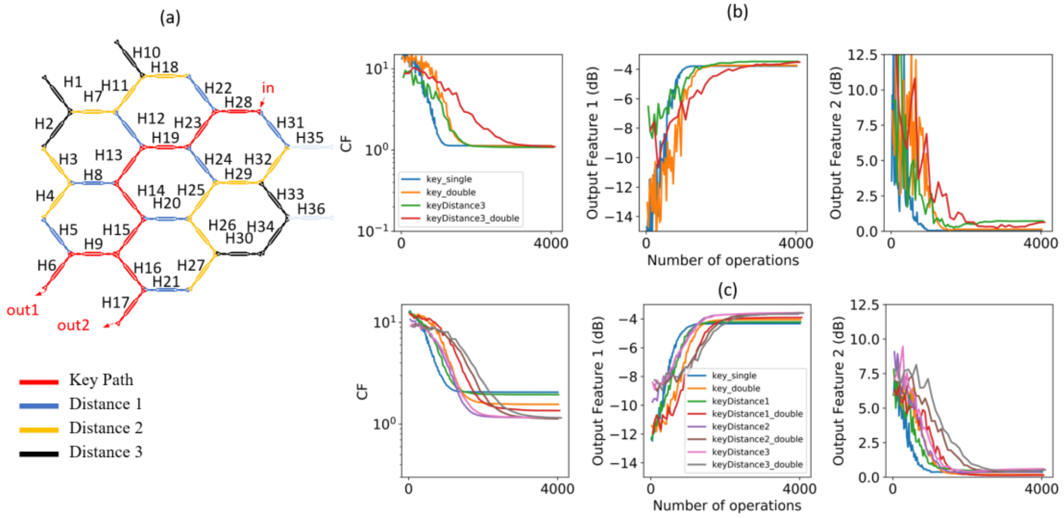

Supplementary Figure 49 | Number of variables vs convergence test: (a) waveguide mesh arrangement with the targeted circuit and TBUs highlighted by distance to the fundamental circuit. (key), (b) convergence test results for 1 case, illustrating the cost function and output feature 1 (average optical power at outputs, and output feature 2 (mean ripple at outputs). (c) convergence test for 30 independent samples per case.

In short, to further increase the reconfiguration speed of programmable photonic circuits, faster tuning methods would be required. However, in order to ensure the scalability of the circuits they must provide low loss (< 0.1 dB), low footprint and simple control electronics. In addition, achieving bit-rate reconfiguration times is not a strict requirement for a wide range of present and future applications. The range of applications of programmable photonics spans applications which are either analog in nature or do not require real-time digital signal processing.

Regarding the convergence rates of the proposed methods, the number of variables introduced during the optimization processes impacts on the performance and convergence rates of the system. Smarter strategies will be required to enable the self-configuration of large scale meshes at reasonable computation times and resources. As proposed in this work, the combination of both approaches (auto-routing, and optimization methods) can be employed to minimize the number of variables. Other techniques like alternative algorithms, combination with PCA, or dynamic variable selections can be employed to address future scalability limits. However, we believe that in the nearest future, the strongest limits will be coming from the evolution of the photonic (optical loss) and electronic (interfacing) hardware.

Resolution of the arbitrary responses of the reconfigurable optical filter

The unit cell length and the mesh interconnection topology set the rules of the interferometric structures that can be programmed.

In principle, as comprehensively detailed elsewhere [23], the hexagonal mesh is constrained to cavities of 6, 10, 12, 14, 16 TBUs and interferences of 2, 4, 6, 8, .. TBUs. In addition, other structures like delay lines, phase shifters, Sagnac loops, multiport interferometers, can be programmed as well. Employing different the FSRs would require the use of additional high performance blocks like Bragg gratings, higher-Q filters and larger FSR filters connected to the waveguide mesh arrangement.

Thus, the degree of arbitrary filter' shapes that can be done depends on the frequency region of interest and span, the hardware employed (shape and basic unit length) and the targeted application. In principle the combination of a large scale mesh with smaller TBUs can finally become an reconfigurable box of interferences with finer resolution and extended possibilities. However, reducing the size/length of the unit cell is a challenge that need to be addressed and that comes with additional trade-offs.

- Accumulated loss: the creation of large circuits involving a greater number of TBUs lead to more accumulated loss. This comes with the fact that the loss of the unit cell is dominated by the loss of the 3-dB couplers. If one need to program a cavity of 1 millimeter, we would require 6 TBUs with 166 um-TBUs or 20 TBUs if they measure 50 um. The second option goes through 28 directional couplers more than the first one. Even assuming 0.1 dB/coupler, we would be adding 2.8 dB more loss to our cavity, without considering additional bend-loss, phase-shifter loss (if any).
- Reducing the size/length of the TBU increase the integration density, which might find technology limits for the tuning crosstalk effect and the electrical interfacing of such a high-number of electrical connections.

Supplementary Figure 50 illustrates the relation between the Basic Unit Length and the achievable maximum Free Spectral Ranges for the different mesh topologies, assuming a silicon on insulator technology with a group index of 4.18.

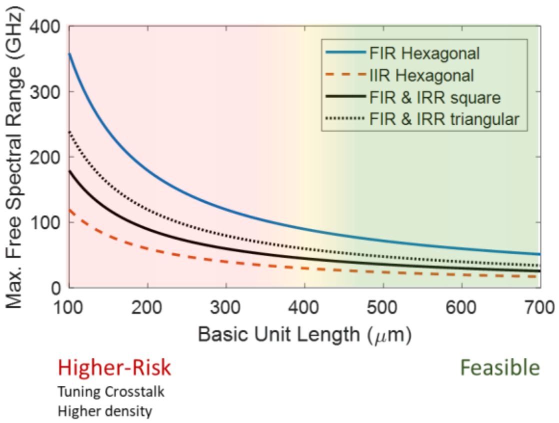

Supplementary Figure 50 | Comparative of maximum achievable Free Spectral Ranges for different waveguide mesh topologies assuming a silicon on insulator waveguide with 4.18 group index.

All in all, with the current state-of the art, the reconfigurable optical core based on waveguide mesh arrangements limits the circuits to discretized values determined by the length of the tunable basic unit. Increasing the resolution and reducing this limit, will require the miniaturization of the unit cell. With current state of the art, it can be achieved a reduction to around 100 um, employing suspended heaters of 30 um, ultra-short 3-dB couplers of 15 um and small transitions and access waveguides. To the best of our knowledge, these had not been reported yet and represent a future technology challenge.

The second key challenge that appears due to the minimization is the tuning crosstalk and the electrical interfacing of the phase shifters. In this paper, we demonstrated a solution for the first issue, as that even a severe 10% **arbitrary** tuning crosstalk can be addressed with the self-configuration methods proposed. For the packaging and electrical interfacing, additional technology efforts are required and expected during the next years.

Addressing FSR variations in interferometric structures

When synthesizing simple periodic interferometric structures, we expect a spectral response defined by a fixed (and single) free spectral range value. This does not seem to be the case of configs. 1 and 7 achieved by auto-routing technique in Fig.2 from main text, where distances between power notches are not equal. After a careful analysis, we believe that the rationale behind FSR deviations in this and other examples can be explained on account of the following facts:

- ***Accidental synthesis of spurious optical paths***: during the creation of any interferometric structure, we may be synthesizing additional undesired paths (i.e., interferometric structures) that introduce interferences with alternative FSR. Taking the synthesis of the aforementioned config. 1 as an example (ORR10), we could experience a contribution from an undesired MZI4 (TCs: H12, H22; short path: H16; long path: H11, H10, H15, H20, H21). Only a small deviation of the coupling factor from the TCs would suffice to introduce such deviation. Other structures are possible as well, since we used presets (from a pre-calibration stage) without including any re-adjustment or optimization of the resulting structure. In the case of Fig. 2 (main text), as we are using the auto-routing self-configuration scheme, tuning crosstalk is being neglected.

- ***Group index variation***: this issue is known to produce different FSRs at different wavelengths. However, such variation is quite soft and continuous in wavelength, and is typically appreciated in large wavelength spans. Indeed, this effect is behind the technique of measuring the group index vs wavelengths employing different structures such as unbalanced MZIs. In our case, the group index of the programmed waveguides employing unit cells might be subject of variations versus wavelength, since a large portion of the circuit is experiencing a change in their refractive indexes. However, again, this FSR change would be appreciated for a larger span than the one shown in the examples, and would show a softer and continuous FSR variation with wavelength. This effect could be discarded in this case with a 0.3 nm span.
- ***System resolution***: As mentioned in Supplementary Note 9, measuring interferometric structures with small FSRs does require a considerable measurement resolution. If not enough points are employed, we might not be able to resolve the spectral trace optimally. As shown in Supplementary Figure 51, this scenario can lead to having a trace where those points closer to the notch are shifted, producing their own contribution to the FSR error. In config. 1 (main text), for example, we used approximately 50 points per FSR and the system resolution is 1 pm, which points to a maximum resolution related deviation of up to +/- 2 pm range approximately -in such case, both notches of a period would suffer a maximum deviation of 1 pm in opposite directions. Again, this is a low-probability, low-impact issue for this precise example.

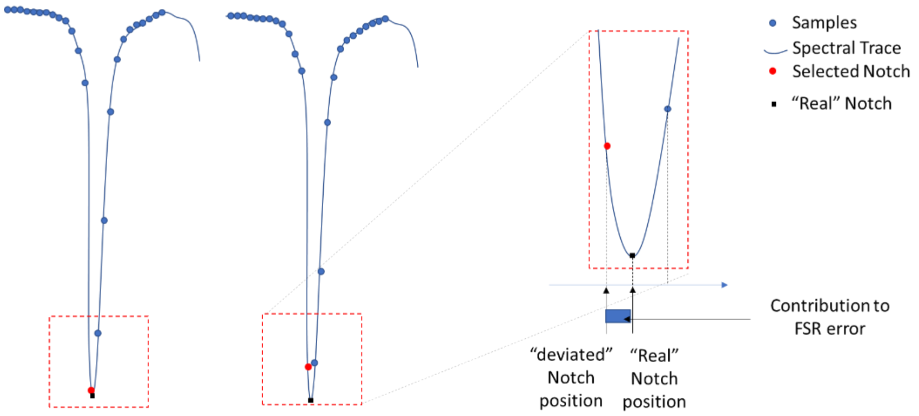

Supplementary Figure 51 | Example of notch mismatch due to insufficient system resolution

- ***Synchronization issues***: During the experiments, we employed a Laser ANDO + OSA ANDO synchronized tunable laser source system to achieve high-resolution and employ the existing synchronization commercial toolbox as motivated in Supplementary Note 9. However, sometimes, during few measurements we experienced that the OSA employed interrupts the measurement for different causes (fixing synchronization issues with the laser unit, fixing synchronization issues with the PC, liberate memory, transmit and perform internal data management, etc). During that time, the photonic system sometimes can behave dynamically altering the system's performance. This dynamic behavior occurs due to the non-optimal overall thermal management implemented for the system (Peltier-cell + Thermal Control Unit + suboptimal thermal holder that allows a residual thermal propagation between the heatsink and the chip holder) and with less impact, from the actual phase shifters and tuning crosstalk. When the system continued the measurements, from the previous wavelength point and a few seconds later, the overall chip temperature produced a slight shift on the spectral response, modifying locally the FSR of the notch nearby.

Although all the aforementioned possibilities can occur at the same time, we believe that the last one (synchronization issues during undesired dynamic behavior) is the one having a stronger impact in this work. To further study it, apart from previous measurements, we extracted the position of the notches (analytical and measured) of the different measurements included employing the auto-routing self-configuration (Fig. 2 main text) for its comparison, as represented below in Supplementary Figure 52. We observe that, indeed, there is only a significant difference (larger than 1 pm, the resolution of our OSA) in configs. 1 and 7. In all remaining scenarios, any other difference lies below or around such number. Note that current examples employ information from a pre-calibrated process and neglect the behaviour of the optical and tuning crosstalks.

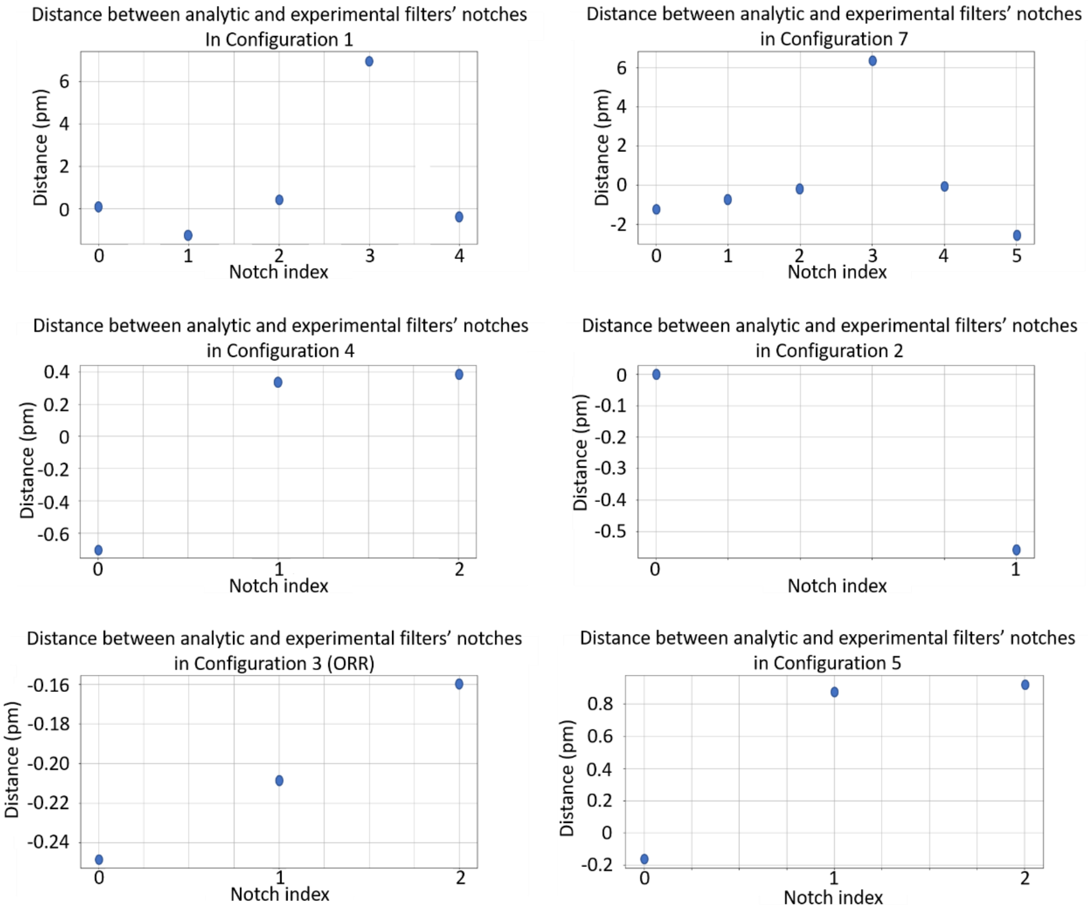

Supplementary Figure 52 | Distances (in pm) between analytic and experimental filters' notches from Fig.2 of main text. In all cases (except for configs. 1 and 7) every notch distance lies below 1 pm, the system resolution of our OSA. Note that the y-axis has a different span for each figure.

For all experimental configurations employing optimization based self-configuration (Figure 5 main text and Supplementary Figures 44, 45 and 46 from this supplementary material), the difference between final results employing almost any configuration and the spectral masks used to achieve them is also hardly noticeable. Precisely, as we used 101 points and a span of 0.5 nm to represent the first two ones, their spectral resolution is therefore of 5 pm. In those cases, only results depicted in green in Supplementary Figure 44 differ in one unit in x-axis (5 pm) from those from the spectral mask at 1584.95 nm, while the rest of them -and of the remaining experiments- match exactly with the mask notch point. In the third circuit, whose spectral resolution was of 301 points, we found only one notch mismatch of 1.67 pm (again, one unit in x-axis) at 1585.045 nm. Note that the last example in Fig 5 Main text corresponds to the ORR10 that showed the FSR deviation in one of its notches. In this case the result is matching the expected FSR.

To mitigate all previously mentioned events, several solutions can be provided:

- Auto-routing based self-configurations would benefit from a posterior fine tuning to match the targeted functionality and further correct tuning and optical crosstalks) or by including optical crosstalk and tuning crosstalk during the pre-characterization. Since after the auto-routing we achieved a solution valid or close to the optimal working configuration, the use of local-search algorithms should also provide a faster final solution.
- The optimization process could and should repeat the measurement if a sync issue is detected. This would alleviate the synchronization issues. Although synchronization issues are given sporadically by our current synchronized TLS-system, a future tunable laser + array of power detectors could potentially experience similar synchronization errors.
- If we assume that the FSR misalignment is coming from the *spurious path, group index variation, or resolution issue* cause, additional/different features can be incorporated to the cost function to further alleviate /optimize/minimize the deviation of the FSR. This can be done following the principles of [11], where some features like the Extinction Ratio or the IL are incorporated to the cost function. In this case, at each operation, we can post process the spectral trace to extract the Extinction Ratio of the peaks separated by a specific FSR (withing a certain margin). Solutions to mitigate similar issues and/or potentially improve the convergence of different applications can range to infinite possibilities of cost-function tailoring, where additional features are considered and added. We expect that this work will motivate application specific experts (optical filter designers,

optical beamformer designers, optical switching designers, …) to tailor and propose alternative cost functions to achieve faster reconfigurations with higher final quality. As a final example consider the mentioned case of the optical filter. The cost function to minimize could be represented as:

$$CF = f_{mask} + f_{ER} + f_{Ripple},$$
$$f_{mask} = c_1 \cdot m.s.e.(mask, trace),$$

Where m.s.e is the mean square error between the mask and the spectral trace, f_{ER} search for the peaks separated a certain FSR (within a margin) and maximizes their ER, and f_{Ripple} search for the peaks within the passbands and minimize the ripples. Note that every feature comes with a weight (c1, c2, c3, …) that helps the designer to equalize or priorate some features before others. In addition, a feature considering the minimization of the sum of power at a few strategic output ports can lead to the minimization of the *spurious path* problems, as they generate undesired optical power splitting that circulates all over the mesh. Consider that the features to be incorporated also depends on the type of the filter (passband, stopband, etc, …). Finally, a feature that can potentially improve the convergence of the self-configuration process is the minimization of the optical power at certain residual ports. This helps to focus most of the energy to the desired output ports.

- Once the structure is close to the targeted value (as in the examples in the paper) one could additionally employ fitting techniques to the targeted analytical function to alleviate any measurement resolution related issue.

Supplementary Note 11. Examples of recent demonstrations of reconfigurable waveguide mesh arrangements

Supplementary Table 24: Experimental demonstrators of feed-forward meshes/ multiport interferometers [16] TBU: Number of TBU, PS: Number of phase shifters, OP: Number of optical ports, P: Power Consumption, RT: Response Time, CL: Fiber-chip coupling losses, E: edge-coupling, V: vertical-coupling

Year	Authors	TBU	PS	OP	dB /TBU	TE	Foundry	P (mW) / π	RT (us)	Topology	Size (mmxmm)	PS density (1/mm^2)	CL/facet (dB)	Chip-couplers
2016	Carollan, et. al	15	30	12	TBD	TO	Silica on silicon	400	TBD	Triangular	100x40	0.007	0.5	E
2016	Ribeiro, et. al.	9	18	8	1	TO	Silicon	15	250	Triangular	1x3	6.000	TBD	V
2017	Anoni, et. al.	6	12	8	1	TO	Silicon	10	10	Triangular	3.7 x 1.4	2.320	TBD	V
2017	Harris, et. al.	88	176	52	TBD	TO	Silicon	10	8	Trapezoid	2.1 x 4.3	19.050	3.5	E
2018	Mennea, et. al	30	120	40	4.20	TO	Silica on silicon	10 V (PWM)	TBD	Rectangular	3.05x1.9(x3)	20.710	0.8	E
2018	Caterina et al.	64	128	16	0.27	TO	Silicon nitride	3.35 V	TBD	Triangular (double)	16x16 TBD	0.500	2.9	E
2019	Zhou, et. al.	20	48	8	TBD	TO	Silicon	27	TBD	Triangular SVD	1.3 x 7.5	4.920	3.5	V
2019	Zheng et al	10	18	10	0.63	TO	Silicon nitride	330	>1000	Rectangular	11x3	0.550	3.5	E

Supplementary Table 25: Experimental demonstrators of feed-forward / feed-backward (General-Purpose) meshes/ multiport interferometers [16] TBU: Number of TBU, PS: Number of phase shifters, OP: Number of optical ports, BUL: Basic Unit Length, BUD: Basic Unit Delay, TE: Tuning effect, TO: Thermo-optic, P: Power Consumption, RT:

Year	Authors	TBU	PS	OP	dB/ TBU	BUL (um)*1	BUD (ps)*2	TE	Foundry	P (mW) /pi	RT (us)	Topology	TBU	Size (mmxmm)	TBU density (1/mm2)	C (CL)
2015	Zhuang, et. al.,	7	14	4	TBD	3450	19.7	TO	Silicon nitride	300	TBD	Square	MZI	3.5x8.5	0.235	E(TBD)
2016	Pérez, et. al.,	30	60	24	0.59	975	13.5	TO	SOI	110	TBD	Hexagonal	MZI	15x15	0.133	V(6.5)
2017-2020	Pérez, et. al.[25]	40	80	30	TBD	1315	8.4	TO	Silicon nitride	290	TBD	Hexagonal	MZI	11x5.5	0.661	E (3.5)
2018	Pérez, et. al.	5	10	8	TBD	1178	TBD	TO	Silicon nitride	300	TBD	Triangular	DD-DC	7x2.5	0.285	E (3.5)

Supplementary References

[1] D. Pérez and J. Capmany, "Scalable analysis for arbitrary photonic integrated waveguide meshes," Optica, vol. 6, no. 1, pp. 19-27, 2019.
[2] D. Pérez, "Field-Programmable Photonic Array for multipurpose microwave photonic applications," in IEEE International Topical Meeting on Microwave Photonics (MWP), Ottawa, Canada, 2019.
[3] D. Pérez et al., "Multipurpose silicon photonics signal processor core," Nature communications, vol. 8, no. 636, 2017.
[4] D. Pérez et al., "Switching and cross-talk characteristics of compact thermal tuners on a Silicon Nitride platform," in European Conference on Integrated Optics (ECIO), Warsaw (Poland), 2016.
[5] S. Yegnanarayanan, et al., "Automated Initialization of Reconfigurable Silicon-Nitride (SiNx) Filters," in Conference on Lasers and Electro-Optics, San José, EEUU, 2018.
[6] M. Milanizadeh, et al., "Canceling Thermal Cross-Talk Effects in Photonic Integrated Circuits," Journal of Lightwave Technology , vol. 37, no. 4, pp. 1325-1332, 2019.
[7] A. López, et al., "Auto-routing algorithm for field-programmable photonic gate arrays," Optics Express, vol. 28, no. 1, pp. 737-752, 2020.

[8] X. Chen, W. Boggaerts, “A Graph-based Design and Programming Strategy for Reconfigurable Photonic Circuits,” in IEEE Photonics Society Summer Topical Meeting Series (SUM), Fort Lauderdale, 2019.
[9] D. Whitley, “A genetic algorithm tutorial,” Statistics and Computing, vol. 4, p. 65–85, 1994.
[10] J. K. R. Eberhart, “A new optimizer using particle swarm theory,” in MHS'95. Proceedings of the Sixth International Symposium on Micro Machine and Human Science, Nagoya, Japan, Japan , 1995.
[11] D. Pérez, “Programmable Integrated Silicon Photonics Waveguide Meshes: Optimized designs and control algorithms,” Journal of Selected Topics in Quantum Electronics, special issue on silicon photonics, vol. 26, n. 2, 2019.
[12] I. Goodfellow, Y. Bengio and A. Courville, Deep Learning, Boston: MIT Press, 2016.
[13] C.K. Madsen, J. H. Zhao, Optical Filter Design and Analysis: A Signal Processing Approach, Wiley-Interscience, 1999.
[14] Y. Xie, et al., “Picosecond optical pulse processing using a terahertz-bandwidth reconfigurable photonic integrated circuit,” Nanophotonics, pp. https://doi.org/10.1515/nanoph-2017-0113, 2018.
[15] M. Burla, Advanced integrated optical beam forming networks for broadband phased array antenna systems, Telecommunication Engineering Faculty of Electrical Engineering, Mathematics and Computer Science, 2013.
[16] J. Capmany, D. Pérez, Programmable Integrated Photonics, Oxford University Press , 2020.
[17] F. Morichetti, A. Canciamilla, C. Ferrari, M. Torregiani, A. Melloni, and M. Martinelli, “Roughness induced backscattering in optical silicon waveguides,” Phys. Rev. Lett., vol. 104, no. 3, pp. 1–4, 2010.
[18] J.V. Capenhout, “2×2siliconelectro-optic switch with 110-nm bandwidth for broadband reconfigurable optical networks,” Optics Express, vol. 17, no. 26, (2009).
[19] J. C. Mikkelsen. et. al., “Dimensional variation tolerant silicon-on-insulator directional couplers”, Optics Express vol. 22, no. 3, pp. 3145-3150, (2014).
[20] A. Masood et al., "Comparison of heater architectures for thermal control of silicon photonic circuits," 10th International Conference on Group IV Photonics, Seoul, 2013, pp. 83-84.
[21] D. Pérez, I. Gasulla and J. Capmany, "Toward Programmable Microwave Photonics Processors," in Journal of Lightwave Technology, vol. 36, no. 2, pp. 519-532, 15 Jan.15, 2018, doi: 10.1109/JLT.2017.2778741.
[22] Maxime Jacques, Alireza Samani, Eslam El-Fiky, David Patel, Zhenping Xing, and David V. Plant, "Optimization of thermo-optic phase-shifter design and mitigation of thermal crosstalk on the SOI platform," Opt. Express 27, 10456-10471 (2019)
[23] D. Pérez et al., “Reconfigurable lattice mesh designs for programmable photonic processors," Opt. Express 24, 12093-12106 (2016)
[24] D. Pérez PhD Thesis, “Integrated microwave photonic processors using waveguide mesh cores”, 2017.
[25] D. Pérez, A. Gutierrez, J. Capmany, “Microwave Photonics Filters Using a Silicon Nitride Programmable Processor with Folded Heaters”, to be presented at IEEE International Topical Meeting on Microwave Photonics (MWP), Japan, 2020.
